# Anti-vascular endothelial growth factor therapy for age-related macular degeneration: a systematic review and network meta-analysis

**DOI:** 10.1186/s13643-021-01864-6

**Published:** 2021-12-20

**Authors:** Andrea C. Tricco, Sonia M. Thomas, Erin Lillie, Areti Angeliki Veroniki, Jemila S. Hamid, Ba’ Pham, Taehoon Lee, Arnav Agarwal, Jane P. Sharpe, Alistair Scott, Rachel Warren, Ronak Brahmbhatt, Erin Macdonald, Ghayath Janoudi, Rajeev H. Muni, Carolina L. M. Francisconi, Trevor Richter, Sharon E. Straus

**Affiliations:** 1 Knowledge Translation Program, Li Ka Shing Knowledge Institute, St. Michael’s Hospital, Unity Health Toronto, 209 Victoria Street, East Building, Toronto, ON M5B 1T8 Canada; 2 Epidemiology Division and Institute for Health Policy, Management, and Evaluation, Dalla Lana School of Public Health, University of Toronto, 155 College St Room 500, Toronto, Ontario M5T 3M7 Canada; 3 Queen’s Collaboration for Health Care Quality Joanna Briggs Institute Centre of Excellence, School of Nursing, Queen’s University, 99 University Ave, Kingston, Ontario K7L 3N6 Canada; 4 Department of Primary Education, School of Education, University of Ioannina, 455 00 Ioannina, Mpizani Greece; 5 Institute of Reproductive and Developmental Biology, Department of Surgery & Cancer, Faculty of Medicine, Imperial College, London, Exhibition Rd, South Kensington, London, SW7 2BU UK; 6 Department of Mathematics and Statistics, University of Ottawa, 150 Louis-Pasteur Pvt, Ottawa, ON K1N 6N5 Canada; 7 Department of Clinical Epidemiology and Biostatistics, McMaster University, 1280 Main Street West, Hamilton, Ontario L8S 4K1 Canada; 8 Department of Medicine, University of Toronto, 1 King’s College Circle, Toronto, Ontario M5S 1A8 Canada; 9 Institute of Health Policy, Management and Evaluation, University of Toronto, 6th floor, 155 College Street, Toronto, Ontario M5T 3M7 Canada; 10 Canadian Agency for Drugs and Technologies in Health (CADTH), 865 Carling Avenue, Ottawa, Ontario K1S 5S8 Canada; 11 St. Michael’s Hospital/Unity Health Toronto, Department of Ophthalmology and Vision Sciences, University of Toronto, Toronto, Canada; 12 Department of Geriatric Medicine, University of Toronto, 27 King’s College Circle, Toronto, Ontario M5S 1A1 Canada

**Keywords:** Ranibizumab, Bevacizumab, Aflibercept, Conbercept, Brolucizumab, Anti-vascular endothelial growth factor, Age-related macular degeneration

## Abstract

**Background:**

The comparative safety and efficacy between anti-vascular endothelial growth factor agents (anti-VEGFs) and between combined therapies for patients with neovascular age-related macular degeneration (nAMD) is unclear. We conducted a systematic review to examine the comparative safety and efficacy anti-VEGFs for adults with nAMD.

**Methods:**

Studies were identified through MEDLINE, EMBASE, and Cochrane CENTRAL (inception to June 3, 2019), grey literature, and scanning reference lists. Two reviewers independently screened citations and full-text articles to identify randomized controlled trials (RCTs), extracted data, and appraised risk of bias. Pairwise random-effects meta-analysis and Bayesian network meta-analysis (NMA) were conducted. The primary outcomes were the proportion of patients experiencing moderate vision gain (≥ 15 letters on the Early Treatment Diabetic Retinopathy Study chart) and the proportion of patients experiencing moderate vision loss (≤ 15 letters).

**Results:**

After screening 3647 citations and 485 potentially relevant full-text articles, 92 RCTs with 24,717 patients were included. NMA (34 RCTs, 8809 patients, 12 treatments) showed small differences among anti-VEGFs in improving the proportion of patients with moderate vision gain, with the largest for conbercept versus broluczumab (OR 0.15, 95% CrI: 0.05–0.56), conbercept versus ranibizumab (OR 0.17, 95% CrI: 0.05–0.59), conbercept versus aflibercept (OR 0.19, 95% CrI: 0.06–0.65), and conbercept versus bevacizumab (OR 0.2, 95% CrI: 0.06–0.69). In NMA (36 RCTs, 9081 patients, 13 treatments) for the proportion of patients with moderate vision loss, small differences were observed among anti-VEGFs, with the largest being for conbercept versus aflibercept (OR 0.24, 95% CrI: 0–4.29), conbercept versus brolucizumab (OR 0.24, 95% CrI: 0–4.71), conbercept versus bevacizumab (OR 0.26, 95% CrI: 0–4.65), and conbercept versus ranibizumab (OR 0.27, 95% CrI: 0–4.67).

**Conclusion:**

The only observed differences were that ranibizumab, bevacizumab, aflibercept, and brolucizumab were statistically superior to conbercept in terms of the proportion of patients with nAMD who experienced moderate vision gain. However, this finding is based on indirect evidence through one small trial comparing conbercept with placebo. This does not account for drug-specific differences when assessing anatomic and functional treatment efficacy in variable dosing regimens.

**Systematic review registration:**

PROSPERO registration number CRD42015022041.

**Supplementary Information:**

The online version contains supplementary material available at 10.1186/s13643-021-01864-6.

## Background

Age-related macular degeneration (AMD) has been identified as one of the leading causes of blindness in older adults globally [1–4]. Neovascular AMD is characterized by choroidal neovascularization, subretinal fluid, haemorrhage and fibrosis [5]. First-line treatment for neovascular AMD includes anti-vascular endothelial growth factor (anti-VEGF) agents, including aflibercept, ranibizumab, and bevacizumab [6]. These agents block VEGF-A isoforms and inhibit VEGF-driven vascular permeability and neovascularization [7].

As newer anti-VEGF agents (conbercept, brolucizumab) become available, there is a need to assess the comparative safety and efficacy between existing anti-VEGF agents and combined therapies for patients with neovascular AMD. Previous reviews included only 3–4 different interventions (bevacizumab, ranibizumab, pegatanib, verteporfin), and did not look at other existing treatment options or combinations of treatments [8, 9]. The majority of these reviews conducted pairwise meta-analysis, which limits them to the direct comparison of two interventions.

Network meta-analysis (NMA) is a statistical approach that allows one to compare two or more interventions simultaneously and rank them on the totality of the evidence [10]. The advantage of NMA is that it combines direct and indirect evidence. The inclusion of indirect evidence enables one to statistically compare interventions that have never been directly compared, which is the case for many of the anti-VEGF agents.

We conducted a systematic review and NMA examining the relative safety and efficacy of anti-VEGF agents compared with other treatments for patients with neovascular AMD.

## Methods

### Protocol

The Preferred Reporting Items for Systematic reviews and Meta-analyses for Protocols (PRISMA-P) was used to develop the protocol [11]. Feedback was obtained from the research team, as well as clinical experts, and members of the commissioning agency (Canadian Agency for Drugs and Technologies in Health, CADTH). The protocol was registered with PROSPERO (CRD42015022041) (Additional file 1: eAppendix 1). This systematic review is related to a therapeutic review, which was conducted on anti-VEGF agents for four ophthalmology indications [12]. The therapeutic review was conducted for the Canadian Drug Expert Committee, which is a pan-Canadian advisory board that makes recommendations regarding drugs listing to federal, provincial, and territorial publicly funded drug plans. We reported our results using the PRISMA-NMA (Additional file 1: eAppendix 2).

### Eligibility criteria

Parallel or cluster randomized clinical trials (RCTs) of patients aged 50 years or older with neovascular AMD were included. Interventions of interest were intravitreal injection of anti-VEGF agents (aflibercept, bevacizumab, ranibizumab, brolucizumab, or conbercept), alone or in any combination. Comparators were anti-VEGF agents compared to each other, photodynamic therapy with verteporfin (PDT), corticosteroids (intravitreal injection or implant: triamcinolone acetonide (IVTA), dexamethasone implant (DXM), fluocinolone acetonide implant), and laser photocoagulation. Other treatments for neovascular AMD, such as interferon alfa [13], radiotherapy [14], or ginkgo biloba [15] and pegatanib [16], were excluded because they were not ophthalmological therapy or were no longer recommended [13–15, 17, 18].

The outcomes were selected by clinical experts affiliated with CADTH and defined in Additional file 1: eAppendix 3. The primary outcome measures consisted of the proportion of patients experiencing vision gain of ≥ 15 letters on the Early Treatment Diabetic Retinopathy Study (ETDRS) chart and vision loss of ≥ 15 ETDRS letters. Secondary outcome measures were: difference in mean change in best-corrected visual acuity (BCVA) from baseline in ETDRS letters, legal blindness, vision-related function, all-cause mortality, arterial and/or venous thromboembolic events (ATE or VTE), bacterial endophthalmitis (BE), increased intraocular pressure, retinal detachment, adverse events (AE), serious AE, and withdrawals due to AE. Due to limited resources, only papers written in English were included.

### Information sources

The electronic databases included: MEDLINE, EMBASE, and the Cochrane Central Register of Controlled Trials. The main literature search was supplemented by searching for RCTs online [19] and scanning the reference lists of included RCTs.

### Literature search

An experienced librarian drafted the literature search, which was peer-reviewed by another using the PRESS checklist [20]. The final literature search strategy was updated on June 3, 2019 (Additional file 1: eAppendix 4).

### Screening process

The team reached 78% agreement after 2 pilot tests of the eligibility criteria using 50 citations each. Following this calibration exercise, pairs of reviewers (AS, AA, EL, JA, MK, ST, TL) screened titles and abstracts independently. For screening potentially relevant full-text papers pairs of reviewers (AS, AA, EL, JA, MK, ST, TL) screened all full-text articles independently after 70% agreement was reached on pilot tests with 20 articles. All screening was conducted using the Synthesi.SR online systematic review software [21].

### Data extraction process

After the team reached approximately 75% agreement on a pilot-test of the data extraction form on 5 RCTs, pairs of reviewers (AA, AS, BP, EL, EM, GJ, JA, JS, RB, RW, ST, TL) conducted all abstraction independently. All data were confirmed by a third reviewer (EL or ST).

### Data items

Data were collected on patient characteristics (e.g., mean age) and study characteristics (e.g., sample size). All outcome results were abstracted for the longest duration of follow-up [22]. Multiple publications reporting data from the same patients were sorted into the main paper and companion reports, with companion reports used for supplementary data only [23].

### Risk of bias assessment

Using the same process for data abstraction, the Cochrane risk-of-bias tool was used for risk of bias assessment [24]. In addition, the results from the network meta-analyses of our primary outcomes were assessed using the Confidence in Network Meta-Analysis (CINeMA) framework [25, 26].

### Data analysis

For all outcomes with at least 2 direct comparative studies available, pairwise random-effects meta-analysis was conducted in a Bayesian environment. The odds ratio (OR) was used for dichotomous outcomes. Studies reporting zero events across all arms were excluded from the analysis. The mean difference (MD) was used for continuous outcomes. If studies used different scales to measure BCVA, they were converted to approximate ETDRS letter scores and standard deviations [27, 28]. If necessary, standard deviations were imputed using established methods [29, 30]. The mean control event rate across included studies was calculated for each outcome when possible. A significant finding was defined as an estimate with a 95% credible interval that excludes the null.

Clinicians on the team selected the treatment nodes focused on the recommended dosing according to Health Canada (Additional file 1: eTable 1). Whenever the evidence formed a connected network diagram, a random-effects Bayesian NMA was conducted in OpenBUGS (Version 3.2.3 rev 1012) [31]. We assumed heterogeneity between studies using a common within-network between-study variance (*τ*^2^) across treatment comparisons, as the included treatments were of a similar nature. An informative prior was used for the between-study variance across all analyses of binary outcomes based on those recommended by Turner et al. [32]. We selected the priors suggested for semi-objective outcomes and pharmacological treatments versus placebo comparison type for the outcomes of vision gain, vision loss, AE, ATE, blindness, withdrawals due to AE, and retinal detachment. Suggested priors for all-cause mortality outcomes and pharmacological treatment vs placebo comparison type were used for the outcome of mortality, and priors suggested for semi-objective outcomes and pharmacological versus pharmacological treatment comparison type were used for the outcomes of serious AE and VTE. Vague priors were used for the between-study variance across analyses for continuous outcomes as the mean difference was determined to be the most appropriate effect measure for the data, and to our knowledge, there are no informative priors for this effect measure and the underlying outcomes of interest. Median effect sizes and 95% credible intervals (CrIs) were calculated using the Markov Chain Monte Carlo (MCMC) method. We ran two chains with 100,000 draws (or until convergence) and removed the first 10,000 (burn-in). A thinning for every 10 draws was used to reduce autocorrelation. Convergence was assessed by visually inspecting history and trace plots. Binary outcomes were modelled using a binomial distribution and continuous outcomes using a normal distribution. The 95% predictive interval (PrI) was calculated to predict the interval within which the results of a future study may lie [33]. The design-by-treatment model was used in STATA to examine consistency in each NMA [34]. The ranking of treatments were explored using Surface Under the Cumulative RAnking (SUCRA) curves [35] with their respective 95% CrIs [36] and plotted using the rank-heat plot [37]. The comparison-adjusted funnel plot was drawn for each NMA, ordering the treatments chronologically based on when they appeared in the Canadian market [38], to examine potential publication bias and small-study effects.

Additional analyses were examined to examine robustness of results when there were more than 10 studies and when the number of studies included in the analysis was greater than the number of treatments: meta-regression on study duration; sub-group analyses on study duration (12 months versus 24 months; if meta-regression revealed significant association), percentage of patients with hypertension (0 versus ≥ 40%), and lens status of patients (phakic/pseudophakic versus cataract). These study and patient characteristics were presented in network plots for each treatment comparison to verify that the transitivity assumption was upheld. We completed several sensitivity analyses including restricting our NMAs to the following: studies with a low risk of bias on random sequence generation; studies with a low risk of bias on allocation concealment; and, large RCTs (> 100 patients) to surmount small study effects. To estimate treatment dose effects, the hierarchical model (i.e. exchangeable subnodes model) with subnode consistency was applied [39].

## Results

### Literature search

**Fig. 1:**
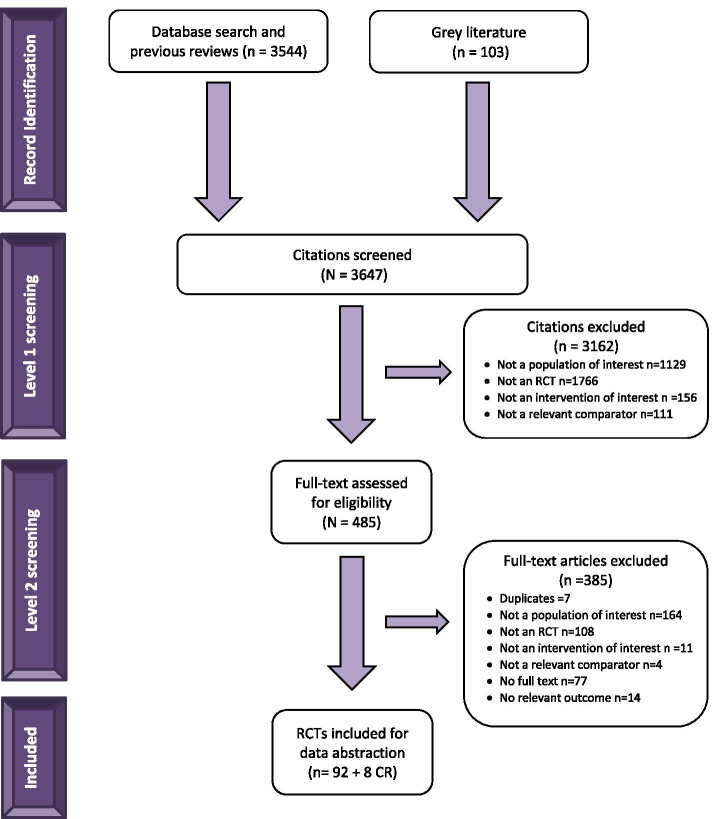
Flow diagram of included studies. Flow diagram illustrating how included studies were identified during screening of citations and full text articles

After screening 3,647 titles and abstracts and 485 potentially relevant full-text articles, 92 RCTs plus 8 companion reports were included (Fig. 1). The full citations can be found in Additional file 1—References. Five of the included RCTs were unpublished studies with data posted on clinicaltrials.gov [40–44]. We contacted 12 authors and received a response from five (response rate: 42%), but this did not lead to inclusion of any additional data or studies.

### Study and patient characteristics

The RCTs were published between 2000 and 2019 (Table 1; Additional file 1: eTables 2-3). Most studies were conducted in Europe (36%) and North America (32%). One of the included RCTs was a cluster-randomized trial (which was subsequently excluded from analysis as the same intervention was administered in both arms), while the rest were randomized trials at the patient level. The average duration of follow-up was 12.55 months. The most common intervention evaluated across the included studies was ranibizumab (53.2%). Across the studies, the average age of patients with neovascular AMD was between 60 and 83 years (Table 1) and the proportion of women was 56%.

**Table 1:** Study and patient characteristics

		Number of studies (***n*** = 92)	% of studies
**Study characteristics**			
**Year of publication**	2000–2006	8	8.7%
	2007–2011	31	33.7%
	2012–2017	42	45.7%
	2018–2019	11	11.9%
**Geographic region**	Europe	33	35.9%
	North America	29	31.5%
	Asia	19	20.7%
	Multi	8	8.7%
	Australia/New Zealand	3	3.3%
**Study design**	Parallel RCT	91	98.9%
	Cluster RCT	1	1.1%
**Setting**	Multi-centre	55	59.8%
	Single-centre	36	39.1%
**Sample Size**	< 50	25	30%
	50–149	31	33%
	150–249	9	11%
	250–499	13	9%
	500–999	8	4%
	≥ 1000	14	15.5%
**Study duration (months)**^a^	< 12	18	19.6%
	12	58	63.0%
	13–23	4	4.3%
	24	10	10.9%
	36	2	2.2%
**Frequency of interventions examined**	Aflibercept	5	6%
	Bevacizumab	27	34%
	Bevacizumab+IVTA+PDT	1	1%
	Bevacizumab+PDT	5	6%
	Brolucizumab	2	2%
	Conbercept	1	1%
	DXM	1	1%
	DXM+PDT+ranibizumab	1	1%
	DXM+ranibizumab	3	4%
	IVTA	4	5%
	IVTA+Bevacizumab	2	3%
	IVTA+PDT	7	9%
	IVTA+ranibizumab	1	1%
	PDT	15	19%
	PDT+ranibizumab	10	13%
	Placebo	8	10%
	Ranibizumab	42	53%
**Number of studies by outcome**	Vision gain	48	61%
	Vision loss	51	65%
	Mean BCVA	77	97%
	Legal blindness	8	10%
	Vision-related function	6	8%
	All-cause mortality	45	57%
	Arterial thromboembolic events	18	23%
	Venous thromboembolic events	11	14%
	Bacterial endophthalmitis	24	30%
	Retinal detachment	20	25%
	AE	22	28%
	Serious AE	23	29%
	Withdrawals due to AE	16	20%
**Patient characteristics**			
*Total # patients: 24,717* *Mean number of patients (range): 655.77 (7–4300)* *Mean age in years (range): 75.3 (60.0–83.0)*^*a*^ *% Female (range): 0.0–74.0%*			
**Mean age (years)**^b^	60-70	3	4%
	70-75	7	9%
	75-80	21	27%
	> 80	1	1%
	NR	45	57%
**% female**	< 47.0%	14	18%
	48.0–57.0%	13	17%
	58.0–62.0%	19	24%
	62.5–65.0%	11	14%
	66.0–74.0%	13	16%
	NR	9	11%
**% patients with hypertension**	0.0–50.0%	4	5%
	51.0–78.0%	3	4%
	NR	72	91%
**Lens status**	Mixed	6	8%
	Pseudophakic	4	5%
	Phakic	1	1%
	NR	79	87%

*Abbreviations: AE*, adverse events; *BCVA*, best-corrected visual acuity; *DXM*, dexamethasone; *IVTA*, intravitreal triamcinolone; *NR*, not reported; *PDT*, photodynamic therapy; *RCT*, randomized controlled trials

^a^Average study duration = 12.38 months

^b^Two studies reported a median age of 69 (Li, 2012) and 79 (Felgen, 2017) years respectively

### Risk of bias results

Approximately 76% of the RCTs were assessed as having a high or unclear risk of bias due to random sequence generation and 84% had an unclear risk of bias due to allocation concealment (Additional file 1: eFigure 1, eTable 4). Approximately half were at an unclear risk of bias due to selective reporting, as well as other (e.g., funding) bias.

### Statistical analysis results

Across all analyses, the transitivity assumption was upheld after visually assessing the distribution of effect modifiers (Additional file 1: eTable 5). There was no evidence of inconsistency according to the design-by-treatment interaction model for each NMA (Additional file 1: eTable 6). The comparison-adjusted funnel plots for each NMA demonstrated no evidence of publication bias or small-study effects (Additional file 1: eFigure 2). Below we present all of the results for the primary and secondary outcomes (Table 2, Additional file 1: eTables 6-7). Results for meta-regression, sensitivity analyses, SUCRA curve values, and dose effects analyses can be found in Additional file 1: eTables 8-11.

**Table 2:** Network meta-analyses results comparing anti-VEGF agents

Treatment comparison	NMA estimate (95% CrI) (95% PrI)
**Proportion of patients experiencing vision gain (≥ 15 ETDRS letters)** 34 RCTs, 8809 patients, 12 treatments + placebo No inconsistency was observed in the overall NMA (chi-square = 1.79, *p* = 0.41) Between-study variance: 0.02 (0.00–0.14)	
Bevacizumab vs aflibercept	0.96 [(0.64–1.39) (0.54–1.62)]
Ranibizumab vs aflibercept	1.09 [(0.78–1.47) (0.65–1.76)]
Ranibizumab vs bevacizumab	1.14 [(0.9–1.43) (0.73–1.8)]
Brolucizumab vs aflibercept	1.2 [(0.85–1.71) (0.71–2.03)]
Brolucizumab vs bevacizumab	1.26 [(0.76–2.14) (0.67–2.44)]
Brolucizumab vs ranibizumab	1.11 [(0.71–1.8) (0.61–2.07)]
Conbercept vs aflibercept	0.19 [(0.06–0.65) (0.05–0.68)]^a^
Conbercept vs bevacizumab	0.2 [(0.06–0.69) (0.06–0.73)]^a^
Conbercept vs ranibizumab	0.17 [(0.05–0.59) (0.05–0.63)]^a^
Conbercept vs brolucizumab	0.15 [(0.05–0.56) (0.04–0.59)]^a^
**Proportion of patients experiencing vision loss of ≥ 15 ETDRS letters** 36 RCTs, 9081 patients, 13 treatments + placebo No inconsistency was observed in the overall NMA (chi-square = 0.25, *p* = 0.88) Between-study variance: 0.02 (0.00–0.13)	
Bevacizumab vs aflibercept	0.94 [(0.51–1.67) (0.47–1.81)]
Ranibizumab vs aflibercept	0.9 [(0.55–1.43) (0.5–1.59)]
Ranibizumab vs bevacizumab	0.96 [(0.69–1.35) (0.6–1.57)]
Brolucizumab vs aflibercept	0.96 [(0.57–1.63) (0.51–1.79)]
Brolucizumab vs bevacizumab	1.03 [(0.47–2.27) (0.44–2.43)]
Brolucizumab vs ranibizumab	1.08 [(0.53–2.19) (0.49–2.36)]
Conbercept vs aflibercept	0.24 [(0–4.29) (0–4.4)]
Conbercept vs bevacizumab	0.26 [(0–4.65) (0–4.67)]
Conbercept vs ranibizumab	0.27 [(0–4.67) (0–4.79)]
Conbercept vs brolucizumab	0.24 [(0–4.71) (0–4.85)]
**Mortality** 24 RCTs, 10 treatments + placebo, 8875 patients No inconsistency in the network (chi-squared = 0.69, *p*-value = 0.71) Between study variance: 0.01 (0.00-0.17)	
Bevacizumab vs aflibercept	0.58 [(0.15–1.98) (0.15–2.09)]
Ranibizumab vs aflibercept	0.59 [(0.17–1.8) (0.16–1.9)]
Ranibizumab vs bevacizumab	1.02 [(0.6–1.73) (0.54–1.94)]
Brolucizumab vs aflibercept	0.7 [(0.24–1.91) (0.23–2558)]
Brolucizumab vs bevacizumab	1.21 [(0.24–6.49) (0.23–2558)]
Brolucizumab vs ranibizumab	1.19 [(0.25–5.98) (0.24–2558)]
**Difference in mean change in BCVA** 26 RCTs, 10 treatments + placebo, 5916 patients No inconsistency in the network (chi-squared = 2.62, *p*-value = 0.27) Between-study variance: 6.29 (3.28–11.27)	
bevacizumab vs aflibercept	2.21 [(− 1.1 to 5.42) (− 3.96 to 8.22)]
ranibizumab vs aflibercept	1.09 [(− 1.53 to 3.7) (− 4.62 to 6.81)]
ranibizumab vs bevacizumab	− 1.11 [(− 3.07 to 0.92) (− 6.5 to 4.28)]
brolucizumab vs aflibercept	− 0.46 [(− 4.26 to 3.33) (− 6.84 to 5.81)]
brolucizumab vs bevacizumab	− 2.68 [(− 7.69 to 2.43) (− 9.72 to 4.54)]
brolucizumab vs ranibizumab	− 1.57 [(− 6.12 to 3.07) (− 8.34 to 5.32)]
conbercept vs aflibercept	− 15.17 [(− 23.8 to − 6.5) (− 25.35 to − 4.89)] ^a^
conbercept vs bevacizumab	− 17.35 [(− 25.84 to − 8.57) (− 27.14 to − 7.16)] ^a^
conbercept vs ranibizumab	− 16.23 [(− 24.57 to − 7.74) (− 25.97 to − 6.25)] ^a^
conbercept vs brolucizumab	− 14.68 [(− 24.01 to − 5.17) (− 25.48 to − 3.94)] ^a^
**Adverse events (AEs)** 15 RCTs, 8 treatments + placebo, 5785 patients No inconsistency in the network (chi-squared = 0.01, *p*-value = 0.93) Between-study variance: 0.01 (0.00–0.15)	
Bevacizumab vs aflibercept	1.11 [(0.53–2.1) (0.49–2.25)]
Ranibizumab vs aflibercept	1.23 [(0.76–1.93) (0.67–2.16)]
Ranibizumab vs bevacizumab	1.11 [(0.71–1.87) (0.63–2.12)]
Brolucizumab vs aflibercept	1.07 [(0.77–1.46) (0.67–1.69)]
Brolucizumab vs bevacizumab	0.97 [(0.48–2.14) (0.45–2.34)]
Brolucizumab vs ranibizumab	0.87 [(0.5–1.55) (0.46–1.72)]
Conbercept vs aflibercept	0.74 [(0.28–2) (0.26–2.09)]
Conbercept vs bevacizumab	0.67 [(0.22–2.15) (0.21–2.3)]
Conbercept vs ranibizumab	0.61 [(0.22–1.68) (0.21–1.77)]
Conbercept vs brolucizumab	0.69 [(0.25–1.96) (0.23–2.08)]
**Arterial thromboembolic events (ATE)** 15 RCTs, 8 treatments + placebo, 6365 patients No source of inconsistency in the network (no closed loops) Between-study variance: 0.03 (0.00–0.48)	
Bevacizumab vs aflibercept	1.13 [(0.31–4.32) (0.29–4.78)]
Ranibizumab vs aflibercept	1.81 [(0.61–5.86) (0.54–6.68)]
Ranibizumab vs bevacizumab	1.6 [(0.85–3.15) (0.7–3.85)]
Brolucizumab vs aflibercept	0.66 [(0.28–1.52) (0.24–1.82)]
Brolucizumab vs bevacizumab	0.58 [(0.12–2.61) (0.11–2.93)]
Brolucizumab vs ranibizumab	0.36 [(0.09–1.42) (0.08–1.57)]
Conbercept vs aflibercept	0.73 [(0.01–38.5) (0.01–39.9)]
Conbercept vs bevacizumab	0.66 [(0.01–31.63) (0.01–32.15)]
Conbercept vs ranibizumab	0.41 [(0.01–19.15) (0.01–20.03)]
Conbercept vs brolucizumab	1.1 [(0.02–62.85) (0.02–64.99)]

Note: The NMA estimates are odds ratios for all outcomes except the mean change in BCVA, which is reported as mean differences

^a^Statistically significant difference

#### Primary outcome: Vision gain

**Fig. 2:**
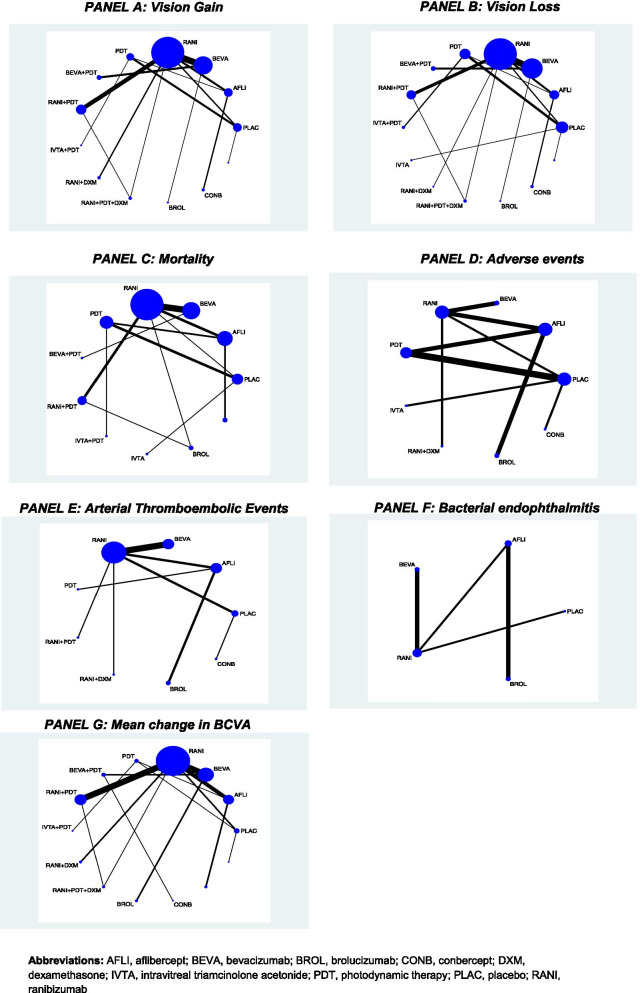
Network diagrams for primary and secondary outcomes. Illustration of networks for each network meta-analysis. Each treatment node indicates an intervention and is weighted according to the number of patients who received the particular intervention. Each edge (line connecting the nodes) is weighted according to the number of studies that directly compare the treatments it connects. Abbreviations: AFLI, aflibercept; BEVA, bevacizumab; BROL, brolucizumab; CONB, conbercept; DXM, dexamethasone; IVTA, intravitreal triamcinolone acetonide; PDT, photodynamic therapy; PLAC, placebo; RANI, ranibizumab

**Fig. 3:**
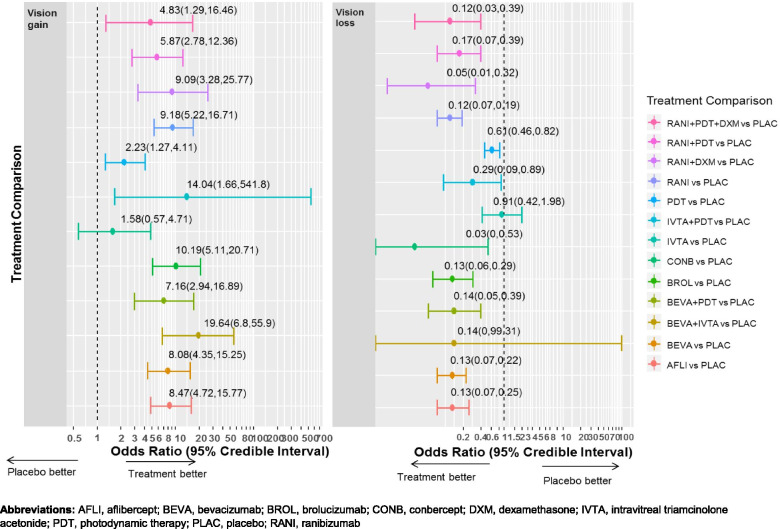
Forest plots of treatments versus placebo for vision gain and vision loss outcomes. Illustration of effect estimates for anti-VEGF agents compared to placebo for the outcomes of vision gain and vision loss. Abbreviations: AFLI, aflibercept; BEVA, bevacizumab; BROL, brolucizumab; CONB, conbercept; DXM, dexamethasone; IVTA, intravitreal triamcinolone acetonide; PDT, photodynamic therapy; PLAC, placebo; RANI, ranibizumab

For the number of patients who gained ≥15 ETDRS letters, NMA including 34 RCTs, 8,809 patients, and 12 treatments was conducted (Figs. 2 and 3). There were 78 treatment comparisons (Additional file 1: eTable 6) and the total event rate for the placebo group was 4.2%. For between agent comparisons, small differences were observed, with the largest for conbercept versus the following: broluczumab (OR 0.15, 95% CrI: 0.05–0.56), ranibizumab (OR 0.17, 95% CrI: 0.05–0.59), aflibercept (OR 0.19, 95% CrI: 0.06–0.65), and bevacizumab (OR 0.2, 95% CrI: 0.06–0.69) (Table 2).

CINeMA assessment

Comparisons between bevacizumab and ranibizumab, aflibercept and conbercept, bevacizumab and conbercept, brolucizumab and conbercept, and conbercept and ranibizumab received high confidence ratings. However, only bevacizumab and ranibizumab had direct evidence, and the remaining comparisons were based on indirect evidence alone. All other agent-to-agent comparisons received moderate to low confidence ratings (Additional file 1: eTable 12).

### Additional analyses

Meta-regression on study duration revealed no association between effect size and follow-up time (log-odds ratio estimate = − 0.02; 95% CrI: − 0.05 to 0.01). The sensitivity analysis including studies with > 100 patients (17 RCTs, 7 treatments, and 5953 patients) was consistent with the main analysis.

### Dose effects analysis

A dose effects analysis of the anti-VEGF agents alone, including 4 treatments and 9 different doses (ranibizumab [0.3mg, 0.5mg, 2mg], bevacizumab [1.25mg, 2.5mg], aflibercept [0.5mg, 2mg, 4mg], conbercept [0.5mg], and brolucizumab [3mg, 6mg]), was conducted for the outcome of vision gain. The results were consistent with the main analysis.

#### Primary outcome: Vision loss

For the proportion of patients who lost ≥ 15 ETDRS letters, NMA including 36 RCTs, 9,081 patients, and 13 treatments were conducted (Figs. 2 and 3). There were 91 treatment comparisons (Additional file 1: eTable 6) and the total event rate for the placebo group was 58%. Small differences were observed between the anti-VEGF agents, with the largest being for conbercept versus the following: aflibercept (OR 0.24, 95% CrI: (0-4.29), brolucizumab (OR 0.24, 95% CrI: 0–4.71), bevacizumab (OR 0.26, 95% CrI: 0–4.65), and ranibizumab (OR 0.27, 95% CrI: 0–4.67) (Table 2).

### CINeMA assessments

None of the agent-to-agent comparisons received a high confidence rating. Comparisons between bevacizumab and ranibizumab, as well as bevacizumab and conbercept received a moderate confidence rating, and all other agent-to-agent comparisons received a low confidence rating (Additional file 1: eTable 13).

### Additional analyses

Meta-regression on study duration revealed no association between effect size and follow-up time (estimate = 0.02, 95% CrI: 0.00 to 0.15). Sensitivity analysis including 18 RCTs with a sample size over 100 patients, 10 treatments, and 6214 patients was conducted and the results were consistent with the main analysis.

### Dose effects

A dose effects analysis including 4 treatments and 9 different doses (ranibizumab [0.3mg, 0.5mg, 2mg], bevacizumab [1.25mg, 2.5mg], aflibercept [0.5mg, 2mg, 4mg], triamcinolone acetonide [4mg], conbercept [0.5mg], and brolucizumab [3mg, 6mg]) was conducted for the outcome of vision loss. The results were consistent with the main analysis.

### Secondary outcomes

#### Mean change in BCVA

NMA including 26 RCTs, 6067 patients, and 10 treatments was conducted (Fig. 2). There were 91 treatment comparisons (Additional file 1: eTable 6). The largest differences between the anti-VEGF agents was for conbercept versus bevacizumab (MD -17.35, 95% CrI: − 25.84 to − 8.57), conbercept versus ranibizumab (MD − 16.23, 95% CrI: − 24.57 to − 7.74), conbercept versus aflibercept (MD − 15.17, 95% CrI: − 23.8 to − 6.5), and conbercept versus brolucizumab (MD − 14.68, 95% CrI: − 24.01 to − 5.17) (Table 2).

#### Legal blindness

NMA was not possible due to a lack of data for legal blindness. The total event rate for the placebo group was 48%. Across the 6 RCTs, one pairwise meta-analysis was possible that compared anti-VEGF agents (Additional file 1: eTable 7). Ranibizumab was found to have fewer cases of legal blindness when compared to bevacizumab (OR 0.00, 95% CrI: 0.00-0.03).

#### Vision-related function

Vision-related function on the National Eye Institute 25-Item Visual Function Questionnaire (NEI VFQ-25) composite score was not feasible to conduct NMA due to lack of data. However, across the 5 RCTs, pairwise meta-analysis was only possible for one comparison between anti-VEGF agents (Additional file 1: eTable 7), and patients treated with ranibizumab had similar scores for vision-related function when compared to those treated with aflibercept (MD 0.40, 95% CrI, − 1.59 to 2.40).

#### All-cause mortality

NMA including 24 RCTs, 8,875 patients, and 10 treatments was conducted (Fig. 2). There were 55 treatment comparisons with a total event rate for the placebo group of 2.0%. Small differences were observed between the anti-VEGF agents, with the largest being for bevacizumab versus aflibercept (OR 0.58, 95% CrI: 0.15–1.98) and ranibizumab versus aflibercept (OR 0.59, 95% CrI: 0.17–1.8) (Table 2).

#### Arterial thromboembolic events

NMA including 15 RCTs, 6365 patients, and 8 treatments was conducted (Fig. 2). There were 36 treatment comparisons with a total event rate for the placebo group of 2%. Small differences were observed between the anti-VEGF agents, with the largest being for ranibizumab versus aflibercept (OR 1.81, 95% CrI: 0.61–5.86) (Table 2).

#### Venous thromboembolic events

NMA was not possible due to a lack of data. Across the 12 RCTs, one pairwise meta-analysis was conducted (Additional file 1: eTable 7) and ranibizumab was associated with fewer venous thromboembolic events versus bevacizumab (OR 0.58, 95% CrI 0.01-1.91).

#### Bacterial endophthalmitis

NMA was not possible due to a lack of data. Across the 7 RCTs, two pairwise meta-analyses were possible (Additional file 1: eTable 7); brolucizumab was inferior to aflibercept (OR 5.70, 95% CrI: 0.65–187.90) and ranibizumab was superior to bevacizumab (OR 0.77, 95% CrI: 0.15-4.09).

#### Retinal detachment

NMA was not possible due to a lack of data. Across the 9 RCTs, two pairwise meta-analyses were possible between the anti-VEGF agents (Additional file 1: eTable 7); brolucizumab versus afliberept (OR 1.01, 95% CrI: 0.09-11.90) and ranibizumab versus bevacizumab (OR 0.93, 95% CrI: 0.09-9.78).

#### AEs overall

For AEs overall (i.e., not the specific AEs reported above), NMA including 15 RCTs, 5,785 patients, and 8 treatments was conducted (Fig. 2). There were 36 treatment comparisons with a total event rate for the placebo group of 53% (Additional file 1: eTable 6). There were small differences between the anti-VEGF agents, with the largest being for conbercept versus ranibizumab (OR 0.61, 95% CrI: 0.22–1.68) (Table 2).

#### Serious AEs

For the outcome of serious AEs, NMA was not possible due to a lack of data. Across the 8 studies, two pairwise meta-analyses were possible that compared the anti-VEGF agents (Additional file 1: eTable 7); brolucizumab versus aflibercept (OR 3.03, 95% CrI: 1.22–8.33) and ranibizumab versus bevacizumab (OR 0.86, 95% CrI: 0.59–1.22).

#### Withdrawals due to AEs

NMA was not possible due to a lack of data. The total event rate for the placebo group was 3%. Across the 11 RCTs, one pairwise meta-analysis was possible between the anti-VEGF agents (Additional file 1: eTable 7); ranibizumab versus bevacizumab (OR 1.20, 95% CrI: 0.47-3.14).

#### Rank-heat plot

The SUCRA curve demonstrated that the anti-VEGF agents were superior to all other comparators, yet none of the anti-VEGF agents were consistently superior to each other across all outcomes (Additional file 1: eFigure 3).

## Discussion

There were small differences between the anti-VEGF agents with the largest observed differences for conbercept compared to the other agents. Fewer patients treated with conbercept experienced vision gain when compared to other anti-VEGF agents. However, conbercept appeared most effective in terms of preventing vision loss and had fewer adverse events compared to other anti-VEGF agents. It should be noted that only one small trial (*n* = 123) comparing conbercept with sham (3 months of follow-up data) was included, and this should be taken into consideration when interpreting these results. Our dose-effects analysis for vision gain and vision loss demonstrated similar results. The rank-heat plot showed that the anti-VEGF agents are the most efficacious and safest when administered alone and compared to other agents. Furthermore, the anti-VEGF agents have similar effectiveness and safety profiles. However, outcome data were not available for all anti-VEGF agents and the 95% CrIs for the SUCRA curve values ranged widely (0–100%), suggesting that these results need to be interpreted alongside the effect sizes and measures of variance. Caution has been noted in interpreting SUCRA curve values, which may be unreliable [45]. The 95% CrIs around some of the effect sizes varied widely, suggesting that these results need to be interpreted with caution. For example, for the primary outcome vision gain, the result for IVTA+PDT vs. placebo varied widely (OR, 14.04; 95% CrI: 1.66 to 541.8).

Our results are consistent with guidance issued by the UK National Institute for Health and Care Excellence [46], and previous reviews. Fadda and colleagues conducted a systematic review and NMA of 5 RCTs, 4 anti-VEGF drugs, and placebo for AMD [8]. They found that anti-VEGF drugs were effective for decreasing vision loss of 15 ETDRS letters. Ranibizumab and bevacizumab were not different regarding effectiveness outcomes. Solomon and colleagues conducted a Cochrane review examining anti-VEGF (pegaptanib, ranibizumab, bevacizumab) for AMD [47]. They included 16 RCTs and found that the anti-VEGF agents increased proportion of patients with vision gain of 15 ETDRS letters or more, decreased proportion of patients with vision loss of 15 ETDRS letters, and improved vision (assessed at 20/200 or better) after one year of follow up versus controls. No differences were observed between bevacizumab and ranibizumab for visual acuity outcomes. However, our review was more comprehensive than these other reviews, including 67 to 74 more studies and 9 to 11 more treatments (Additional file 1: eTable 14). Moreover, we examined treatment combinations for neovascular AMD and a dose-effects analysis that can be used by patients and their clinicians when considering these agents.

### Limitations

There are limitations to the studies included in our review. Most of the included RCTs were assessed as having a high or unclear risk of bias due to random sequence generation and allocation concealment, which are the most important aspects that ensure validity of RCTs. The majority of the RCTs did not report mean age, comorbidities (e.g., diabetes, hypertension), or other confounding factors (e.g., lens status); thus, additional analyses were not possible for all outcomes. As well, the patients included in these RCTs might have more advanced illness; the incidence of legal blindness within one year of follow-up was 48% in the placebo group. Some of the RCTs included healthier individuals (without history of cardiovascular disease), which may have led to underestimating the harms (particularly mortality and adverse events) that might be associated with these agents in the real world. Furthermore, these trials were not sufficiently powered to detect harms from these agents, which suggests that our results are conservative. While we cannot conclude that these agents do not cause harm, it is reassuring that across all studies there was no significant increased risk of death or serious side effects. We were unable to conduct any sub-group analysis on severity of illness due to a lack of data. Finally, most RCTs were conducted within 12 months and future studies should look at longer-term use and sustainability of efficacy. Future studies should also consider using the recommended core outcomes set for macular degeneration [48], to ensure adequate data is available for meaningful comparison of treatments.

There were limitations in our systematic review process. We only included studies published in English due to time and resource limitations. Our protocol was developed for a therapeutic review looking at four retinal conditions [12]. The current systematic review built off of this work, but deviates from the protocol in terms of focusing only on one retinal condition (nAMD), and the inclusion of newer anti-VEGF drugs. We planned to include increased intra-ocular pressure as an outcome; however, we excluded this because the included studies did not specify the extent to which intraocular pressure changed so the results were not clinically relevant. Our outcome selection was informed by clinical experts and patient group input, due to a lack of a core outcome set when the protocol was developed. A core outcome set was published the following year [48], but we chose to remain consistent with the outcomes in our pre-established protocol. In particular, the value of patient-reported outcome measures (PROM) was highlighted, and while we included the NEI VFQ-25, this PROM was not recommended by the working group [48]. In addition, we did not explore the impact of different treatment regimens for anti-VEGF agents in our dose effects analysis and recommend that this be explored in future studies. NMAs were not feasible for many of the safety outcomes due to a dearth of data. We did not include observational studies, which may have provided a more complete safety profile for these agents. This is especially important because many of the adverse events examined here are rare and long-term observational data are required to fully examine these harms.

## Conclusions

Anti-VEGF agents are superior to other medications on the market, especially when administered alone. The anti-VEGF agents have similar effectiveness and safety profiles. These results can be used by decision-makers, such as patients and healthcare providers regarding the use of anti-VEGF agents.

## Supplementary Information

**Additional file 1: **Supplementary Online Content. The appendix include all supplemental data and information. **eAppendix 1.** Systematic Review Protocol as Registered in PROSPERO (CRD42015022041). **eAppendix 2.** PRISMA NMA Checklist of Items to Include When Reporting a Systematic Review Involving a Network Meta-analysis. **eAppendix 3.** Outcome Definitions. **eAppendix 4.** MEDLINE/EMBASE Literature Search Strategy. **eTable 1.** Recommended Dosage of Anti-VEGF Agents for Treatment of Wet AMD. **eTable 2.** Study Characteristics. **eTable 3.** Patient Characteristics. **eFigure 1.** Aggregate Risk of Bias Figure. **eTable 4.** Cochrane Risk of Bias Results for Individual Studies. **eTable 5.** Transitivity Assessment for all NMA Outcomes. **eTable 6.** All Network Meta-Analyses Results. **eFigure 2.** Comparison-adjusted Funnel Plots. Vision Gain. Vision Loss. Mean Change in Best-corrected Visual Acuity. Mortality. Arterial Thromboembolic Events. Adverse Events. **eTable 7.** All Pairwise Meta-Analysis Results. **eTable 8.** Sensitivity Network Meta-Analysis results. Outcome: VISION GAIN, Outcome: VISION LOSS, **eTable 9.** Surface Under the Cumulative Ranking Curve (SUCRA) Values for the Overall NMA and Subgroup Analyses for Vision Gain and Vision Loss. **eTable 10.** Surface Under the Cumulative Ranking Curve (SUCRA) Results for all Other Outcomes. **eTable 11.** Dose effects network meta-analysis (NMA) results. **eTable 12.** Confidence in Network Meta-Analysis (CINeMA) assessment for the outcome of vision gain. **eTable 13.** Confidence in Network Meta-Analysis (CINeMA) assessment for the outcome of vision loss. **eFigure 3.** Rank Heat Plot. **eTable 14.** Comparison to Previous Systematic Reviews.

## Data Availability

The datasets used and/or analysed during the current study are available from the corresponding author on reasonable request.

## Abbreviations

AEs: Adverse events
AMD: Age-related macular degeneration
Anti-VEGF: Anti-vascular endothelial growth factor
ATE: Arterial thromboembolic events
BCVA: Best-corrected visual acuity
BE: Bacterial endophthalmitis
CADTH: Canadian Agency for Drugs and Technologies in Health
CrI: Credible interval
DXM: Dexamethasone implant
ETDRS: Early Treatment Diabetic Retinopathy Study
IVTA: Intravitreal triamcinolone acetonide
MCMC: Markov Chain Monte Carlo
MD: Mean differences
NEI VFQ-25: National Eye Institute 25-Item Visual Function Questionnaire
nAMD: Neovascular age-related macular degeneration
NMA: Network meta-analysis
OR: Odds ratio
PRISMA-P: Preferred Reporting Items for Systematic reviews and Meta-analyses for Protocols
PDT: Photodynamic therapy
PrI: Predictive interval
PROM: Patient-reported outcome measures
RCT: Randomized controlled trial
SUCRA: Surface Under The Cumulative Ranking
VTE: Venous thromboembolic events
