# Supplementary material for: Anti-vascular endothelial growth factor therapy for age-related macular degeneration: a systematic review and network meta-analysis
Source: Syst Rev. 2021 Dec 20;10:315. doi: 10.1186/s13643-021-01864-6 (PMC8690960; doi:10.1186/s13643-021-01864-6)
Supplement: Supplementary file 1 — Additional file 1: Supplementary Online Content. The appendix include all supplemental data and information. eAppendix 1. Systematic Review Protocol as Registered in PROSPERO (CRD42015022041). eAppendix 2. PRISMA NMA Checklist of Items to Include When Reporting a Systematic Review Involving a Network Meta-analysis. eAppendix 3. Outcome Definitions. eAppendix 4. MEDLINE/EMBASE Literature Search Strategy. eTable 1. Recommended Dosage of Anti-VEGF Agents for Treatment of Wet AMD. eTable 2. Study Characteristics. eTable 3. Patient Characteristics. eFigure 1. Aggregate Risk of Bias Figure. eTable 4. Cochrane Risk of Bias Results for Individual Studies. eTable 5. Transitivity Assessment for all NMA Outcomes. eTable 6. All Network Meta-Analyses Results. eFigure 2. Comparison-adjusted Funnel Plots. Vision Gain. Vision Loss. Mean Change in Best-corrected Visual Acuity. Mortality. Arterial Thromboembolic Events. Adverse Events. eTable 7. All Pairwise Meta-Analysis Results. eTable 8. Sensitivity Network Meta-Analysis results. Outcome: VISION GAIN, Outcome: VISION LOSS, eTable 9. Surface Under the Cumulative Ranking Curve (SUCRA) Values for the Overall NMA and Subgroup Analyses for Vision Gain and Vision Loss. eTable 10. Surface Under the Cumulative Ranking Curve (SUCRA) Results for all Other Outcomes. eTable 11. Dose effects network meta-analysis (NMA) results. eTable 12. Confidence in Network Meta-Analysis (CINeMA) assessment for the outcome of vision gain. eTable 13. Confidence in Network Meta-Analysis (CINeMA) assessment for the outcome of vision loss. eFigure 3. Rank Heat Plot. eTable 14. Comparison to Previous Systematic Reviews. [file 13643_2021_1864_MOESM1_ESM.docx]

**Supplementary Online Content**

[eAppendix 1: Systematic Review Protocol as Registered in PROSPERO (CRD42015022041) 3](#_Toc85186208)

[eAppendix 2: PRISMA NMA Checklist of Items to Include When Reporting a Systematic Review Involving a Network Meta-analysis 7](#_Toc85186209)

[eAppendix 3: Outcome Definitions 11](#_Toc85186210)

[eAppendix 4: MEDLINE/EMBASE Literature Search Strategy 12](#_Toc85186211)

[eTable 1: Recommended Dosage of Anti-VEGF Agents for Treatment of Wet AMD 17](#_Toc85186212)

[eTable 2: Study Characteristics 18](#_Toc85186213)

[eTable 3: Patient Characteristics 26](#_Toc85186214)

[eFigure 1: Aggregate Risk of Bias Figure 30](#_Toc85186215)

[eTable 4: Cochrane Risk of Bias Results for Individual Studies 31](#_Toc85186216)

[eTable 5: Transitivity Assessment for all NMA Outcomes 34](#_Toc85186217)

[eTable 6: All Network Meta-Analyses Results 39](#_Toc85186218)

[eFigure 2: Comparison-adjusted Funnel Plots 49](#_Toc85186219)

[Vision Gain 49](#_Toc85186220)

[Vision Loss 49](#_Toc85186221)

[Mean Change in Best-corrected Visual Acuity 50](#_Toc85186222)

[Mortality 50](#_Toc85186223)

[Arterial Thromboembolic Events 51](#_Toc85186224)

[Adverse Events 51](#_Toc85186225)

[eTable 7: All Pairwise Meta-Analysis Results 53](#_Toc85186226)

[eTable 8: Sensitivity Network Meta-Analysis results 57](#_Toc85186227)

[**Outcome: VISION GAIN** 57](#_Toc85186228)

[**Outcome: VISION LOSS** 59](#_Toc85186229)

[eTable 9: Surface Under the Cumulative Ranking Curve (SUCRA) Values for the Overall NMA and Subgroup Analyses for Vision Gain and Vision Loss 61](#_Toc85186230)

[eTable 10: Surface Under the Cumulative Ranking Curve (SUCRA) Results for all Other Outcomes 62](#_Toc85186231)

[eTable 11: Dose effects network meta-analysis (NMA) results 64](#_Toc85186232)

[eTable 12: Confidence in Network Meta-Analysis (CINeMA) assessment for the outcome of vision gain 69](#_Toc85186233)

[eTable 13: Confidence in Network Meta-Analysis (CINeMA) assessment for the outcome of vision loss 73](#_Toc85186234)

[eFigure 3: Rank Heat Plot 78](#_Toc85186235)

[eTable 14: Comparison to Previous Systematic Reviews 80](#_Toc85186236)

[References 81](#_Toc85186237)

# eAppendix 1: Systematic Review Protocol as Registered in PROSPERO (CRD42015022041)

| \| \| **Comparative efficacy and safety of three different anti-vascular endothelial growth factor (VEGF) drugs for retinal conditions: a systematic review** \| \| --- \| \| *Andrea C. Tricco, Sharon Straus, Sonia Thomas, Erin Lillie, Taehoon Lee, Jaimie Adams, Trevor Richter, Ghayath Janoudi* \| \| **Citation**  Andrea C. Tricco, Sharon Straus, Sonia Thomas, Erin Lillie, Taehoon Lee, Jaimie Adams, Trevor Richter, Ghayath Janoudi. Comparative efficacy and safety of three different anti-vascular endothelial growth factor (VEGF) drugs for retinal conditions: a systematic review. PROSPERO 2015:CRD42015022041 Available from <http://www.crd.york.ac.uk/PROSPERO/display_record.asp?ID=CRD42015022041> \| \| **Review question(s)**  What is the comparative efficacy and safety of anti-VEGF drugs amongst adults with the following conditions?  1) Neovascular (wet) age-related macular degeneration (AMD)  2) Visual impairment due to diabetic macular oedema (DME)  3) Macular oedema due to retinal vein occlusion (RVO)  4) Choroidal neovascularization (CNV) secondary to pathologic myopia (PM)  **Searches**  The literature search will be performed by an experienced information specialist using a peer-reviewed search strategy.  Published literature will be identified by searching MEDLINE, Embase, and the Cochrane Central Register of Controlled Trials from inception onwards.  Difficult to locate and unpublished (sometimes referred to as “grey”) literature will be identified by searching www.clinicaltrials.gov. These searches will be supplemented by reviewing the reference lists of included studies and contacting the drug manufacturers.  **Types of study to be included**  Parallel or cluster randomized controlled trials will be eligible for inclusion  **Condition or domain being studied**  Diabetic macular edema (DME), wet age-related macular degeneration (AMD), macular edema due to retinal vein occlusion (RVO), choroidal neovascularization (CNV) due to pathologic myopia.  **Participants/ population**  The study population will include adults with any of the following conditions: wet AMD, DME, RVO and/or CNV secondary to pathologic myopia.  **Intervention(s), exposure(s)**  Bevacizumab, Aflibercept, Ranibizumab  **Comparator(s)/ control**  Bevacizumab, Aflibercept, Ranibizumab, Pegaptanib, photodynamic therapy verteporfin, corticosteroids (only Triamcinolone Acetonide intravitreal injection, Dexamethasone implant, Fluocinolone Acetonide implant), laser photocoagulation, placebo/no treatment  **Context**  All medical contexts will be included regardless of region/country where the trial(s) were conducted.  **Outcome(s)**  Primary outcomes  Efficacy outcomes:  1. Change (gain or loss) in best corrected visual acuity (BCVA) of =15 EDTRS letters  2. Change from baseline in BCVA  3. Blindness (legal)  4. Vision-related function  Secondary outcomes  Harms outcomes:  1. Adverse events  2. Serious adverse event  3. Withdrawals due to adverse events  4. Mortality  5. Harms of special interest:  • Arterial/venous thromboembolic events  • Bacterial endophthalmitis  • Increased intra-ocular pressure  • Retinal detachment  **Data extraction, (selection and coding)**  All titles and abstracts (level 1 screening) will be screened in duplicate, and any discrepancies will be resolved by a third reviewer. This will be repeated for the level 2 screening (screening of full-text papers). A standardized data abstraction form will be used to abstract data on study characteristics (e.g., study design, year of conduct, sample size), patient characteristics (e.g., mean age and standard deviation, comorbidities), and the definitions of outcomes (e.g., quality of life scale used).  **Risk of bias (quality) assessment**  Risk of bias in the included studies will be appraised using the Cochrane risk of bias tool.  **Strategy for data synthesis**  A pair-wise random-effects meta-analysis will be attempted for each treatment indication. We may consider conducting a random-effects network meta-analysis within each indication, should a connected network with a sufficient number of studies become available.  **Analysis of subgroups or subsets**  Important heterogeneity (e.g., an I-squared statistic >75%) will be explored using meta-regression analysis and sub-group analysis.  **Dissemination plans**  The summary of results will be used by CADTH to develop a recommendation report for policy makers. One or more posters/oral presentation will be prepared as well as and a manuscript for publication in an open-access, peer-reviewed journal.  **Contact details for further information**  Dr Tricco  30 Bond Street, Toronto, Ontario, M5B 1W8  triccoa@smh.ca  **Organisational affiliation of the review**  Knowledge Translation Program at Li Ka Shing Knowledge Institute, St. Michael’s Hospital  http://www.stmichaelshospital.com/knowledgeinstitute/index.php  **Review team**  Dr Andrea C. Tricco, Knowledge Translation Program at Li Ka Shing Knowledge Institute, St. Michael’s Hospital Dr Sharon Straus, Knowledge Translation Program at Li Ka Shing Knowledge Institute, St. Michael’s Hospital Ms Sonia Thomas, Knowledge Translation Program at Li Ka Shing Knowledge Institute, St. Michael’s Hospital Ms Erin Lillie, Knowledge Translation Program at Li Ka Shing Knowledge Institute, St. Michael’s Hospital Dr Taehoon Lee, Knowledge Translation Program at Li Ka Shing Knowledge Institute, St. Michael’s Hospital Ms Jaimie Adams, Knowledge Translation Program at Li Ka Shing Knowledge Institute, St. Michael’s Hospital Dr Trevor Richter, Canadian Agency for Drugs and Technologies in Health (CADTH) Dr Ghayath Janoudi, Canadian Agency for Drugs and Technologies in Health (CADTH)  **Collaborators**  Ms Becky Skidmore, Li Ka Shing Knowledge Institute, St. Michael’s Hospital Ms Alissa Epworth, Li Ka Shing Knowledge Institute, St. Michael’s Hospital  **Details of any existing review of the same topic by the same authors**  Not applicable.  **Anticipated or actual start date**  01 May 2015  **Anticipated completion date**  09 February 2016  **Funding sources/sponsors**  We received funding for this systematic review from the Canadian Institutes of Health Research (CIHR) Drug Safety and Effectiveness Network (DSEN). ACT is funded by a CIHR/DSEN New Investigator Award in Knowledge Synthesis and SES is funded by a Tier 1 Canada Research Chair in Knowledge Translation.  **Conflicts of interest**  None known  **Other registration details**  Not applicable.  **Language**  English  **Country**  Canada  **Subject index terms status**  Subject indexing assigned by CRD  **Subject index terms**  Diabetic Retinopathy; Humans; Retinal Diseases; Vascular Endothelial Growth Factor A  **Reference and/or URL for protocol**  Anti-vascular endothelial growth factor drugs for retinal conditions: a therapeutic review - project protocol. Ottawa: CADTH; 2015 Jul. (CADTH Therapeutic Review vol.3, no.2a).  <https://www.cadth.ca/sites/default/files/pdf/TR0009_Anti-VEGF_Protocol_e.pdf>  **Stage of review**  Completed published and being updated  **Date of registration in PROSPERO**  01 June 2015  **Date of publication of this revision**  20 January 2017  **Details of final report/publication(s)**  Anti–vascular endothelial growth factor drugs for the treatment of retinal conditions. Ottawa: CADTH; 2016 Apr. (CADTH therapeutic review; vol. 3, no. 2b) <https://www.cadth.ca/sites/default/files/pdf/TR0009_Anti-VEGF_TR_Science_Report.pdf>  **DOI**  10.15124/CRD42015022041   \| **Stage of review at time of this submission** \| **Started** \|  \| **Completed** \| \| --- \| --- \| --- \| --- \| \| Preliminary searches \| Yes \|  \| Yes \| \| Piloting of the study selection process \| Yes \|  \| Yes \| \| Formal screening of search results against eligibility criteria \| Yes \|  \| Yes \| \| Data extraction \| Yes \|  \| Yes \| \| Risk of bias (quality) assessment \| Yes \|  \| Yes \| \| Data analysis \| Yes \|  \| Yes \| \| \| \| --- \| --- \| --- \| --- \| --- \| --- \| --- \| --- \| --- \| --- \| --- \| --- \| --- \| --- \| --- \| --- \| --- \| --- \| --- \| --- \| --- \| --- \| --- \| --- \| --- \| --- \| --- \| --- \| --- \| --- \| --- \| --- \| --- \| |
| --- | --- | --- | --- | --- | --- | --- | --- | --- | --- | --- | --- | --- | --- | --- | --- | --- | --- | --- | --- | --- | --- | --- | --- | --- | --- | --- | --- | --- | --- | --- | --- | --- | --- |

# eAppendix 2: PRISMA NMA Checklist of Items to Include When Reporting a Systematic Review Involving a Network Meta-analysis

| **Section/Topic** | **Item #** | **Checklist Item*** | **Reported on Page #** |
| --- | --- | --- | --- |
| **TITLE** |  |  |  |
| Title | 1 | Identify the report as a systematic review *incorporating a network meta-analysis (or related form of meta-analysis).* | 1 |
|  |  |  |  |
| **ABSTRACT** |  |  |  |
| Structured summary | 2 | Provide a structured summary including, as applicable:  **Background:** main objectives  **Methods:** data sources; study eligibility criteria, participants, and interventions; study appraisal; and *synthesis methods, such as network meta-analysis.*  **Results:** number of studies and participants identified; summary estimates with corresponding confidence/credible intervals; *treatment rankings may also be discussed. Authors may choose to summarize pairwise comparisons against a chosen treatment included in their analyses for brevity.*  **Discussion/Conclusions:** limitations; conclusions and implications of findings.  **Other:** primary source of funding; systematic review registration number with registry name. | 4-5 |
|  |  |  |  |
| **INTRODUCTION** |  |  |  |
| Rationale | 3 | Describe the rationale for the review in the context of what is already known*, including mention of why a network meta-analysis has been conducted.* | 6-7 |
| Objectives | 4 | Provide an explicit statement of questions being addressed, with reference to participants, interventions, comparisons, outcomes, and study design (PICOS). | 6-7 |
|  |  |  |  |
| **METHODS** |  |  |  |
| Protocol and registration | 5 | Indicate whether a review protocol exists and if and where it can be accessed (e.g., Web address); and, if available, provide registration information, including registration number. | 7 |
| Eligibility criteria | 6 | Specify study characteristics (e.g., PICOS, length of follow-up) and report characteristics (e.g., years considered, language, publication status) used as criteria for eligibility, giving rationale. *Clearly describe eligible treatments included in the treatment network, and note whether any have been clustered or merged into the same node (with justification).* | 7-8 |
| Information sources | 7 | Describe all information sources (e.g., databases with dates of coverage, contact with study authors to identify additional studies) in the search and date last searched. | 8 |
| Search | 8 | Present full electronic search strategy for at least one database, including any limits used, such that it could be repeated. | 8-9, eAppendix 4 |
| Study selection | 9 | State the process for selecting studies (i.e., screening, eligibility, included in systematic review, and, if applicable, included in the meta-analysis). | 9 |
| Data collection process | 10 | Describe method of data extraction from reports (e.g., piloted forms, independently, in duplicate) and any processes for obtaining and confirming data from investigators. | 9 |
| Data items | 11 | List and define all variables for which data were sought (e.g., PICOS, funding sources) and any assumptions and simplifications made. | 9 |
| Geometry of the network | **S1** | Describe methods used to explore the geometry of the treatment network under study and potential biases related to it. This should include how the evidence base has been graphically summarized for presentation, and what characteristics were compiled and used to describe the evidence base to readers. | 10-12 |
| Risk of bias within individual studies | 12 | Describe methods used for assessing risk of bias of individual studies (including specification of whether this was done at the study or outcome level), and how this information is to be used in any data synthesis. | 9-10 |
| Summary measures | 13 | State the principal summary measures (e.g., risk ratio, difference in means). *Also describe the use of additional summary measures assessed, such as treatment rankings and surface under the cumulative ranking curve (SUCRA) values, as well as modified approaches used to present summary findings from meta-analyses.* | 10-12 |
| Planned methods of analysis | 14 | Describe the methods of handling data and combining results of studies for each network meta-analysis. This should include, but not be limited to:   - *Handling of multi-arm trials;* - *Selection of variance structure;* - *Selection of prior distributions in Bayesian analyses; and* - *Assessment of model fit.* | 10-12 |
| Assessment of Inconsistency | **S2** | Describe the statistical methods used to evaluate the agreement of direct and indirect evidence in the treatment network(s) studied. Describe efforts taken to address its presence when found. | 10-12 |
| Risk of bias across studies | 15 | Specify any assessment of risk of bias that may affect the cumulative evidence (e.g., publication bias, selective reporting within studies). | 10-12 |
| Additional analyses | 16 | Describe methods of additional analyses if done, indicating which were pre-specified. This may include, but not be limited to, the following:   - Sensitivity or subgroup analyses; - Meta-regression analyses; - *Alternative formulations of the treatment network; and* - *Use of alternative prior distributions for Bayesian analyses (if applicable).* | 10-12 |
|  |  |  |  |
| **RESULTS†** |  |  |  |
| Study selection | 17 | Give numbers of studies screened, assessed for eligibility, and included in the review, with reasons for exclusions at each stage, ideally with a flow diagram. | 12, Fig. 1 |
| **Presentation of network structure** | **S3** | Provide a network graph of the included studies to enable visualization of the geometry of the treatment network. | Fig. 2 |
| **Summary of network geometry** | **S4** | Provide a brief overview of characteristics of the treatment network. This may include commentary on the abundance of trials and randomized patients for the different interventions and pairwise comparisons in the network, gaps of evidence in the treatment network, and potential biases reflected by the network structure. | 12-13 |
| Study characteristics | 18 | For each study, present characteristics for which data were extracted (e.g., study size, PICOS, follow-up period) and provide the citations. | 12-13, eTable 2 |
| Risk of bias within studies | 19 | Present data on risk of bias of each study and, if available, any outcome level assessment. | eTable 4 |
| Results of individual studies | 20 | For all outcomes considered (benefits or harms), present, for each study: 1) simple summary data for each intervention group, and 2) effect estimates and confidence intervals. *Modified approaches may be needed to deal with information from larger networks.* | NA |
| Synthesis of results | 21 | Present results of each meta-analysis done, including confidence/credible intervals. *In larger networks, authors may focus on comparisons versus a particular comparator (e.g. placebo or standard care), with full findings presented in an appendix. League tables and forest plots may be considered to summarize pairwise comparisons.* If additional summary measures were explored (such as treatment rankings), these should also be presented. | 14-18, eTable 6-10 |
| **Exploration for inconsistency** | **S5** | Describe results from investigations of inconsistency. This may include such information as measures of model fit to compare consistency and inconsistency models, *P* values from statistical tests, or summary of inconsistency estimates from different parts of the treatment network. | 13-14, eTable 5-6 |
| Risk of bias across studies | 22 | Present results of any assessment of risk of bias across studies for the evidence base being studied. | 13, eFigure 1 |
| Results of additional analyses | 23 | Give results of additional analyses, if done (e.g., sensitivity or subgroup analyses, meta-regression analyses*, alternative network geometries studied, alternative choice of prior distributions for Bayesian analyses,* and so forth). | 14-18, eTables 8, 9 and 11 |
|  |  |  |  |
| **DISCUSSION** |  |  |  |
| Summary of evidence | 24 | Summarize the main findings, including the strength of evidence for each main outcome; consider their relevance to key groups (e.g., healthcare providers, users, and policy-makers). | 18- 20 |
| Limitations | 25 | Discuss limitations at study and outcome level (e.g., risk of bias), and at review level (e.g., incomplete retrieval of identified research, reporting bias). *Comment on the validity of the assumptions, such as transitivity and consistency. Comment on any concerns regarding network geometry (e.g., avoidance of certain comparisons).* | 20-21 |
| Conclusions | 26 | Provide a general interpretation of the results in the context of other evidence, and implications for future research. | 21 |
|  |  |  |  |
| **FUNDING** |  |  |  |
| Funding | 27 | Describe sources of funding for the systematic review and other support (e.g., supply of data); role of funders for the systematic review. This should also include information regarding whether funding has been received from manufacturers of treatments in the network and/or whether some of the authors are content experts with professional conflicts of interest that could affect use of treatments in the network. | 23 |

# eAppendix 3: Outcome Definitions

| **Outcome** | **Definition** |
| --- | --- |
| Vision gain | The proportion of patients experiencing a gain in best corrected visual acuity (BCVA) of 15 Early Treatment Diabetic Retinopathy Study (ETDRS) letters or more (3 lines or more) |
| Vision loss | The proportion of patients experiencing a loss in best corrected visual acuity (BCVA) of 15 Early Treatment Diabetic Retinopathy Study (ETDRS) letters or more (3 lines or more) |
| Mean change in BCVA | The mean change in BCVA scores (ETDRS letters) from baseline to follow-up |
| Legal blindness | Legal blindness is defined as worse than or equal to 20/200 with best correction in the better eye or a visual field extent of less than 20 degrees in diameter |
| Vision related function | The mean change in the composite vision related function score as measured by the validated National Eye Institute 25-Item Visual Function Questionnaire (NEI VFQ-25) from baseline to follow-up |
| Adverse events | The total/overall reported number of adverse events or harms |
| Serious adverse events | The total/overall reported number of serious adverse events/harms |
| Withdrawals due to adverse events | The number of patients reported as withdrawn from the study due to adverse events |
| Mortality | The number of patients reported for death regardless of causality |
| Arterial thromboembolic events | Includes myocardial infarction (MI), unstable angina, ischemic stroke, transient ischemic attack (TIA) or any other arterial thromboembolic event reported |
| Venous thromboembolic events | Includes deep vein thrombosis (DVT), pulmonary embolism (PE), cavernous sinus thrombosis, central or branch retinal vein occlusion, or any venous thromboembolic event reported |
| Bacterial endophthalmitis | An inflammatory reaction of the intraocular fluids or tissues caused by microbial organisms |
| Retinal detachment | Retinal detachment occurs when the retina pulls away from the layer of blood vessels that provides it with oxygen and nourishment |

# eAppendix 4: MEDLINE/EMBASE Literature Search Strategy

Database: Ovid MEDLINE(R) In-Process & Other Non-Indexed Citations and Ovid MEDLINE(R) <1946 to Present>, Embase <1974 to 2015 Nov 13>

Search Strategy:

--------------------------------------------------------------------------------

1 Retinal Degeneration/

2 limit 1 to yr="1973-2009"

3 Macular Degeneration/

4 Wet Macular Degeneration/

5 ((exudative or neovascular or wet) adj3 ((macula* adj2 degeneration) or (macula* adj2 deterioration) or maculopath* or (macula* adj2 dystroph*) or (macula* adj2 atroph*))).tw,kw.

6 ((exudative or neovascular or wet) adj2 (AMD or ARMD)).tw,kw.

7 (wAMD or wARMD).tw,kw.

8 Diabetic Retinopathy/

9 ((diabet* or DM) adj3 (maculopath* or retinopath*)).tw,kw.

10 (PDR or DME or DMO).tw,kw.

11 Macular Edema/

12 ((macula* or retina*) adj3 (edema$1 or oedema$1)).tw,kw.

13 (Irvine-Gass adj3 (edema$1 or oedema$1 or syndrome$1)).tw,kw.

14 (cystoid macula* adj dystroph*).tw,kw.

15 Retinal Vein Occlusion/

16 (retinal vein adj3 (occlu* or obstruct* or clos* or stricture* or steno* or block* or embolism*)).tw,kw.

17 (BRVO or CRVO).tw,kw.

18 Choroidal Neovascularization/

19 ((choroid* or subretinal or sub-retinal) adj1 neovasculari#ation*).tw,kw.

20 CNV.tw,kw.

21 or/2-20

22 Vascular Endothelial Growth Factor A/ai or "Receptors, Vascular Endothelial Growth Factor"/ai

23 (anti adj2 VEGF$1).tw,kw.

24 (antiVEGF$1 or VEGF inhibitor* or VEGF antagonist*).tw,kw.

25 (antivascular endothelial growth factor$1 or anti-vascular endothelial growth factor$1).tw,kw.

26 Antibodies, Monoclonal, Humanized/

27 (monoclonal antibod* and humani#ed).tw,kw.

28 (antibod* adj2 humani#ed).tw,kw.

29 Angiogenesis Inhibitors/

30 (angiogen* adj3 (inhibitor* or antagonist*)).tw,kw.

31 (anti-angiogen* or antiangiogen*).tw,kw.

32 aflibercept.tw,kw.

33 ("AVE 0005" or AVE0005 or "AVE 005" or AVE005 or "Bay 86-5321" or "Bay86-5321" or Eylea or "UNII-15C2VL427D" or Zaltrap or ZIV-aflibercept).tw,kw.

34 ((vasculotropin or vascular endothelial growth factor or VEGF) adj trap*).tw,kw.

35 aflibercept.rn,nm.

36 Bevacizumab.tw,kw.

37 (Altuzan or Avastin or "nsc 704865" or nsc704865 or "rhuMAb-VEGF" or "UNII-2S9ZZM9Q9V").tw,kw.

38 IVB injection$1.tw,kw.

39 Bevacizumab.rn,nm.

40 Pegaptanib.tw,kw.

41 ("EYE 001" or EYE001 or Macugen or "NX 1838" or NX1838 or "UNII-3HP012Q0FH").tw,kw.

42 Pegaptanib.rn,nm.

43 Ranibizumab.tw,kw.

44 (Lucentis or "rhuFab V2" or "UNII-ZL1R02VT79").tw,kw.

45 IVR injection$1.tw,kw.

46 Ranibizumab.rn,nm.

47 or/22-46

48 21 and 47

49 exp Photochemotherapy/

50 Photosensitizing Agents/

51 (photochemo* or photo-chemo* or photodynamic* or photo-dynamic* or photosensiti* or photo-sensiti*).tw,kw.

52 PDT.tw,kw.

53 or/49-52

54 verteporfin.tw,kw.

55 (verteporphin or "BPD-MA" or "CL 318,952" or "CL 318952" or "UNII-0X9PA28K43" or Visudyne).tw,kw.

56 verteporfin.rn,nm.

57 or/54-56

58 53 and 57

59 (PDTV or "PDT-V" or VPDT or "V-PDT").tw,kw.

60 58 or 59

61 21 and 60

62 exp Triamcinolone/

63 ((Triamcinol* adj acet*) or (Triamcincol* adj acet*) or (Triamsinol* adj acet*) or Acetospan or Adcortyl or AllerNaze or Aristocort or Aristoderm or Aristogel or Aristospan or Asmacort or Azmacort or "BRN 0060069" or "CCRIS 5231" or Cinonide or Clinacort or "Coupe-A" or "EINECS 200-948-7" or Flutex or Flutone or FX006 or Kenacort* or Kenalog* or Kenalone or Kenlog or Nasacort or "NSC 21916" or Omcilon or Oracort or Oralone or Polcortolon or Rineton or Solodelf or Tramacin or Triacet$2 or Triacort or Triamcot or Triam-Forte or Triam-Injekt or Triamonide or Trianex or Triatex or Tricinolon or Tricort* or Triderm or Triesence or Triesense or Tri-nasal or Tristoject or Trivaris or Trymex or "UNII-F446C597KA" or Volon).tw,kw.

64 triamcinolone.rn,nm.

65 triamcinolone acetonide.rn,nm.

66 Glucocorticoids/

67 (glucocorticoid* or glucorticoid*).tw,kw.

68 (anecortave or "AL 3789" or AL3789 or "EINECS 231-812-5" or "NSC 15475" or "NSC 24345" or Retaane or "UNII-Y0PC411K4T").tw,kw.

69 anecortave acetate.rn,nm.

70 exp Fluocinolone Acetonide/

71 ((Fluocinolon* adj Acet*) or Alvadermo or Capex or Co-Fluocin or Cortiespec or "EINECS 200-668-5" or Flucinar or Fluocid or Flucort or Fluocet or Fluonid or Fluotrex or (Fluortriamcinolon* adj Acet*) or Flurosyn or Flusolgen or Gelidina or Iluvien or Jellin or Jellisoft or Percutina or Radiocin or Retisert or Sinalar or Synalar or Synamol or Synandone or Synandrone or Synamol or Synemol or Synsac or Tefunote or "UNII-0CD5FD6S2M").tw,kw.

72 fluocinolone acetonide.rn,nm.

73 Pregnadienediols/

74 (dihydroxypregnadiene* or di-hydroxypregnadiene* or pregnadienediol*).tw,kw.

75 exp Dexamethasone/

76 (Dexamethasone or Decaject* or Decameth or Dexasone or Dexpak or Hexadecadrol or Hexadrol or Maxidex or Millicorten or Oradexon or Ozurdex).tw,kw.

77 dexamethasone.rn,nm.

78 ((intravitreal or intra-vitreal) adj3 (corticoid* or corticosteroid* or steroid*)).tw,kw.

79 or/62-78

80 exp Injections/

81 Drug Implants/

82 (depot or implant* or infus* or inject* or intravitreal* or intra-vitreal* or microsphere* or micro-sphere* or suspension*).tw,kw.

83 or/80-82

84 79 and 83

85 21 and 84

86 (controlled clinical trial or randomized controlled trial).pt.

87 clinical trials as topic.sh.

88 (randomi#ed or randomly or RCT$1 or placebo*).tw.

89 ((singl* or doubl* or trebl* or tripl*) adj (mask* or blind* or dumm*)).tw.

90 trial.ti.

91 or/86-90

92 (48 or 61 or 85) and 91

93 exp Animals/ not (exp Animals/ and Humans/)

94 92 not 93

95 (comment or editorial or interview or news).pt.

96 (letter not (letter and randomized controlled trial)).pt.

97 94 not (95 or 96)

98 97 use prmz [MEDLINE RECORDS]

99 macular degeneration/

100 age related macular degeneration/

101 wet macular degeneration/

102 ((exudative or neovascular or wet) adj3 ((macula* adj2 degeneration) or (macula* adj2 deterioration) or maculopath* or (macula* adj2 dystroph*) or (macula* adj2 atroph*))).tw,kw.

103 ((exudative or neovascular or wet) adj2 (AMD or ARMD)).tw,kw.

104 (wAMD or wARMD).tw,kw.

105 diabetic retinopathy/

106 ((diabet* or DM) adj3 (maculopath* or retinopath*)).tw,kw.

107 diabetic macular edema/

108 (PDR or DME or DMO).tw,kw.

109 exp macular edema/

110 ((macula* or retina*) adj3 (edema$1 or oedema$1)).tw,kw.

111 (Irvine-Gass adj3 (edema$1 or oedema$1 or syndrome$1)).tw,kw.

112 (cystoid macula* adj dystroph*).tw,kw.

113 exp retina vein occlusion/

114 (retinal vein adj3 (occlu* or obstruct* or clos* or stricture* or steno* or block* or embolism*)).tw,kw.

115 (BRVO or CRVO).tw,kw.

116 subretinal neovascularization/

117 ((choroid* or subretinal or sub-retinal) adj1 neovasculari#ation*).tw,kw.

118 CNV.tw,kw.

119 or/99-118

120 vasculotropin inhibitor/

121 (anti adj2 VEGF$1).tw,kw.

122 (antiVEGF$1 or VEGF inhibitor* or VEGF antagonist*).tw,kw.

123 (antivascular endothelial growth factor$1 or anti-vascular endothelial growth factor$1).tw,kw.

124 monoclonal antibody/

125 (monoclonal antibod* and humani#ed).tw,kw.

126 (antibod* adj2 humani#ed).tw,kw.

127 angiogenesis inhibitor/

128 (angiogen* adj3 (inhibitor* or antagonist*)).tw,kw.

129 (anti-angiogen* or antiangiogen*).tw,kw.

130 aflibercept/

131 (aflibercept or "AVE 0005" or AVE0005 or "AVE 005" or AVE005 or "Bay 86-5321" or "Bay86-5321" or Eylea or "UNII-15C2VL427D" or Zaltrap or ZIV-aflibercept).tw,kw.

132 ((vasculotropin or vascular endothelial growth factor or VEGF) adj trap*).tw,kw.

133 aflibercept.rn.

134 bevacizumab/

135 (bevacizumab or Altuzan or Avastin or "nsc 704865" or nsc704865 or "rhuMAb-VEGF" or "UNII-2S9ZZM9Q9V").tw,kw.

136 IVB injection$1.tw,kw.

137 Bevacizumab.rn.

138 pegaptanib/

139 (Pegaptanib or "EYE 001" or EYE001 or Macugen or "NX 1838" or NX1838 or "UNII-3HP012Q0FH").tw,kw.

140 Pegaptanib.rn.

141 ranibizumab/

142 (Ranibizumab or Lucentis or "rhuFab V2" or "UNII-ZL1R02VT79").tw,kw.

143 IVR injection$1.tw,kw.

144 Ranibizumab.rn.

145 or/120-144

146 119 and 145

147 photodynamic therapy/

148 photosensitizing agent/

149 photochemotherapy/

150 (photochemo* or photo-chemo* or photodynamic* or photo-dynamic* or photosensiti* or photo-sensiti*).tw,kw.

151 PDT.tw,kw.

152 or/147-151

153 verteporfin/

154 (verteporphin or "BPD-MA" or "CL 318,952" or "CL 318952" or "UNII-0X9PA28K43" or Visudyne).tw,kw.

155 verteporfin.rn.

156 or/153-155

157 152 and 156

158 (PDTV or "PDT-V" or VPDT or "V-PDT").tw,kw.

159 157 or 158

160 119 and 159

161 triamcinolone/

162 triamcinolone acetonide/

163 ((Triamcinol* adj acet*) or (Triamcincol* adj acet*) or (Triamsinol* adj acet*) or Acetospan or Adcortyl or AllerNaze or Aristocort or Aristoderm or Aristogel or Aristospan or Asmacort or Azmacort or "BRN 0060069" or "CCRIS 5231" or Cinonide or Clinacort or "Coupe-A" or "EINECS 200-948-7" or Flutex or Flutone or FX006 or Kenacort* or Kenalog* or Kenalone or Kenlog or Nasacort or "NSC 21916" or Omcilon or Oracort or Oralone or Polcortolon or Rineton or Solodelf or Tramacin or Triacet$2 or Triacort or Triamcot or Triam-Forte or Triam-Injekt or Triamonide or Trianex or Triatex or Tricinolon or Tricort* or Triderm or Triesence or Triesense or Tri-nasal or Tristoject or Trivaris or Trymex or "UNII-F446C597KA" or Volon).tw,kw.

164 triamcinolone.rn.

165 triamcinolone acetonide.rn.

166 glucocorticoid/

167 (glucocorticoid* or glucorticoid*).tw,kw.

168 anecortave/

169 (anecortave or "AL 3789" or AL3789 or "EINECS 231-812-5" or "NSC 15475" or "NSC 24345" or Retaane or "UNII-Y0PC411K4T").tw,kw.

170 anecortave.rn.

171 fluocinolone acetonide/

172 ((Fluocinolon* adj Acet*) or Alvadermo or Capex or Co-Fluocin or Cortiespec or "EINECS 200-668-5" or Flucinar or Fluocid or Flucort or Fluocet or Fluonid or Fluotrex or (Fluortriamcinolon* adj Acet*) or Flurosyn or Flusolgen or Gelidina or Iluvien or Jellin or Jellisoft or Percutina or Radiocin or Retisert or Sinalar or Synalar or Synamol or Synandone or Synandrone or Synamol or Synemol or Synsac or Tefunote or "UNII-0CD5FD6S2M").tw,kw.

173 fluocinolone acetonide.rn.

174 pregnane derivative/

175 (dihydroxypregnadiene* or di-hydroxypregnadiene* or pregnadienediol*).tw,kw.

176 dexamethasone/

177 dexamethasone isonicotinate/

178 (Dexamethasone or Decaject* or Decameth or Dexasone or Dexpak or Hexadecadrol or Hexadrol or Maxidex or Millicorten or Oradexon or Ozurdex).tw,kw.

179 dexamethasone.rn.

180 dexamethasone isonicotinate.rn.

181 ((intravitreal or intra-vitreal*) adj3 (corticoid* or corticosteroid* or steroid*)).tw,kw.

182 or/161-181

183 exp injection/

184 drug implant/

185 intravitreal drug administration/

186 vi.fs.

187 (depot or implant* or infus* or inject* or intravitreal* or intra-vitreal* or microsphere* or micro-sphere* or suspension*).tw,kw.

188 or/183-187

189 182 and 188

190 119 and 189

191 randomized controlled trial/ or controlled clinical trial/

192 exp "clinical trial (topic)"/

193 (randomi#ed or randomly or RCT$1 or placebo*).tw.

194 ((singl* or doubl* or trebl* or tripl*) adj (mask* or blind* or dumm*)).tw.

195 trial.ti.

196 or/191-195

197 (146 or 160 or 190) and 196

198 exp animal experimentation/ or exp models animal/ or exp animal experiment/ or nonhuman/ or exp vertebrate/

199 exp humans/ or exp human experimentation/ or exp human experiment/

200 198 not 199

201 197 not 200

202 editorial.pt.

203 letter.pt. not (letter.pt. and randomized controlled trial/)

204 201 not (202 or 203)

205 204 use oemezd [EMBASE RECORDS]

206 98 or 205 [BOTH DATABASES]

207 remove duplicates from 206 [TOTAL UNIQUE RECORDS]

208 207 use prmz [UNIQUE MEDLINE RECORDS]

209 207 use oemezd [UNIQUE EMBASE RECORDS]

# eTable 1: Recommended Dosage of Anti-VEGF Agents for Treatment of Wet AMD

| **Anti-VEGF agent** | **Recommended dosage** | **Source** |
| --- | --- | --- |
| Aflibercept | 2 mg (0.05 mL or 50 microliters) of aflibercept administered by intravitreal injection every month (4 weeks) for the first 3 months, followed by 2 mg (0.05 mL) via intravitreal injection every 2 months (8 weeks).  After the first 12 months of treatment with EYLEA, the treatment interval could be extended up to every 3 months (12 weeks) based on visual and/or anatomic outcomes. | Product monograph[1] |
| Ranibizumab | 0.5 mg of ranibizumab is recommended to be administered by intravitreal injection once a month. Treatment may be reduced to one injection every 3 months after the first three injections if monthly dosing is not feasible. Compared to monthly dosing, dosing every 3 months will lead to an approximate 5-letter (1 line) loss of visual acuity benefit, on average, over the following 9 months. | Product monograph[2] |
| Bevacizumab | Note this drug is repackaged and used off-label for the treatment of wet age-related macular degeneration (AMD).  1.25 mg (in 0.05mL of solution) administered by intravitreal injection once monthly.  The need to repackage the drug from the available size vial into a smaller dose increases risk for transmission of infection if improper aseptic technique occurs. | CATT research group[3] |

# eTable 2: Study Characteristics

| **Author, Year** | **Trial Name** | **Trial Identifier** | **Country of Conduct** | **Study Design** | **Study Period** | **Single or Multi-Centre** | **Overall Sample Size** | **Study Duration (Months)** |
| --- | --- | --- | --- | --- | --- | --- | --- | --- |
| Ahmadieh, 2011[4] | NR | NCT00370370 | Iran | Parallel RCT | NR | MULTI | 120 | 6 |
| Amarakoon, 2019[5] | NR | NTR1174 | Netherlands | Parallel RCT | Mar 2010- Dec 2012 | SINGLE | 120 | 12 |
| Arias, 2006[6] | NR | NR | Spain | Parallel RCT | 2004 | SINGLE | 61 | 12 |
| Arnold, 2001[7] | Verteporfin in Photodynamic Therapy Study | BPD OCR 003 | United Kingdom, United States, Spain, Germany, Geneva, Switzerland, England, Sweden, France, Canada, Italy, Austria | Parallel RCT | 1998 | MULTI | 339 | 24 |
| Azab, 2005[8] | Visudyne in Minimally Classic Choroidal Neovascularization Study | BPD OCR 011 | Canada, United States, Czech Republic, Finland, Italy, England | Parallel RCT | 2001-2002 | MULTI | 117 | 24 |
| Barikian, 2015[9] | NR | NR | Lebanon | Parallel RCT | 2010-2012 | SINGLE | 90 | 12 |
| Bashshur, 2007[10] | NR | NR | Lebanon | Parallel RCT | 2005 | SINGLE | 64 | 6 |
| Berg, 2015[11] | LUCAS | NCT01127360 | Norway | Parallel RCT | Mar 2009 to Jul 2012 | MULTI | 441 | 12 |
| Biswas, 2011a[12] | NR | NR | India | Parallel RCT | 2007-2009 | MULTI | 60 | 18 |
| Biswas, 2011b[13] | NR | NR | India | Parallel RCT | NR | MULTI | 120 | 18 |
| Boyer, 2009[14] | SAILOR | NCT00251459 | United States | Parallel RCT | NR | MULTI | 4300 | 12 |
| Bressler, 2001[15] | TAP Study | NR | United States, England, Germany, Switzerland, Canada | Parallel RCT | 1996-1997 | MULTI | 609 | 24 |
| Brown, 2011[16] [CR: Heier, 2011[17]] | CLEAR-IT 2 | NCT00320788 | United States | Parallel RCT | 2006-2008 | MULTI | 159 | 3.67975 |
| Busbee, 2013[18] | HARBOR | NCT00891735 | United States | Parallel RCT | 2009-2010 | MULTI | 1098 | 12 |
| Chakravarthy, 2013[19] | IVAN | ISRCTN92166560 | United Kingdom | Parallel RCT | Mar 27, 2008 - Oct 15, 2010 | MULTI | 610 | 24 |
| Chan, 2015[20] | NR | NCT00749021 | United States | Parallel RCT | 2012 | MULTI | 40 | 12 |
| Chaudhary, 2007[21] | NR | NR | Canada | Parallel RCT | NR | SINGLE | 30 | 12 |
| Chen, 2010[22] | LUV | NR | United States | Parallel RCT | NR | SINGLE | 7 | 12 |
| Costagliola, 2010[23] | NR | NR | Italy | Parallel RCT | 2007-2008 | MULTI | 85 | 12 |
| Danis, 2000[24] | NR | NR | United States | Parallel RCT | NR | SINGLE | 30 | 6 |
| Datseris, 2015[25] | NR | NR | Greece | Parallel RCT | NR | SINGLE | 100 | 12 |
| Dugel, 2019a[26] | HARRIER | NCT02434328 | Singapore, Malaysia, South Korea, Taiwan, Vietnam, Greece, Brazil, Austria, Switzerland, Germany, the Netherlands, Norway, Belgium, Croatia, Turkey, United Kingdom, Czech Republic, Denmark, Estonia, Finland, France, Germany, Hungary, Ireland, Italy, Latvia, Lithuania, Poland, Portugal, Russia, Slovakia, Spain, Sweden | Parallel RCT | 2015-2016 | MULTI | 743 | 12 |
| Dugel 2019b[26] | HAWK | NCT02307682 | Japan, Australia, New Zealand, Israel, Argentina, Columbia, Panama, USA, Canada, Mexico, Puerto Rico | Parallel RCT | 2014-2016 | MULTI | 1082 | 12 |
| Dunavoelgyi, 2011[27] | NR | NR | Austria | Parallel RCT | NR | SINGLE | 40 | 12 |
| Eldem, 2014[28] | SALUTE | NCT01148511 | Turkey | Parallel RCT | 2010-2012 | MULTI | 93 | 12 |
| El-Mollayess, 2012[29] | NR | NR | Lebanon | Parallel RCT | 2009 | MULTI | 120 | 12 |
| Feltgen, 2017[30] | RABIMO | Eudra-CT number: 2009-017324-11 | Germany | Parallel RCT | Apr 2010 - Aug 2013 | SINGLE | 40 | 12 |
| Fung, 2012[31] | LAST | NR | United States | Parallel RCT | NR | SINGLE | 9 | 12 |
| Gharbiya, 2018[32] | NR | NR | Italy | Parallel RCT | October 2014-January 2016 | SINGLE | 76 | 3 |
| Gillies, 2003[33] [CR: Gillies, 2004[34]] | NR | NR | Australia | Parallel RCT | 1996-2000 | SINGLE | 139 | 12 |
| Gillies, 2019[35] | RIVAL | NCT02130024 | Australia | Parallel RCT | 2014/04 - 2017/11 | MULTI | 281 | 12 |
| Giustolisi, 2011[36] | NR | NR | Italy | Parallel RCT | NR | SINGLE | 47 | 6 |
| Guymer, 2019[37] | FLUID | NCT01972789 | Australia | Parallel RCT | 2013-2015 | MULTI | 349 | 24 |
| Haga, 2018[38] | NR | UMIN ID: 000014946 | Japan | Parallel RCT | 2014/08 - 2016/10 | MULTI | 41 | 12 |
| Hatz, 2015[39] | NR | NCT00429962 | Switzerland | Parallel RCT | 2006-2008 | SINGLE | 40 | 12 |
| Heier, 2012[40] - VIEW 1 | VIEW 1 | NCT00509795 | United States, Canada | Parallel RCT | Aug. 2007 - Sep. 2010 | MULTI | 1217 | 12 |
| Heier, 2012[40] - VIEW 2 | VIEW 2 | NCT00637377 | Argentina, Australia, Austria, Belgium, Brazil, Colombia, Czech Republic, France, Germany, Hungary, India, Israel, Italy, Japan, Republic of Korea, Latvia, Mexico, Netherlands, Poland, Portugal, Singapore, Slovakia, Spain, Sweden, Switzerland, United Kingdom, | Parallel RCT | Apr. 2008 - Sep. 2010 | MULTI | 1240 | 12 |
| Hudson, 2011[41] | RADICAL | NCT00492284 | United States, Canada | Parallel RCT | 2007-2010 | MULTI | 162 | 24 |
| Kaiser, 2009[42] | VIO | NCT00121407 | United States, Canada | Parallel RCT | 2002-2005 | MULTI | 364 | 24 |
| Kaiser, 2012[43] | DELANI | NCT00436553 | United States, Canada | Parallel RCT | 2007-2009 | MULTI | 321 | 12 |
| Kertes, 2019[44] | CANTREAT | NCT02103738 | Canada | Parallel RCT | NR | MULTI | 580 | 12 |
| Kodjikian, 2013[45] | GEFAL | NCT01170767 | France | Parallel RCT | 2009 - 2012 | MULTI | 501 | 12 |
| Krebs, 2013a[46] | MANTA | NCT00710229 | Austria | Parallel RCT | 2008-2011 | MULTI | 321 | 12 |
| Krebs, 2013b[47] | Lucentis KAV | NCT01570608 | Austria | Parallel RCT | Mar 2007-Aug 2011 | MULTI | 51 | 12 |
| Kupperman, 2015[48] | NR | NCT00511706 | United States, Australia, France, Israel, Italy, Korea, Portugal, United Kingdom | Parallel RCT | 2007-2009 | MULTI | 243 | 5.75 |
| Lai, 2009[49] | NR | NR | Hong Kong | Parallel RCT | Oct 2006 - Dec 2007 | MULTI | 50 | 6 |
| Larsen, 2012[50] | MONT BLANC | NCT00433017 | Austria, Belgium, Denmark, France, Germany, Hungary, Italy, Netherlands, Poland, Spain, Switzerland, United Kingdom | Parallel RCT | May 2007 - Jul 2009 | MULTI | 255 | 12 |
| Lazic, 2007[51] | NR | NR | Croatia | Parallel RCT | Feb-June 2006 | SINGLE | 165 | 3 |
| Lee, 2007[52] | NR | NR | Korea | Parallel RCT | Jul 2004 - Apr 2005 | SINGLE | 39 | 12 |
| Li, 2012* [53] | NRTTB | NCT01306591 | China | Cluster RCT | Jan 2008 - Jan 2010 | MULTI | 185 | 11.04 |
| Li, 2017[54] | SIGHT | NCT01482910 | China | Parallel RCT | 2011-2014 | MULTI | 304 | 12 |
| Lim, 2015[55] | NR | NCT01175395 | United States | Parallel RCT | 2010-2013 | SINGLE | 10 | 12 |
| Liu, 2019[56] | PHOENIX | NCT01436864 | China | Parallel RCT | Aug 2011 to Sep 2013 | MULTI | 124 | 12 |
| Lushchyk, 2013[57] | NR | NTR1174 | The Netherlands | Parallel RCT | Jun 2008 - Mar 2011 | SINGLE | 191 | 12 |
| Maberley, 2009[58] | Canadian Retinal Trial | NCT00148551 | Canada | Parallel RCT | Jan 2004 - Mar 2008 | MULTI | 100 | 12 |
| Mahmood, 2015[59] | GMAN | ISRCTN34221234 | United Kingdom | Parallel RCT | Feb 2008 - May 2013 | SINGLE | 331 | 21.16 |
| Mantel, 2016[60] | NR | NR | Switzerland | Parallel RCT | NR | SINGLE | 19 | 12 |
| Martin, 2011[61] | CATT | NCT00593450 | United States | Parallel RCT | 2008 - 2010 | MULTI | 1208 | 12 |
| Menon, 2013[62] | BeMOc | EUDRACT No: 2006-003033-33, ISRCTN number: 12980412 | United Kingdom | Parallel RCT | Nov 2006 - Nov 2008 | SINGLE | 100 | 12 |
| Michels, 2005[63] | NR | NR | Austria, Germany, United Kingdom, Switzerland | Parallel RCT | Aug 2000 - May 2002 | MULTI | 203 | 12 |
| Modarres, 2009[64] | NR | NR | Iran | Parallel RCT | Mar - Dec 2006 | SINGLE | 100 | 5 |
| Mori, 2017[65] | NR | NR | Japan | Parallel RCT | NR | SINGLE | 58 | 12 |
| Motarjemizadeh, 2018[66] | NR | NR | Iran | Parallel RCT | Apr 2005 to Mar 2015 | SINGLE | 142 | 12 |
| Nguyen, 2012[67] | CLEAR-IT 1 | NR | United States | Parallel RCT | NR | MULTI | 28 | 2 |
| NR, 2012[68] | NR | NCT00473642 | United States | Parallel RCT | 2007-2009 | SINGLE | 31 | 12 |
| NR, 2015[69] | NR | NCT00896779 | US | Parallel RCT | Oct 2009-Sep 2013 | NR | 20 | 13 |
| NR, 2016[70] | NR | NCT01482910 | China | Parallel RCT | 2011-2014 | MULTI | 304 | 11 |
| NR, 2017[71] | TREND | NCT01948830 | US | Parallel RCT | Dec 2013-Nov 2015 | MULTI | 650 | 12 |
| Parodi, 2012[72] | NR | NCT01327222 | Italy | Parallel RCT | NR | SINGLE | 21 | 6 |
| Piermarocchi, 2008[73] | NR | NR | Italy | Parallel RCT | NR | SINGLE | 84 | 24 |
| Piri, 2014[74] | NR | NCT00370539 | Iran | Parallel RCT | NR | MULTI | 84 | 12 |
| Potter, 2010[75] | VIA | NR | Canada | Parallel RCT | Aug 2006 - Jun 2007 | SINGLE | 36 | 6 |
| Ranchod, 2013[76] | LUCEDEX | NCT00793923 | United States | Parallel RCT | Jan 2008 - Dec 2012 | SINGLE | 40 | 12 |
| Regillo, 2008[77] | PIER | NCT00090623 | United States | Parallel RCT | 2004-2007 | MULTI | 184 | 24 |
| Rezar-Dreindl, 2016[78] | NR | NCT01162746 | Austria | Parallel RCT | Oct 2011-Dec 2015 | SINGLE | 40 | 12 |
| Riazi-Esfahani, 2008[79] | NR | NR | Iran | Parallel RCT | Dec. 2005 to Apr 2006 | MULTI | 104 | 1.4 |
| Rosenfeld, 2006a[80] [CR: Chang, 2007[81]] | MARINA | NCT00056836 | United States | Parallel RCT | 2003-2005 | MULTI | 716 | 24 |
| Rosenfeld, 2006b[82] | NR | NR | United States | Parallel RCT | Mar 2002 - Oct 2003 | MULTI | 32 | 4.6 |
| Rosenfeld, 2007[83] | VALIO | NR | United States | Parallel RCT | Dec 2001 - Aug 2002 | MULTI | 60 | 12 |
| Sacu, 2008[84] | NR | EudraCT No.: 2005-000776-41 | Austria | Parallel RCT | NR | SINGLE | 40 | 2.8 |
| Schauwvlieghe, 2016[85] | BRAMD | Netherlands Trial Register: NTR1704 | Netherlands | Parallel RCT | Jan 2009 - Dec 2011 | MULTI | 332 | 12 |
| Schmidt-Erfurth, 2008[86] | the Early Retreatment Study Group | NR | Austria | Parallel RCT | Aug 2000 and May 2002 | MULTI | 203 | 24 |
| Schmidt-Erfurth, 2011[87] | the EXCITE study | NCT00275821 | Austria | Parallel RCT | 2005-2008 | MULTI | 353 | 12 |
| Scholler, 2014[88] | NR | EK-07-192-1007/ EudraCT Nr. 2007-005157-33). | Austria | Parallel RCT | 2008 - 2011 | SINGLE | 55 | 12 |
| Semeraro, 2015[89] | NR | NR | Italy | Parallel RCT | NR | MULTI | 75 | 12 |
| Silva, 2018[90] | TREND | NCT01948830 | Belgium, Croatia, Denmark, Egypt, Germany, Hungary, India, Israel, Italy, Korea, Portugal, Russia, Slovakia, Slovenia, Spain, Switzerland, Turkey, UK | Parallel RCT | 2013-2015 | MULTI | 650 | 12 |
| Subramanian, 2010[91] | NR | ISRCTN73359806 | United States | Parallel RCT | 2007-2009 | SINGLE | 28 | 12 |
| Tano, 2010[92] | EXTEND-I | NR | Japan | Parallel RCT | NR | MULTI | 76 | 12 |
| Vallance, 2010[93] | NR | ISRCTN42639823 | United Kingdom | Parallel RCT | 2007-2009 | SINGLE | 18 | 12 |
| Weingessel, 2015[94] | NR | NR | Austria | Parallel RCT | NR | SINGLE | 34 | 12 |
| Williams, 2012[95] | NR | NR | United States | Parallel RCT | NR | MULTI | 60 | 12 |
| Wykoff, 2015[96] | TREX-AMD | NCT01748292 | United States | Parallel RCT | 2012-2017 | MULTI | 60 | 12 |

**Abbreviations:** NR, not reported; RCT, randomized controlled trial

* this study was a cluster RCT, all other included studies were parallel RCTs

# eTable 3: Patient Characteristics

| **Author, Year** | **# of eyes** | **Overall mean age** | **Overall mean age variance type** | **Overall mean age variance value** | **% female** | **Lens status of patients** |
| --- | --- | --- | --- | --- | --- | --- |
| Ahmadieh 2011[4] | 115 | 71.40 | SD | 7.60 | 60.00 | pseudophakic |
| Amarakoon, 2019[5] | 120 | NR | NR | NR | 61.6 | NR |
| Arias 2006[6] | 61 | NR | NR | NR | 50.82 | mixture of phakic/ pseudophakic/ cataract |
| Arnold 2001[7] | 339 | NR | NR | NR | 60.00 | NR |
| Azab 2005[8] | 117 | NR | NR | NR | 64.00 | NR |
| Barikian 2015[9] | 90 | 76.70 | range | 56.00-90.00 | 45.56 | NR |
| Bashshur 2007[10] | 64 | NR | NR | NR | 50.00 | NR |
| Berg 2015[11] | NR | NR | SD | NR | NR | NR |
| Biswas 2011a[12] | 60 | 60.00 | NR | NR | NR | NR |
| Biswas 2011b[13] | 104 | NR | NR | NR | 52.00 | NR |
| Boyer 2009[14] | 4300 | 79.00 | SD | NR | 59.00 | NR |
| Bressler 2001[15] | 609 | NR | NR | NR | 56.49 | NR |
| Brown 2011[16] [CR: Heier 2011[17]] | 159 | 78.20 | range | 53.00-94.00 | 62.00 | NR |
| Busbee 2013[18] | 1097 | 79.00 | SD | NR | 59.00 | NR |
| Chakravarthy 2013[19] | NR | 77.70 | SD | 7.40 | 60.00 | NR |
| Chan 2015[20] | 36 | NR | SD | NR | 72.00 | NR |
| Chaudhary 2007[21] | 30 | NR | SD | NR | 53.00 | phakic |
| Chen 2010[22] | 7 | 76.00 | range | 66.00-80.00 | 0.00 | NR |
| Costagliola 2010[23] | 85 | NR | SD | NR | 55.00 | mixture of phakic/ pseudophakic/ cataract |
| Danis 2000[24] | 27 | NR | NR | NR | 56.00 | NR |
| Datseris 2015[25] | 95 | 74.00 | SD | 9.40 | 69.00 | NR |
| Dugel, 2019a[26] | 743 | 75.1 | SD | 8.24 | 57.1 | NR |
| Dugel 2019b[26] | 1082 | 76.5 | SD | 8.68 | 56.5 | NR |
| Dunavoelgyi, 2011[27] | 40 | NR | SD | NR | NR | NR |
| Eldem 2014[28] | 93 | NR | range | NR | 46.24 | NR |
| El-Mollayess 2012[29] | 120 | 76.80 | NR | NR | 65.00 | NR |
| Feltgen 2017[30] | 40 | 79 | IQR | 12 | 65.00 | NR |
| Fung 2012[31] | 9 | NR | NR | NR | 66.67 | NR |
| Gharbiya, 2018[32] | 76 | 78.6 | range | 54-94 | 65.8 | mixture of phakic and pseudophakic |
| Gillies 2003[33] [CR: Gillies, 2004[34]] | 151 | NR | NR | NR | 60.93 | mixture of phakic, pseudophakic, cataract |
| Gillies, 2019[35] | 281 | 77.7 | SD | 8.1 | 52.7 | NR |
| Giustolisi 2011[36] | 47 | NR | NR | NR | 48.94 | NR |
| Guymer, 2019[37] | 349 | 79 | SD | 8.1 | 54.50 | NR |
| Haga, 2018[38] | 41 | NR | NR | NR | 31.70 | NR |
| Hatz 2014[39] | 40 | NR | NR | NR | 68.00 | NR |
| Heier 2012[40] - VIEW 1 | 1210 | NR | SD | NR | 58.76 | NR |
| Heier 2012[40] - VIEW 2 | 1202 | NR | SD | NR | 55.49 | NR |
| Hudson 2011[41] | 162 | 79.00 | SD | 7.00 | 58.00 | NR |
| Kaiser 2009[42] | 364 | 79.00 | NR | NR | 62.64 | NR |
| Kaiser 2012[43] | 321 | NR | SD | NR | 59.81 | NR |
| Kertes, 2019[44] | 580 | 78.8 | SD | 7.8 | 60.30 | NR |
| Kodjikian 2013[45] | 501 | NR | NR | NR | 66.00 | NR |
| Krebs 2013a[46] | 317 | NR | SD | NR | 63.70 | NR |
| Krebs 2013b[47] | 51 | 78.86 | SD | 7.83 | NR | mixture of phakic, pseudophakic, cataract |
| Kupperman 2015[48] | 243 | NR | NR | NR | 60.00 | NR |
| Lai 2009[49] | 50 | 75.80 | SD | 7.40 | 32.00 | mixture of phakic, pseudophakic, cataract |
| Larsen 2012[50] | 255 | NR | SD | NR | 59.61 | NR |
| Lazic 2007[51] | 165 | 75.70 | SD | 6.00 | 64.24 | NR |
| Lee 2007[52] | 39 | 69.08 | SD | 9.24 | 32.43 | pseudophakic |
| Li 2012[53] | 185 | Median 69 | NR | NR | 34.05 | NR |
| Li, 2017[54] | 304 | 65.1 | SD | 8.7 | 35.50 | NR |
| Lim 2015[55] | 10 | 73.40 | range | 59.00-89.00 | 60.00 | NR |
| Liu, 2019[56] | 124 | NR | MR | NR | 32.30 | NR |
| Lushchyk 2013[57] | 191 | NR | SD | NR | 66.00 | NR |
| Maberley 2009[58] | 100 | NR | range | NR | 56.00 | mixture of phakic, pseudophakic, cataract |
| Mahmood 2015[59] | 331 | NR | IQR | NR | 61.00 | NR |
| Mantel 2016[60] | 21 | 76.00 | SD | 23.50 | NR | NR |
| Martin 2011[61] | 1208 | NR | NR | NR | 62.00 | NR |
| Menon 2013[62] | 100 | NR | NR | NR | 72.00 | NR |
| Michels 2005[63] | 203 | NR | NR | NR | NR | NR |
| Modarres 2009[64] | 86 | NR | SD | NR | 32.56 | NR |
| Mori, 2017[65] | 40 | NR | NR | NR | 72.50 | NR |
| Motarjemizadeh, 2018[66] | 136 | 71.1 | SD | 8.4 | 45.60 | mixture of phakic and pseudophakic |
| Nguyen 2012[67] | 28 | 76.30 | SD | NR | 46.43 | NR |
| NR 2012[68] | 31 | 74.00 | SD | 2.70 | 74.00 | NR |
| NR 2015[69] | 20 | 83 | range | 68-97 | 55.00 | NR |
| NR 2016[70] | 304 | 65.10 | SD | 8.70 | 35.53 | NR |
| NR 2017[71] | 650 | 75.2 | SD | 8.37 | 55.00 | NR |
| Parodi 2012[72] | 21 | 71.50 | SD | 4.20 | 61.90 | NR |
| Piermarocchi 2008[73] | 84 | NR | SD | NR | 70.24 | pseudophakic |
| Piri 2014[74] | 84 | 71.70 | SD | 9.00 | 42.86 | NR |
| Potter 2010[75] | 36 | NR | SD | NR | 68.57 | NR |
| Ranchod 2013[76] | 37 | NR | NR | NR | 64.86 | NR |
| Regillo 2008[77] | 184 | 78.00 | NR | NR | 59.78 | NR |
| Rezar-Dreindl 2016[78] | 40 | NR | SD | NR | 72.50 | NR |
| Riazi-Esfahani 2008[79] | 92 | 70.60 | SD | 8.70 | 42.20 | NR |
| Rosenfeld 2006a[80] [CR: Chang 2007[81]] | 716 | NR | SD | NR | 64.80 | NR |
| Rosenfeld 2006b[82] | 32 | 79.70 | SD | 5.00 | 62.50 | NR |
| Rosenfeld 2007[83] | 60 | NR | NR | NR | 58.33 | NR |
| Sacu 2008[84] | 40 | NR | SD | NR | 65.00 | NR |
| Schauwvlieghe 2016[85] | 332 | 78 | SD | 7 | 56.00 | pseudophakic |
| Schmidt-Erfurth 2008[86] | 203 | NR | NR | NR | NR | NR |
| Schmidt-Erfurth 2011[87] | 353 | 75.30 | SD | 7.56 | 59.20 | NR |
| Scholler 2014[88] | 55 | NR | SD | NR | 70.91 | NR |
| Semeraro 2015[89] | 75 | NR | SD | NR | 52.00 | NR |
| Silva, 2018[90] | 650 | NR | NR | NR | 55.40 | NR |
| Subramanian 2010[91] | 28 | 78.59 | SD | NR | 4.55 | NR |
| Tano 2010[92] | 76 | NR | NR | NR | 22.00 | NR |
| Vallance 2010[93] | 18 | NR | NR | NR | NR | NR |
| Weingessel 2015[94] | 30 | NR | SD | NR | 73.00 | NR |
| Williams 2012[95] | 56 | NR | NR | NR | NR | NR |
| Wykoff 2015[96] | 60 | 77.00 | range | 59.00-96.00 | 63.00 | NR |

**Abbreviations:** NA, not applicable; NR, not reported; SD, standard deviation; Tx, treatment

# eFigure 1: Aggregate Risk of Bias Figure


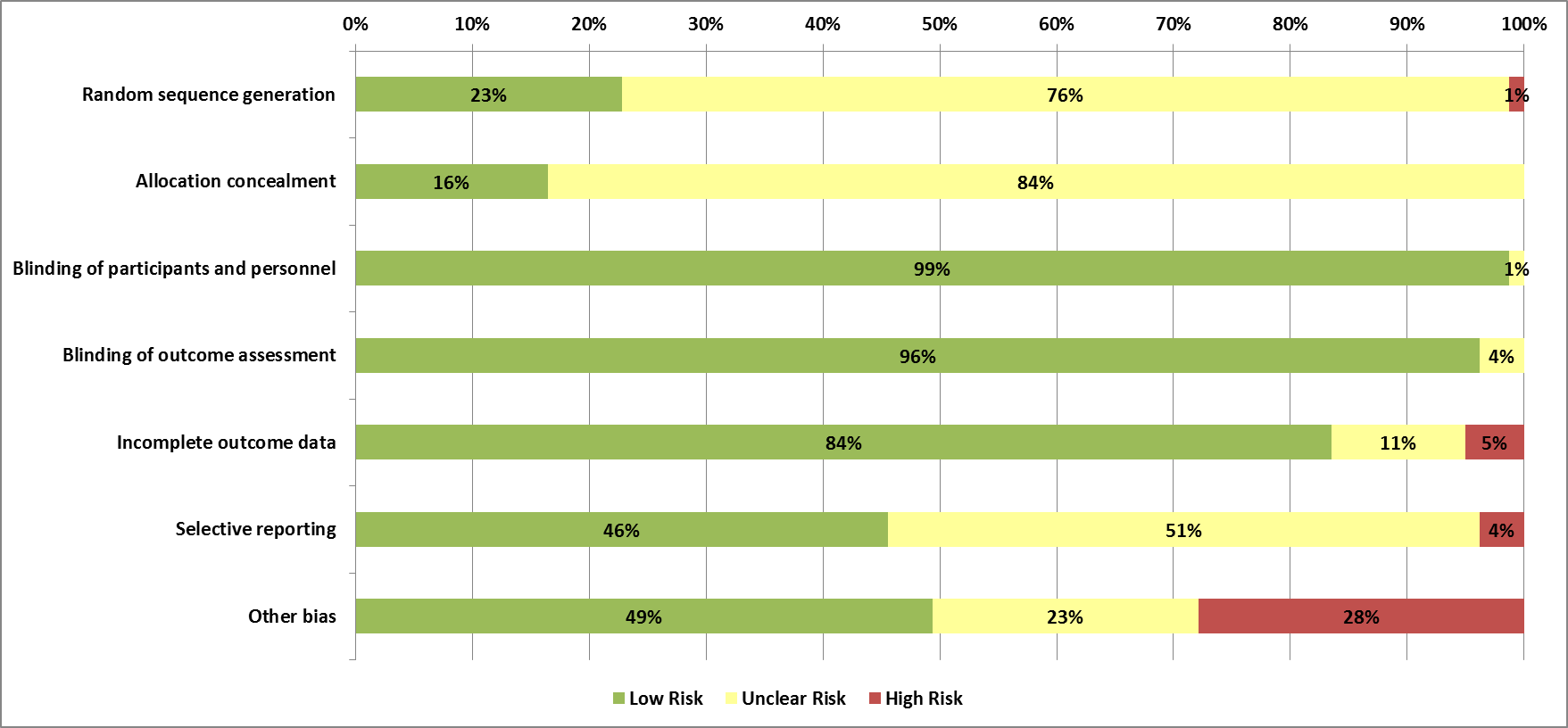


Aggregate summary of Cochrane Risk of Bias assessments across all included studies

# eTable 4: Cochrane Risk of Bias Results for Individual Studies

| **First Author, Year** | **Selection Bias** | | **Performance Bias** | **Detection Bias** | **Attrition Bias** | **Reporting Bias** | **Other Bias** |
| --- | --- | --- | --- | --- | --- | --- | --- |
|  | **Random sequence generation** | **Allocation concealment** | **Blinding of participants and personnel** | **Blinding of outcome assessment** | **Incomplete outcome data** | **Selective reporting** | **Other Bias** |
| Ahmadieh 2011 | Low risk | Unclear risk | Low risk | Low risk | Low risk | Low risk | Low risk |
| Arias 2006 | Unclear risk | Unclear risk | Low risk | Low risk | Low risk | Unclear risk | Unclear risk |
| Arnold 2001 | Unclear risk | Unclear risk | Low risk | Low risk | Low risk | Unclear risk | High risk |
| Azab 2005 | Unclear risk | Unclear risk | Low risk | Low risk | Low risk | Unclear risk | High risk |
| Barikian 2015 | Unclear risk | Unclear risk | Low risk | Low risk | Low risk | Unclear risk | Low risk |
| Bashshur 2007 | Unclear risk | Unclear risk | Low risk | Low risk | Low risk | Unclear risk | Unclear risk |
| Berg 2015 | Low risk | Low risk | Low risk | Low risk | Low risk | Low risk | Low risk |
| Biswas 2011a | Unclear risk | Unclear risk | Low risk | Low risk | Unclear risk | Unclear risk | Unclear risk |
| Biswas 2011b | Low risk | Unclear risk | Low risk | Low risk | Unclear risk | Unclear risk | Low risk |
| Boyer 2009 | Unclear risk | Unclear risk | Low risk | Low risk | Low risk | Low risk | High risk |
| Bressler 2001 | Unclear risk | Unclear risk | Low risk | Low risk | Low risk | Unclear risk | High risk |
| Brown 2011 | Unclear risk | Unclear risk | Low risk | Low risk | Low risk | Low risk | High risk |
| Busbee 2013 | Low risk | Low risk | Low risk | Low risk | Low risk | Low risk | High risk |
| Chakravarthy 2013 | Low risk | Low risk | Low risk | Low risk | Low risk | Low risk | Low risk |
| Chan 2015 | Unclear risk | Unclear risk | Low risk | Low risk | Low risk | Low risk | Low risk |
| Chaudhary 2007 | Unclear risk | Unclear risk | Low risk | Low risk | Low risk | Unclear risk | Unclear risk |
| Chen 2010 | Unclear risk | Unclear risk | Low risk | Low risk | Low risk | Unclear risk | Low risk |
| Costagliola 2010 | Unclear risk | Unclear risk | Low risk | Low risk | Unclear risk | Unclear risk | Unclear risk |
| Danis 2000 | Unclear risk | Unclear risk | Low risk | Low risk | Unclear risk | Unclear risk | Low risk |
| Datseris 2015 | Unclear risk | Unclear risk | Low risk | Low risk | Low risk | Unclear risk | Unclear risk |
| Dugel 2019a | Low risk | Low risk | Low risk | Low risk | Low risk | Low risk | High risk |
| Dugel 2019b | Low risk | Low risk | Low risk | Low risk | Low risk | Low risk | High risk |
| Dunavoelgyi 2011 | Unclear risk | Unclear risk | Low risk | Low risk | Unclear risk | Unclear risk | Unclear risk |
| Eldem 2014 | Unclear risk | Unclear risk | Low risk | Low risk | High risk | Low risk | High risk |
| El-Mollayess 2012 | Low risk | Unclear risk | Low risk | Low risk | Low risk | Unclear risk | Low risk |
| Feltgen 2017 | Unclear risk | Unclear risk | Low risk | Low risk | Low risk | Low risk | High risk |
| Fung 2012 | Unclear risk | Unclear risk | Low risk | Low risk | Low risk | Unclear risk | Low risk |
| Gillies 2003 | Low risk | Low risk | Low risk | Low risk | Low risk | Unclear risk | Low risk |
| Gillies 2019 | Low risk | High risk | High risk | Low risk | Low risk | Low risk | Low risk |
| Giustolisi 2011 | Unclear risk | Unclear risk | Low risk | Low risk | Low risk | Unclear risk | Low risk |
| Hatz 2014 | Unclear risk | Unclear risk | Low risk | Low risk | Low risk | Low risk | Low risk |
| Heier 2012 - VIEW 1 | Unclear risk | Low risk | Low risk | Low risk | Low risk | Low risk | High risk |
| Heier 2012 - VIEW 2 | Unclear risk | Low risk | Low risk | Low risk | Low risk | Low risk | High risk |
| Hudson 2011 | Unclear risk | Unclear risk | Low risk | Low risk | Low risk | Low risk | Low risk |
| Kaiser 2009 | Unclear risk | Unclear risk | Low risk | Low risk | Low risk | Low risk | High risk |
| Kaiser 2012 | Unclear risk | Unclear risk | Low risk | Low risk | Low risk | Low risk | High risk |
| Kodjikian 2013 | Unclear risk | Unclear risk | Low risk | Low risk | Low risk | Low risk | Low risk |
| Krebs 2013a | Unclear risk | Low risk | Low risk | Low risk | Low risk | Low risk | Low risk |
| Krebs 2013b | Low risk | Unclear risk | Low risk | Low risk | Low risk | Low risk | Unclear risk |
| Kupperman 2015 | Unclear risk | Unclear risk | Low risk | Low risk | Low risk | Low risk | High risk |
| Lai 2009 | Low risk | Low risk | Low risk | Low risk | Low risk | Unclear risk | Low risk |
| Larsen 2012 | Unclear risk | Unclear risk | Low risk | Low risk | Low risk | Low risk | High risk |
| Laziv 2007 | Unclear risk | Unclear risk | Low risk | Low risk | Low risk | Unclear risk | Unclear risk |
| Lee 2007 | High risk | Low risk | Low risk | Low risk | Low risk | Unclear risk | Low risk |
| Li 2012 | Low risk | Unclear risk | Low risk | Low risk | High risk | Low risk | Low risk |
| Li 2017 | Low risk | Unclear risk | Low risk | Low risk | Low risk | Low risk | High risk |
| Liu 2019 | Low risk | Low risk | Low risk | Low risk | Low risk | Low risk | Low risk |
| Lim 2014 | Unclear risk | Unclear risk | Low risk | Unclear risk | Low risk | Low risk | High risk |
| Lyshchyk 2013 | Unclear risk | Unclear risk | Low risk | Low risk | High risk | Low risk | Unclear risk |
| Maberley 2009 | Low risk | Low risk | Low risk | Low risk | Low risk | Low risk | Low risk |
| Mahmood 2015 | Low risk | Unclear risk | Low risk | Low risk | Low risk | High risk | Low risk |
| Mantel 2015 | Unclear risk | Unclear risk | Unclear risk | Unclear risk | Low risk | Unclear risk | Low risk |
| Martin 2011 | Unclear risk | Low risk | Low risk | Low risk | Low risk | Low risk | Low risk |
| Menon 2013 | Unclear risk | Unclear risk | Low risk | Low risk | Low risk | High risk | Low risk |
| Michels 2005 | Unclear risk | Unclear risk | Low risk | Low risk | Low risk | Unclear risk | Low risk |
| Modarres 2009 | Low risk | Unclear risk | Low risk | Low risk | Low risk | Unclear risk | Unclear risk |
| Motarjemizadeh 2018 | Low risk | Unclear risk | Low risk | Low risk | Unclear risk | Unclear risk | Low risk |
| Nguyen 2012 | Unclear risk | Unclear risk | Low risk | Low risk | Low risk | Unclear risk | High risk |
| NR 2012 | Unclear risk | Unclear risk | Low risk | Low risk | Low risk | Unclear risk | Low risk |
| NR 2015 | Low risk | Unclear risk | Low risk | Low risk | Low risk | Low risk | Low risk |
| NR 2016 | Unclear risk | Unclear risk | Low risk | Low risk | Low risk | Low risk | Low risk |
| NR 2017 | Unclear risk | Unclear risk | Low risk | Low risk | Low risk | Low risk | Low risk |
| Parodi 2012 | Unclear risk | Unclear risk | Low risk | Low risk | Unclear risk | Low risk | Unclear risk |
| Piermarocchi 2008 | Unclear risk | Unclear risk | Low risk | Low risk | Unclear risk | Unclear risk | Unclear risk |
| Piri 2014 | Unclear risk | Unclear risk | Low risk | Low risk | Low risk | High risk | Low risk |
| Potter 2010 | Unclear risk | Unclear risk | Low risk | Low risk | Low risk | Unclear risk | Low risk |
| Ranchod 2013 | Unclear risk | Unclear risk | Low risk | Low risk | Low risk | Low risk | Low risk |
| Regillo 2008 | Low risk | Unclear risk | Low risk | Low risk | Low risk | Unclear risk | High risk |
| Rezar-Dreindl 2016 | Low risk | Low risk | Low risk | Low risk | Low risk | Low risk | Unclear risk |
| Riazi-Esfahani 2008 | Unclear risk | Unclear risk | Low risk | Low risk | High risk | Unclear risk | Unclear risk |
| Rosenfeld 2006a | Unclear risk | Unclear risk | Low risk | Low risk | Low risk | Unclear risk | High risk |
| Rosenfeld 2006b | Unclear risk | Unclear risk | Low risk | Low risk | Low risk | Unclear risk | High risk |
| Rosenfeld 2007 | Unclear risk | Unclear risk | Low risk | Low risk | Low risk | Unclear risk | Low risk |
| Sacu 2008 | Unclear risk | Unclear risk | Low risk | Low risk | Low risk | Unclear risk | Low risk |
| Schauwvlieghe 2016 | Low risk | Low risk | Low risk | Low risk | Low risk | Low risk | Low risk |
| Schmidt-Erfurth 2008 | Unclear risk | Unclear risk | Low risk | Low risk | Low risk | Unclear risk | Low risk |
| Schmidt-Erfurth 2011 | Unclear risk | Unclear risk | Low risk | Low risk | Low risk | Low risk | High risk |
| Scholler 2014 | Low risk | Unclear risk | Low risk | Low risk | Unclear risk | Low risk | Low risk |
| Semeraro 2015 | Unclear risk | Unclear risk | Low risk | Low risk | Low risk | Unclear risk | Unclear risk |
| Subramanian 2010 | Unclear risk | Unclear risk | Low risk | Low risk | Unclear risk | Low risk | Low risk |
| Tano 2010 | Unclear risk | Unclear risk | Low risk | Low risk | Low risk | Unclear risk | Low risk |
| Vallance 2010 | Unclear risk | Unclear risk | Low risk | Low risk | Low risk | Low risk | Unclear risk |
| Weingessel 2015 | Unclear risk | Unclear risk | Low risk | Low risk | Low risk | Unclear risk | Unclear risk |
| Williams 2012 | Unclear risk | Unclear risk | Low risk | Low risk | Low risk | Unclear risk | High risk |
| Wykoff 2015 | Unclear risk | Unclear risk | Low risk | Unclear risk | Low risk | Unclear risk | High risk |

# eTable 5: Transitivity Assessment for all NMA Outcomes

| ***Treatment Comparison*** | ***Median study duration*** | ***Lens status*** | ***% patients with diabetes*** | ***% patients with hypertension*** | ***Age*** | ***Sample size*** | ***RoB – random sequence generation*** | ***RoB – allocation concealment*** |
| --- | --- | --- | --- | --- | --- | --- | --- | --- |
| ***VISION GAIN*** | | | | | | | | |
| Ranibizumab vs. placebo | 18 months | Not reported | Not reported | Not reported | (≥ 50 years, Not reported) | > 100 | Unclear or high risk | Unclear or high risk |
| PDT vs. placebo | 24 months | Not reported | Not reported | ≥ 40% | Not reported | > 100 | Unclear or high risk | Unclear or high risk |
| Ranibizumab vs. aflibercept | 12 months | Not reported | Not reported | Not reported | Not reported | > 100 | Unclear or high risk | Low risk |
| Ranibizumab vs. bevacizumab | 12 months | Not reported | Not reported | Not reported | Not reported | > 100 | Unclear or high risk | Unclear or high risk |
| Bevacizumab+PDT vs. bevacizumab | 12 months | Not reported | Not reported | Not reported | Not reported | < 100 | Unclear or high risk | Unclear or high risk |
| DXM+PDT+ranibizumab vs. ranibizumab | 24 months | Not reported | Not reported | Not reported | ≥ 50 years | > 100 | Unclear or high risk | Unclear or high risk |
| PDT+ranibizumab vs. ranibizumab | 12 months | Not reported | Not reported | Not reported | Not reported | < 100 | Unclear or high risk | Unclear or high risk |
| DXM+ranibizumab vs. ranibizumab | 9 months | Not reported | Not reported | Not reported | Not reported | (< 100, > 100) | Unclear or high risk | Unclear or high risk |
| IVTA+PDT vs. PDT | 12 months | Phakic/ Pseudophakic | Not reported | Not reported | Not reported | < 100 | Unclear or high risk | Unclear or high risk |
| DXM+PDT+ranibizumab vs. PDT+ranibizumab | 24 months | Not reported | Not reported | Not reported | ≥ 50 years | > 100 | Unclear or high risk | Unclear or high risk |
| ***VISION LOSS*** | | | | | | | | |
| Ranibizumab vs. placebo | 18 months | Not reported | Not reported | Not reported | (≥ 50 years, Not reported) | > 100 | Unclear or high risk | Unclear or high risk |
| PDT vs. placebo | 24 months | Not reported | Not reported | (≥ 40%, Not reported) | Not reported | > 100 | Unclear or high risk | Unclear or high risk |
| IVTA vs. placebo | 12 months | Phakic/ Pseudophakic | Not reported | ≥ 40% | Not reported | > 100 | Low risk | Low risk |
| Ranibizumab vs. aflibercept | 12 months | Not reported | Not reported | Not reported | Not reported | > 100 | Unclear or high risk | Low risk |
| Ranibizumab vs. bevacizumab | 12 months | Not reported | Not reported | Not reported | Not reported | > 100 | Unclear or high risk | Unclear or high risk |
| Bevacizumab+PDT vs. bevacizumab | 12 months | Not reported | Not reported | Not reported | Not reported | < 100 | Unclear or high risk | Unclear or high risk |
| DXM+ranibizumab vs. ranibizumab | 6 months | Not reported | Not reported | Not reported | Not reported | > 100 | Unclear or high risk | Unclear or high risk |
| DXM+PDT+ranibizumab vs. ranibizumab | 24 months | Not reported | Not reported | Not reported | ≥ 50 years | > 100 | Unclear or high risk | Unclear or high risk |
| PDT+ranibizumab vs. ranibizumab | 12 months | Not reported | Not reported | Not reported | ≥ 50 years | < 100 | Unclear or high risk | Unclear or high risk |
| IVTA+PDT vs. PDT | 12 months | Phakic/ Pseudophakic | Not reported | Not reported | Not reported | < 100 | Unclear or high risk | Unclear or high risk |
| DXM+PDT+ranibizumab vs. PDT+ranibizumab | 24 months | Not reported | Not reported | Not reported | ≥ 50 years | > 100 | Unclear or high risk | Unclear or high risk |
| ***Mean BCVA*** | | | | | | | | |
| Ranibizumab vs. placebo | 18 months | Not reported | Not reported | Not reported | (≥50 years, Not reported) | > 100 | Unclear or high risk | Unclear or high risk |
| PDT vs. placebo | 24 months | Not reported | Not reported | Not reported | ≥50 years | > 100 | Unclear or high risk | Unclear or high risk |
| Ranibizumab vs. aflibercept | 12 months | Not reported | Not reported | Not reported | Not reported | > 100 | Unclear or high risk | Unclear or high risk |
| PDT + ranibizumab vs. DXM + PDT + ranibizumab | 24 months | Not reported | Not reported | Not reported | ≥50 years | > 100 | Unclear or high risk | Unclear or high risk |
| Bevacizumab + IVTA vs. bevacizumab | 6 months | Phakic/ pseudophakic | Not reported | Not reported | ≥50 years | > 100 | Low risk | Unclear or high risk |
| Ranibizumab vs. bevacizumab | 12 months | Not reported | Not reported | Not reported | Not reported | > 100 | Unclear or high risk | Unclear or high risk |
| Bevacizumab + PDT vs. bevacizumab | 9 months | (Phakic/ pseudophakic, Not reported) | Not reported | Not reported | Not reported | < 100 | Unclear or high risk | Unclear or high risk |
| DXM + PDT + ranibizumab vs. ranibizumab | 24 months | Not reported | Not reported | Not reported | ≥50 years | > 100 | Unclear or high risk | Unclear or high risk |
| PDT + ranibizumab vs. ranibizumab | 12 months | Not reported | Not reported | Not reported | Not reported | < 100 | Unclear or high risk | Unclear or high risk |
| DXM + ranibizumab vs. ranibizumab | 9 months | Not reported | Not reported | Not reported | Not reported | (< 100,  > 100) | Unclear or high risk | Unclear or high risk |
| IVTA + PDT vs. PDT | 6 months | Not reported | Not reported | Not reported | Not reported | > 100 | Unclear or high risk | Unclear or high risk |
| Bevacizumab + IVTA + PDT vs. bevacizumab + PDT | 12 months | Not reported | Not reported | Not reported | ≥50 years | > 100 | Unclear or high risk | Unclear or high risk |
| ***MORTALITY*** | | | | | | | | |
| Ranibizumab vs. placebo | 18 months | Not reported | Not reported | Not reported | (≥50 years, Not reported) | > 100 | Unclear or high risk | Unclear or high risk |
| PDT vs. placebo | 24 months | Not reported | Not reported | ≥40% | Not reported | > 100 | Unclear or high risk | Unclear or high risk |
| IVTA vs. placebo | 36 months | Not reported | Not reported | ≥40% | Not reported | > 100 | Low risk | Low risk |
| Ranibizumab vs. aflibercept | 12 months | Not reported | Not reported | Not reported | Not reported | > 100 | Unclear or high risk | Low risk |
| PDT vs. aflibercept | 6 months | Not reported | Not reported | Not reported | ≥50 years | > 100 | Unclear or high risk | Unclear or high risk |
| Ranibizumab vs. bevacizumab | 12 months | Not reported | Not reported | Not reported | Not reported | > 100 | Unclear or high risk | Unclear or high risk |
| Bevacizumab + PDT vs. bevacizumab | 6 months | Not reported | Not reported | Not reported | Not reported | < 100 | Unclear or high risk | Unclear or high risk |
| DXM + PDT + ranibizumab vs. ranibizumab | 24 months | Not reported | Not reported | Not reported | ≥50 years | > 100 | Unclear or high risk | Unclear or high risk |
| PDT + ranibizumab vs. ranibizumab | 12 months | Not reported | Not reported | Not reported | Not reported | > 100 | Unclear or high risk | Unclear or high risk |
| IVTA + PDT vs. PDT | 12 months | Phakic/ Pseudophakic | Not reported | Not reported | Not reported | > 100 | Low risk | Low risk |
| DXM + PDT + ranibizumab vs. PDT + ranibizumab | 24 months | Not reported | Not reported | Not reported | ≥50 years | > 100 | Unclear or high risk | Unclear or high risk |
| ***BACTERIAL ENDOPHTHALMITIS*** | | | | | | | | |
| Ranibizumab vs. bevacizumab | 12 months | Not reported | Not reported | (≥40%, Not reported) | Not reported | > 100 | Unclear or high risk | Unclear or high risk |
| Ranibizumab vs. placebo | 24 months | Not reported | Not reported | Not reported | Not reported | > 100 | Unclear or high risk | Unclear or high risk |
| Ranibizumab vs. aflibercept | 12 months | Not reported | Not reported | Not reported | Not reported | > 100 | Unclear or high risk | Low risk |
| ***ADVERSE EVENTS*** | | | | | | | | |
| Ranibizumab vs. bevacizumab | 12 months | Not reported | Not reported | (≥ 40%, Not reported) | Not reported | (<100, >100) | Unclear or high risk | Unclear or high risk |
| Ranibizumab vs. placebo | 24 months | Not reported | Not reported | Not reported | Not reported | >100 | Unclear or high risk | Unclear or high risk |
| PDT vs. placebo | 24 months | Not reported | Not reported | ≥ 40% | Not reported | >100 | Unclear or high risk | Unclear or high risk |
| IVTA vs. placebo | 12 months | Phakic/ pseudophakic | Not reported | ≥ 40% | Not reported | >100 | Low risk | Low risk |
| Aflibercept vs. PDT | 11.05 months | Not reported | Not reported | Not reported | ≥ 50 years | >100 | Unclear or high risk | Unclear or high risk |
| DXM + ranibizumab vs. ranibizumab | 6 months | Not reported | Not reported | Not reported | Not reported | >100 | Unclear or high risk | Unclear or high risk |
| Ranibizumab vs. aflibercept | 12 months | Not reported | Not reported | Not reported | Not reported | >100 | Unclear or high risk | Low risk |
| ***ARTERIAL THROMBOEMBOLIC EVENTS*** | | | | | | | | |
| Ranibizumab vs. placebo | 18 months | Not reported | Not reported | Not reported | (≥50 years, Not reported) | > 100 | Unclear or high risk | Unclear or high risk |
| Ranibizumab vs. aflibercept | 12 months | Not reported | Not reported | Not reported | Not reported | > 100 | Unclear or high risk | Low risk |
| Ranibizumab vs. bevacizumab | 12 months | Not reported | Not reported | Not reported | Not reported | > 100 | Unclear or high risk | Unclear or high risk |
| PDT + ranibizumab vs. ranibizumab | 12 months | Not reported | Not reported | Not reported | Not reported | > 100 | Unclear or high risk | Unclear or high risk |
| DXM + ranibizumab vs. ranibizumab | 6 months | Not reported | Not reported | Not reported | Not reported | > 100 | Unclear or high risk | Unclear or high risk |

Method: For continuous outcomes, e.g. study duration, the median value for all network meta-analyses was reported. For categorical outcomes, e.g. lens status, percentage of patients with diabetes and hypertension, age, and sample size, the mode (most frequent outcome) was reported. For instances where there was no mode, all outcomes in the analysis were reported. For the risk of bias (ROB) assessments, if all studies in the analyses had the same ROB, then this was reported. Otherwise, if studies had mixed results for ‘low’ or ‘unclear or high risk of bias’, then the outcome reported was ‘unclear or high risk of bias’.

**Abbreviations:** DXM, dexamethasone; IVTA, intravitreal triamcinolone acetonide; PDT, photodynamic therapy; RoB, risk of bias

# eTable 6: All Network Meta-Analyses Results

| **Treatment Comparison** | **NMA Estimate**  **(95% CrI) (95% PrI)** |
| --- | --- |
| **Proportion of patients experiencing VISION GAIN of ≥15 ETDRS letters**  34 RCTs, 8809 patients, 12 treatments + placebo  No inconsistency was observed in the overall NMA (Chi-square=1.79, p=0.41)  Between-study variance: 0.02 (0.00-0.14) | |
| aflibercept vs placebo | 8.47 [(4.72-15.77) (4.16-17.38)]* |
| bevacizumab vs placebo | 8.08 [(4.35-15.25) (3.82-16.75)]* |
| bevacizumab vs aflibercept | 0.96 [(0.64-1.39) (0.54-1.62)] |
| ranibizumab vs placebo | 9.18 [(5.22-16.71) (4.53-18.21)]* |
| ranibizumab vs aflibercept | 1.09 [(0.78-1.47) (0.65-1.76)] |
| ranibizumab vs bevacizumab | 1.14 [(0.9-1.43) (0.73-1.8)] |
| PDT vs placebo | 2.23 [(1.27-4.11) (1.13-4.58)]* |
| PDT vs aflibercept | 0.26 [(0.15-0.49) (0.13-0.55)]* |
| PDT vs bevacizumab | 0.28 [(0.14-0.56) (0.13-0.63)]* |
| PDT vs ranibizumab | 0.24 [(0.13-0.47) (0.12-0.53)]* |
| bevacizumab+PDT vs placebo | 7.16 [(2.94-16.89) (2.67-17.9)]* |
| bevacizumab+PDT vs aflibercept | 0.84 [(0.4-1.73) (0.37-1.91)] |
| bevacizumab+PDT vs bevacizumab | 0.88 [(0.48-1.63) (0.43-1.79)] |
| bevacizumab+PDT vs ranibizumab | 0.77 [(0.4-1.5) (0.36-1.65)] |
| bevacizumab+PDT vs PDT | 3.19 [(1.27-7.78) (1.15-8.27)]* |
| PDT+ranibizumab vs placebo | 5.87 [(2.78-12.36) (2.51-13.5)]* |
| PDT+ranibizumab vs aflibercept | 0.69 [(0.39-1.21) (0.34-1.35)] |
| PDT+ranibizumab vs bevacizumab | 0.72 [(0.43-1.22) (0.38-1.39)] |
| PDT+ranibizumab vs ranibizumab | 0.64 [(0.4-1.01) (0.35-1.16)] |
| PDT+ranibizumab vs PDT | 2.63 [(1.16-5.63) (1.05-6.01)]* |
| PDT+ranibizumab vs bevacizumab+PDT | 0.82 [(0.37-1.86) (0.33-2.01)] |
| IVTA+PDT vs placebo | 14.04 [(1.66-541.8) (1.61-540.6)]* |
| IVTA+PDT vs aflibercept | 1.66 [(0.19-63.41) (0.19-64.5)] |
| IVTA+PDT vs bevacizumab | 1.75 [(0.2-66.18) (0.2-69.46)] |
| IVTA+PDT vs ranibizumab | 1.54 [(0.18-58.01) (0.18-58.2)] |
| IVTA+PDT vs PDT | 6.26 [(0.81-220.6) (0.78-225.4)] |
| IVTA+PDT vs bevacizumab+PDT | 2.02 [(0.21-79.48) (0.2-79.93)] |
| IVTA+PDT vs PDT+ranibizumab | 2.4 [(0.27-94.57) (0.26-97.47)] |
| DXM+ranibizumab vs placebo | 9.09 [(3.28-25.77) (3.09-27.7)]* |
| DXM+ranibizumab vs aflibercept | 1.07 [(0.44-2.68) (0.41-2.87)] |
| DXM+ranibizumab vs bevacizumab | 1.12 [(0.47-2.74) (0.44-3)] |
| DXM+ranibizumab vs ranibizumab | 0.99 [(0.42-2.34) (0.39-2.53)] |
| DXM+ranibizumab vs PDT | 4.07 [(1.39-11.69) (1.31-12.21)]* |
| DXM+ranibizumab vs bevacizumab+PDT | 1.28 [(0.44-3.76) (0.41-3.99)] |
| DXM+ranibizumab vs PDT+ranibizumab | 1.56 [(0.6-4.19) (0.56-4.48)] |
| DXM+ranibizumab vs IVTA+PDT | 0.63 [(0.02-6.92) (0.02-7.03)] |
| DXM+PDT+ranibizumab vs placebo | 4.83 [(1.29-16.46) (1.24-17.37)]* |
| DXM+PDT+ranibizumab vs aflibercept | 0.57 [(0.17-1.73) (0.16-1.82)] |
| DXM+PDT+ranibizumab vs bevacizumab | 0.6 [(0.18-1.74) (0.17-1.86)] |
| DXM+PDT+ranibizumab vs ranibizumab | 0.53 [(0.16-1.51) (0.15-1.6)] |
| DXM+PDT+ranibizumab vs PDT | 2.16 [(0.56-7.39) (0.53-7.76)] |
| DXM+PDT+ranibizumab vs bevacizumab+PDT | 0.68 [(0.17-2.36) (0.17-2.47)] |
| DXM+PDT+ranibizumab vs PDT+ranibizumab | 0.83 [(0.25-2.42) (0.24-2.55)] |
| DXM+PDT+ranibizumab vs IVTA+PDT | 0.33 [(0.01-3.89) (0.01-3.98)] |
| DXM+PDT+ranibizumab vs DXM+ranibizumab | 0.53 [(0.13-2.03) (0.12-2.13)] |
| bevacizumab+IVTA vs placebo | 19.64 [(6.8-55.9) (6.29-59.65)]* |
| bevacizumab+IVTA vs aflibercept | 2.3 [(0.9-5.94) (0.84-6.25)] |
| bevacizumab+IVTA vs bevacizumab | 2.42 [(1.04-5.72) (0.95-6.21)]* |
| bevacizumab+IVTA vs ranibizumab | 2.12 [(0.88-5.22) (0.83-5.58)] |
| bevacizumab+IVTA vs PDT | 8.77 [(2.92-25.85) (2.74-26.89)]* |
| bevacizumab+IVTA vs bevacizumab+PDT | 2.77 [(0.95-7.94) (0.9-8.4)] |
| bevacizumab+IVTA vs PDT+ranibizumab | 3.35 [(1.21-9.34) (1.15-9.94)]* |
| bevacizumab+IVTA vs IVTA+PDT | 1.36 [(0.03-14.13) (0.04-14.23)] |
| bevacizumab+IVTA vs DXM+ranibizumab | 2.15 [(0.63-7.25) (0.59-7.74)] |
| bevacizumab+IVTA vs DXM+PDT+ranibizumab | 4.07 [(1.01-17.9) (0.97-18.67)]* |
| brolucizumab vs placebo | 10.19 [(5.11-20.71) (4.6-22.48)]* |
| brolucizumab vs aflibercept | 1.2 [(0.85-1.71) (0.71-2.03)] |
| brolucizumab vs bevacizumab | 1.26 [(0.76-2.14) (0.67-2.44)] |
| brolucizumab vs ranibizumab | 1.11 [(0.71-1.8) (0.61-2.07)] |
| brolucizumab vs PDT | 4.58 [(2.25-9.07) (2.03-9.83)]* |
| brolucizumab vs bevacizumab+PDT | 1.43 [(0.65-3.23) (0.61-3.52)] |
| brolucizumab vs PDT+ranibizumab | 1.74 [(0.9-3.44) (0.81-3.78)] |
| brolucizumab vs IVTA+PDT | 0.72 [(0.02-6.33) (0.02-6.51)] |
| brolucizumab vs DXM+ranibizumab | 1.13 [(0.43-2.95) (0.39-3.09)] |
| brolucizumab vs DXM+PDT+ranibizumab | 2.11 [(0.67-7.56) (0.64-8.07)] |
| brolucizumab vs bevacizumab+IVTA | 0.52 [(0.19-1.44) (0.18-1.55)] |
| conbercept vs placebo | 1.58 [(0.57-4.71) (0.54-5.12)] |
| conbercept vs aflibercept | 0.19 [(0.06-0.65) (0.05-0.68)]* |
| conbercept vs bevacizumab | 0.2 [(0.06-0.69) (0.06-0.73)]* |
| conbercept vs ranibizumab | 0.17 [(0.05-0.59) (0.05-0.63)]* |
| conbercept vs PDT | 0.71 [(0.22-2.44) (0.21-2.6)] |
| conbercept vs bevacizumab+PDT | 0.22 [(0.06-0.88) (0.06-0.92)]* |
| conbercept vs PDT+ranibizumab | 0.27 [(0.08-1.02) (0.07-1.07)] |
| conbercept vs IVTA+PDT | 0.11 [(0-1.24) (0-1.26)] |
| conbercept vs DXM+ranibizumab | 0.18 [(0.04-0.76) (0.04-0.8)]* |
| conbercept vs DXM+PDT+ranibizumab | 0.33 [(0.07-1.83) (0.07-1.94)] |
| conbercept vs bevacizumab+IVTA | 0.08 [(0.02-0.38) (0.02-0.4)]* |
| conbercept vs brolucizumab | 0.15 [(0.05-0.56) (0.04-0.59)]* |
| **Proportion of patients experiencing VISION LOSS of ≥15 ETDRS letters**  36 RCTs, 9081 patients, 13 treatments + placebo  No inconsistency was observed in the overall NMA (Chi-square=0.25, p=0.88)  Between-study variance: 0.02 (0.00-0.13) | |
| aflibercept vs placebo | 0.13 [(0.07-0.25) (0.07-0.27)]* |
| bevacizumab vs placebo | 0.13 [(0.07-0.22) (0.06-0.24)]* |
| bevacizumab vs aflibercept | 0.94 [(0.51-1.67) (0.47-1.81)] |
| ranibizumab vs placebo | 0.12 [(0.07-0.19) (0.07-0.21)]* |
| ranibizumab vs aflibercept | 0.9 [(0.55-1.43) (0.5-1.59)] |
| ranibizumab vs bevacizumab | 0.96 [(0.69-1.35) (0.6-1.57)] |
| PDT vs placebo | 0.61 [(0.46-0.82) (0.39-0.96)]* |
| PDT vs aflibercept | 4.53 [(2.38-8.76) (2.18-9.5)]* |
| PDT vs bevacizumab | 4.84 [(2.62-9.29) (2.39-10.08)]* |
| PDT vs ranibizumab | 5.05 [(3-8.75) (2.73-9.7)] * |
| bevacizumab+PDT vs placebo | 0.14 [(0.05-0.39) (0.04-0.41)] * |
| bevacizumab+PDT vs aflibercept | 1.02 [(0.35-2.91) (0.34-3.06)] |
| bevacizumab+PDT vs bevacizumab | 1.09 [(0.46-2.62) (0.43-2.79)] |
| bevacizumab+PDT vs ranibizumab | 1.14 [(0.44-2.93) (0.42-3.12)] |
| bevacizumab+PDT vs PDT | 0.22 [(0.08-0.66) (0.07-0.71)] * |
| PDT+ranibizumab vs placebo | 0.17 [(0.07-0.39) (0.06-0.41)] * |
| PDT+ranibizumab vs aflibercept | 1.23 [(0.51-2.94) (0.48-3.11)] |
| PDT+ranibizumab vs bevacizumab | 1.33 [(0.59-2.95) (0.55-3.18)] |
| PDT+ranibizumab vs ranibizumab | 1.38 [(0.66-2.86) (0.61-3.08)] |
| PDT+ranibizumab vs PDT | 0.27 [(0.11-0.66) (0.1-0.7)] * |
| PDT+ranibizumab vs bevacizumab+PDT | 1.21 [(0.37-3.91) (0.35-4.1)] |
| IVTA+PDT vs placebo | 0.29 [(0.09-0.89) (0.09-0.93)] * |
| IVTA+PDT vs aflibercept | 2.18 [(0.58-7.72) (0.56-8.16)] |
| IVTA+PDT vs bevacizumab | 2.34 [(0.64-8.08) (0.61-8.4)] |
| IVTA+PDT vs ranibizumab | 2.45 [(0.69-8.09) (0.65-8.45)] |
| IVTA+PDT vs PDT | 0.48 [(0.15-1.4) (0.15-1.48)] |
| IVTA+PDT vs bevacizumab+PDT | 2.15 [(0.44-9.94) (0.42-10.24)] |
| IVTA+PDT vs PDT+ranibizumab | 1.78 [(0.41-7.21) (0.39-7.49)] |
| IVTA vs placebo | 0.91 [(0.42-1.98) (0.39-2.12)] |
| IVTA vs aflibercept | 6.8 [(2.47-18.51) (2.35-19.44)] * |
| IVTA vs bevacizumab | 7.27 [(2.78-19.46) (2.63-20.61)] * |
| IVTA vs ranibizumab | 7.56 [(3.04-19.18) (2.89-20.29)] * |
| IVTA vs PDT | 1.49 [(0.65-3.46) (0.61-3.7)] |
| IVTA vs bevacizumab+PDT | 6.67 [(1.79-24.88) (1.72-25.71)] * |
| IVTA vs PDT+ranibizumab | 5.52 [(1.73-17.77) (1.65-18.68)] * |
| IVTA vs IVTA+PDT | 3.11 [(0.8-12.72) (0.76-13.23)] |
| DXM+ranibizumab vs placebo | 0.05 [(0.01-0.32) (0.01-0.33)] * |
| DXM+ranibizumab vs aflibercept | 0.39 [(0.04-2.45) (0.04-2.51)] |
| DXM+ranibizumab vs bevacizumab | 0.42 [(0.05-2.47) (0.05-2.52)] |
| DXM+ranibizumab vs ranibizumab | 0.43 [(0.05-2.54) (0.05-2.65)] |
| DXM+ranibizumab vs PDT | 0.09 [(0.01-0.54) (0.01-0.55)] * |
| DXM+ranibizumab vs bevacizumab+PDT | 0.38 [(0.04-2.75) (0.04-2.81)] |
| DXM+ranibizumab vs PDT+ranibizumab | 0.31 [(0.03-2.14) (0.03-2.18)] |
| DXM+ranibizumab vs IVTA+PDT | 0.18 [(0.02-1.54) (0.02-1.57)] |
| DXM+ranibizumab vs IVTA | 0.06 [(0.01-0.42) (0.01-0.43)] * |
| DXM+PDT+ranibizumab vs placebo | 0.12 [(0.03-0.39) (0.03-0.41)] * |
| DXM+PDT+ranibizumab vs aflibercept | 0.86 [(0.23-2.98) (0.22-3.13)] |
| DXM+PDT+ranibizumab vs bevacizumab | 0.93 [(0.26-3.04) (0.25-3.2)] |
| DXM+PDT+ranibizumab vs ranibizumab | 0.97 [(0.28-3.03) (0.27-3.19)] |
| DXM+PDT+ranibizumab vs PDT | 0.19 [(0.05-0.66) (0.05-0.7)] * |
| DXM+PDT+ranibizumab vs bevacizumab+PDT | 0.84 [(0.18-3.79) (0.18-3.94)] |
| DXM+PDT+ranibizumab vs PDT+ranibizumab | 0.7 [(0.21-2.13) (0.2-2.24)] |
| DXM+PDT+ranibizumab vs IVTA+PDT | 0.39 [(0.07-2.16) (0.07-2.23)] |
| DXM+PDT+ranibizumab vs IVTA | 0.13 [(0.03-0.55) (0.03-0.58)] * |
| DXM+PDT+ranibizumab vs DXM+ranibizumab | 2.26 [(0.26-24.14) (0.25-24.68)] |
| bevacizumab+IVTA vs placebo | 0.14 [(0-99.31) (0-100.3)] |
| bevacizumab+IVTA vs aflibercept | 1.03 [(0-756.6) (0-772.3)] |
| bevacizumab+IVTA vs bevacizumab | 1.09 [(0-799.1) (0-778)] |
| bevacizumab+IVTA vs ranibizumab | 1.15 [(0-837.5) (0-848.7)] |
| bevacizumab+IVTA vs PDT | 0.23 [(0-169) (0-169.3)] |
| bevacizumab+IVTA vs bevacizumab+PDT | 1.01 [(0-734.3) (0-757.5)] |
| bevacizumab+IVTA vs PDT+ranibizumab | 0.84 [(0-633.6) (0-634.7)] |
| bevacizumab+IVTA vs IVTA+PDT | 0.48 [(0-377.9) (0-370.4)] |
| bevacizumab+IVTA vs IVTA | 0.15 [(0-118) (0-120.3)] |
| bevacizumab+IVTA vs DXM+ranibizumab | 2.72 [(0-2543) (0-2671)] |
| bevacizumab+IVTA vs DXM+PDT+ranibizumab | 1.2 [(0-864.3) (0-879.5)] |
| brolucizumab vs placebo | 0.13 [(0.06-0.29) (0.05-0.31)] * |
| brolucizumab vs aflibercept | 0.96 [(0.57-1.63) (0.51-1.79)] |
| brolucizumab vs bevacizumab | 1.03 [(0.47-2.27) (0.44-2.43)] |
| brolucizumab vs ranibizumab | 1.08 [(0.53-2.19) (0.49-2.36)] |
| brolucizumab vs PDT | 0.21 [(0.09-0.48) (0.08-0.52)] * |
| brolucizumab vs bevacizumab+PDT | 0.95 [(0.29-3.08) (0.28-3.23)] |
| brolucizumab vs PDT+ranibizumab | 0.78 [(0.29-2.17) (0.27-2.3)] |
| brolucizumab vs IVTA+PDT | 0.44 [(0.11-1.81) (0.11-1.91)] |
| brolucizumab vs IVTA | 0.14 [(0.05-0.45) (0.04-0.47)] * |
| brolucizumab vs DXM+ranibizumab | 2.49 [(0.37-23.67) (0.36-23.89)] |
| brolucizumab vs DXM+PDT+ranibizumab | 1.12 [(0.29-4.63) (0.28-4.8)] |
| brolucizumab vs bevacizumab+IVTA | 0.94 [(0-602.8) (0-610.9)] |
| conbercept vs placebo | 0.03 [(0-0.53) (0-0.55)] * |
| conbercept vs aflibercept | 0.24 [(0-4.29) (0-4.4)] |
| conbercept vs bevacizumab | 0.26 [(0-4.65) (0-4.67)] |
| conbercept vs ranibizumab | 0.27 [(0-4.67) (0-4.79)] |
| conbercept vs PDT | 0.05 [(0-0.89) (0-0.92)] * |
| conbercept vs bevacizumab+PDT | 0.23 [(0-4.85) (0-4.97)] |
| conbercept vs PDT+ranibizumab | 0.19 [(0-3.84) (0-3.89)] |
| conbercept vs IVTA+PDT | 0.11 [(0-2.39) (0-2.44)] |
| conbercept vs IVTA | 0.03 [(0-0.66) (0-0.67)] * |
| conbercept vs DXM+ranibizumab | 0.59 [(0-21.31) (0-21.85)] |
| conbercept vs DXM+PDT+ranibizumab | 0.26 [(0-6.3) (0-6.38)] |
| conbercept vs bevacizumab+IVTA | 0.16 [(0-231.1) (0-232.8)] |
| conbercept vs brolucizumab | 0.24 [(0-4.71) (0-4.85)] |
| **Mortality**  24 RCTs, 10 treatments + placebo, 8875 patients  No inconsistency in the network (chi-squared=0.69, p-value=0.71)  Between study variance: 0.01 (0.00-0.17) | |
| aflibercept vs placebo | 1.58 [(0.37-7.86) (0.35-8.2)] |
| bevacizumab vs placebo | 0.92 [(0.26-3.31) (0.25-3.46)] |
| bevacizumab vs aflibercept | 0.58 [(0.15-1.98) (0.15-2.09)] |
| ranibizumab vs placebo | 0.94 [(0.29-3) (0.27-3.17)] |
| ranibizumab vs aflibercept | 0.59 [(0.17-1.8) (0.16-1.9)] |
| ranibizumab vs bevacizumab | 1.02 [(0.6-1.73) (0.54-1.94)] |
| PDT vs placebo | 1.2 [(0.59-2.47) (0.55-2.68)] |
| PDT vs aflibercept | 0.76 [(0.14-3.34) (0.14-3.57)] |
| PDT vs bevacizumab | 1.3 [(0.32-5.31) (0.31-5.58)] |
| PDT vs ranibizumab | 1.28 [(0.35-4.68) (0.34-4.84)] |
| bevacizumab+PDT vs placebo | 6.46 [(0.14-1474) (0.14-1474)] |
| bevacizumab+PDT vs aflibercept | 3.77 [(0.09-943.9) (0.08-944.1)] |
| bevacizumab+PDT vs bevacizumab | 6.49 [(0.19-1610) (0.19-1546)] |
| bevacizumab+PDT vs ranibizumab | 6.46 [(0.18-1444) (0.18-1486)] |
| bevacizumab+PDT vs PDT | 5.39 [(0.11-1183) (0.11-1196)] |
| PDT+ranibizumab vs placebo | 0.51 [(0.1-2.6) (0.1-2.68)] |
| PDT+ranibizumab vs aflibercept | 0.32 [(0.06-1.57) (0.06-1.61)] |
| PDT+ranibizumab vs bevacizumab | 0.57 [(0.15-1.84) (0.14-1.92)] |
| PDT+ranibizumab vs ranibizumab | 0.55 [(0.16-1.6) (0.16-1.7)] |
| PDT+ranibizumab vs PDT | 0.42 [(0.08-2.42) (0.07-2.48)] |
| PDT+ranibizumab vs bevacizumab+PDT | 0.08 [(0-3.6) (0-3.72)] |
| IVTA+PDT vs placebo | 0.11 [(0-2.63) (0-2.68)] |
| IVTA+PDT vs aflibercept | 0.07 [(0-2.26) (0-2.29)] |
| IVTA+PDT vs bevacizumab | 0.12 [(0-3.77) (0-3.76)] |
| IVTA+PDT vs ranibizumab | 0.11 [(0-3.47) (0-3.54)] |
| IVTA+PDT vs PDT | 0.09 [(0-1.9) (0-1.93)] |
| IVTA+PDT vs bevacizumab+PDT | 0.01 [(0-2.66) (0-2.68)] |
| IVTA+PDT vs PDT+ranibizumab | 0.21 [(0-7.26) (0-7.4)] |
| IVTA vs placebo | 1.84 [(0.39-9.24) (0.38-9.67)] |
| IVTA vs aflibercept | 1.17 [(0.13-9.79) (0.13-10.08)] |
| IVTA vs bevacizumab | 2.02 [(0.27-14.87) (0.27-15.33)] |
| IVTA vs ranibizumab | 2 [(0.3-13.54) (0.29-13.91)] |
| IVTA vs PDT | 1.56 [(0.27-8.77) (0.26-9)] |
| IVTA vs bevacizumab+PDT | 0.29 [(0-19.01) (0-19.16)] |
| IVTA vs PDT+ranibizumab | 3.68 [(0.39-33.01) (0.37-34.64)] |
| IVTA vs IVTA+PDT | 17.91 [(0.47-8338) (0.47-8334)] |
| DXM+PDT+ranibizumab vs placebo | 0.61 [(0.08-4.45) (0.07-4.66)] |
| DXM+PDT+ranibizumab vs aflibercept | 0.38 [(0.05-2.56) (0.04-2.63)] |
| DXM+PDT+ranibizumab vs bevacizumab | 0.66 [(0.11-3.37) (0.11-3.45)] |
| DXM+PDT+ranibizumab vs ranibizumab | 0.66 [(0.11-2.95) (0.11-3.1)] |
| DXM+PDT+ranibizumab vs PDT | 0.51 [(0.06-3.87) (0.06-4.01)] |
| DXM+PDT+ranibizumab vs bevacizumab+PDT | 0.09 [(0-5.43) (0-5.48)] |
| DXM+PDT+ranibizumab vs PDT+ranibizumab | 1.2 [(0.2-6.1) (0.19-6.41)] |
| DXM+PDT+ranibizumab vs IVTA+PDT | 5.93 [(0.12-2536) (0.12-2558)] |
| DXM+PDT+ranibizumab vs IVTA | 0.33 [(0.02-4) (0.02-2558)] |
| brolucizumab vs placebo | 1.1 [(0.18-7.51) (0.17-2558)] |
| brolucizumab vs aflibercept | 0.7 [(0.24-1.91) (0.23-2558)] |
| brolucizumab vs bevacizumab | 1.21 [(0.24-6.49) (0.23-2558)] |
| brolucizumab vs ranibizumab | 1.19 [(0.25-5.98) (0.24-2558)] |
| brolucizumab vs PDT | 0.92 [(0.15-6.67) (0.14-2558)] |
| brolucizumab vs bevacizumab+PDT | 0.18 [(0-10.12) (0-2558)] |
| brolucizumab vs PDT+ranibizumab | 2.22 [(0.31-15.25) (0.3-2558)] |
| brolucizumab vs IVTA+PDT | 10.87 [(0.27-4065) (0.26-2558)] |
| brolucizumab vs IVTA | 0.59 [(0.06-6.86) (0.05-2558)] |
| brolucizumab vs DXM+PDT+ranibizumab | 1.84 [(0.2-19.07) (0.19-2558)] |
| **Difference in Mean Change in BCVA**  26 RCTs, 10 treatments + placebo, 5916 patients  No inconsistency in the network (chi-squared=2.62, p-value=0.27)  Between-study variance: 6.29 (3.28-11.27) | |
| aflibercept vs placebo | 16.34 [(11.69 to 21.03) (9.49 to 23.27)] * |
| bevacizumab vs placebo | 18.56 [(13.74 to 23.27) (11.59 to 25.36)] * |
| bevacizumab vs aflibercept | 2.21 [(-1.1 to 5.42) (-3.96 to 8.22)] |
| ranibizumab vs placebo | 17.43 [(13.09 to 21.78) (10.8 to 24.16)] * |
| ranibizumab vs aflibercept | 1.09 [(-1.53 to 3.7) (-4.62 to 6.81)] |
| ranibizumab vs bevacizumab | -1.11 [(-3.07 to 0.92) (-6.5 to 4.28)] |
| PDT vs placebo | 6.39 [(1.54 to 11.2) (-0.64 to 13.34)] * |
| PDT vs aflibercept | -9.98 [(-14.81 to -5.18) (-16.98 to -2.98)] * |
| PDT vs bevacizumab | -12.21 [(-17.54 to -6.75)(-19.61 to -4.82)] * |
| PDT vs ranibizumab | -11.07 [(-16.12 to -5.98)(-18.19 to -3.84)] * |
| bevacizumab+PDT vs placebo | 16.14 [(9.86 to 22.24) (8.03 to 24.11)] * |
| bevacizumab+PDT vs aflibercept | -0.19 [(-5.49 to 5.02) (-7.63 to 7.01)] |
| bevacizumab+PDT vs bevacizumab | -2.42 [(-6.42 to 1.52) (-8.85 to 4.06)] |
| bevacizumab+PDT vs ranibizumab | -1.3 [(-5.83 to 3.12) (-8.13 to 5.39)] |
| bevacizumab+PDT vs PDT | 9.78 [(2.88 to 16.53) (1.26 to 18.25)] * |
| PDT+ranibizumab vs placebo | 12.92 [(7.84 to 17.87) (5.75 to 19.92)] * |
| PDT+ranibizumab vs aflibercept | -3.45 [(-7.09 to 0.14) (-9.81 to 2.68)] |
| PDT+ranibizumab vs bevacizumab | -5.63 [(-8.86 to -2.49) (-11.7 to 0.3)] * |
| PDT+ranibizumab vs ranibizumab | -4.52 [(-7.07 to -2.07) (-10.21 to 0.88)] |
| PDT+ranibizumab vs PDT | 6.56 [(0.86 to 12.11) (-1.02 to 13.89)] * |
| PDT+ranibizumab vs bevacizumab+PDT | -3.24 [(-8.35 to 1.85) (-10.39 to 3.87)] |
| IVTA+PDT vs placebo | 9.43 [(2.47 to 16.38) (0.77 to 18.05)] * |
| IVTA+PDT vs aflibercept | -6.93 [(-13.96 to 0.17) (-15.29 to 1.82)] |
| IVTA+PDT vs bevacizumab | -9.12 [(-16.46 to -1.68) (-18.06 to -0.02)] * |
| IVTA+PDT vs ranibizumab | -8.03 [(-15.11 to -0.78) (-16.78 to 0.83)] * |
| IVTA+PDT vs PDT | 3.03 [(-2.03 to 8.15) (-4.15 to 10.22)] |
| IVTA+PDT vs bevacizumab+PDT | -6.7 [(-15 to 1.88) (-16.42 to 3.36)] |
| IVTA+PDT vs PDT+ranibizumab | -3.49 [(-10.93 to 4.27) (-12.44 to 5.74)] |
| DXM+ranibizumab vs placebo | 16.14 [(9.68 to 22.33) (7.92 to 24.11)] * |
| DXM+ranibizumab vs aflibercept | -0.21 [(-5.54 to 4.89) (-7.54 to 6.94)] |
| DXM+ranibizumab vs bevacizumab | -2.41 [(-7.43 to 2.35) (-9.57 to 4.71)] |
| DXM+ranibizumab vs ranibizumab | -1.31 [(-5.94 to 3.08) (-8.23 to 5.37)] |
| DXM+ranibizumab vs PDT | 9.75 [(2.93 to 16.33) (1.32 to 17.98)] * |
| DXM+ranibizumab vs bevacizumab+PDT | -0.02 [(-6.38 to 6.35) (-8.21 to 8.07)] |
| DXM+ranibizumab vs PDT+ranibizumab | 3.2 [(-1.9 to 8.28) (-3.99 to 10.47)] |
| DXM+ranibizumab vs IVTA+PDT | 6.71 [(-1.86 to 15.04) (-3.33 to 16.45)] |
| DXM+PDT+ranibizumab vs placebo | 14.15 [(4.75 to 23.31) (3.52 to 24.53)] * |
| DXM+PDT+ranibizumab vs aflibercept | -2.2 [(-10.81 to 6.25) (-11.99 to 7.66)] |
| DXM+PDT+ranibizumab vs bevacizumab | -4.43 [(-12.69 to 3.91) (-14.28 to 5.32)] |
| DXM+PDT+ranibizumab vs ranibizumab | -3.29 [(-11.48 to 4.75) (-12.94 to 6.23)] |
| DXM+PDT+ranibizumab vs PDT | 7.74 [(-1.92 to 17.26) (-3.1 to 18.45)] |
| DXM+PDT+ranibizumab vs bevacizumab+PDT | -2.02 [(-11.23 to 7.19) (-12.49 to 8.54)] |
| DXM+PDT+ranibizumab vs PDT+ranibizumab | 1.2 [(-6.87 to 9.34) (-8.37 to 10.88)] |
| DXM+PDT+ranibizumab vs IVTA+PDT | 4.74 [(-6.27 to 15.53) (-7.31 to 16.5)] |
| DXM+PDT+ranibizumab vs DXM+ranibizumab | -1.96 [(-11.27 to 7.23) (-12.48 to 8.62)] |
| bevacizumab+IVTA vs placebo | 21.95 [(15.56 to 28.24) (13.7 to 30.06)] * |
| bevacizumab+IVTA vs aflibercept | 5.57 [(0.2 to 10.93) (-1.81 to 12.86)] * |
| bevacizumab+IVTA vs bevacizumab | 3.38 [(-0.82 to 7.59) (-3.25 to 10.11)] |
| bevacizumab+IVTA vs ranibizumab | 4.49 [(-0.17 to 9.16) (-2.39 to 11.43)] |
| bevacizumab+IVTA vs PDT | 15.55 [(8.68 to 22.38) (6.97 to 23.88)] * |
| bevacizumab+IVTA vs bevacizumab+PDT | 5.76 [(0.08 to 11.71) (-1.89 to 13.58)] * |
| bevacizumab+IVTA vs PDT+ranibizumab | 9 [(3.8 to 14.43) (1.87 to 16.38)] * |
| bevacizumab+IVTA vs IVTA+PDT | 12.53 [(4.02 to 20.99) (2.66 to 22.24)] * |
| bevacizumab+IVTA vs DXM+ranibizumab | 5.78 [(-0.57 to 12.35) (-2.27 to 14.14)] |
| bevacizumab+IVTA vs DXM+PDT+ranibizumab | 7.77 [(-1.38 to 17.12) (-2.64 to 18.44)] |
| bevacizumab+IVTA+PDT vs placebo | 19.26 [(6.9 to 31.35) (5.9 to 32.5)] * |
| bevacizumab+IVTA+PDT vs aflibercept | 2.87 [(-9.05 to 14.74) (-9.85 to 15.94)] |
| bevacizumab+IVTA+PDT vs bevacizumab | 0.71 [(-10.75 to 12.06) (-11.63 to 13.02)] |
| bevacizumab+IVTA+PDT vs ranibizumab | 1.83 [(-9.8 to 13.39) (-10.68 to 14.18)] |
| bevacizumab+IVTA+PDT vs PDT | 12.84 [(0.32 to 25.5) (-0.61 to 26.51)] * |
| bevacizumab+IVTA+PDT vs bevacizumab+PDT | 3.12 [(-7.56 to 13.76) (-8.49 to 14.88)] |
| bevacizumab+IVTA+PDT vs PDT+ranibizumab | 6.4 [(-5.53 to 18.22) (-6.42 to 19.29)] |
| bevacizumab+IVTA+PDT vs IVTA+PDT | 9.83 [(-3.73 to 23.34) (-4.87 to 24.28)] |
| bevacizumab+IVTA+PDT vs DXM+ranibizumab | 3.14 [(-9.34 to 15.47) (-10.27 to 16.51)] |
| bevacizumab+IVTA+PDT vs DXM+PDT+ranibizumab | 5.09 [(-8.8 to 19.22) (-9.63 to 20.1)] |
| bevacizumab+IVTA+PDT vs bevacizumab+IVTA | -2.7 [(-14.76 to 9.47) (-15.8 to 10.45)] |
| brolucizumab vs placebo | 15.88 [(9.84 to 21.94) (7.99 to 23.89)] * |
| brolucizumab vs aflibercept | -0.46 [(-4.26 to 3.33) (-6.84 to 5.81)] |
| brolucizumab vs bevacizumab | -2.68 [(-7.69 to 2.43) (-9.72 to 4.54)] |
| brolucizumab vs ranibizumab | -1.57 [(-6.12 to 3.07) (-8.34 to 5.32)] |
| brolucizumab vs PDT | 9.53 [(3.39 to 15.58) (1.52 to 17.48)] * |
| brolucizumab vs bevacizumab+PDT | -0.26 [(-6.66 to 6.18) (-8.3 to 7.9)] |
| brolucizumab vs PDT+ranibizumab | 2.97 [(-2.17 to 8.31) (-4.29 to 10.34)] |
| brolucizumab vs IVTA+PDT | 6.47 [(-1.6 to 14.47) (-2.97 to 16.01)] |
| brolucizumab vs DXM+ranibizumab | -0.24 [(-6.53 to 6.35) (-8.25 to 8.05)] |
| brolucizumab vs DXM+PDT+ranibizumab | 1.72 [(-7.42 to 11.2) (-8.81 to 12.57)] |
| brolucizumab vs bevacizumab+IVTA | -6.04 [(-12.56 to 0.57) (-14.28 to 2.22)] |
| brolucizumab vs bevacizumab+IVTA+PDT | -3.4 [(-15.88 to 9.23) (-16.77 to 10.29)] |
| conbercept vs placebo | 1.2 [(-6.08 to 8.62) (-7.6 to 10.06)] |
| conbercept vs aflibercept | -15.17 [(-23.8 to -6.5) (-25.35 to -4.89)] * |
| conbercept vs bevacizumab | -17.35 [(-25.84 to -8.57) (-27.14 to -7.16)] * |
| conbercept vs ranibizumab | -16.23 [(-24.57 to -7.74) (-25.97 to -6.25)] * |
| conbercept vs PDT | -5.17 [(-13.84 to 3.64) (-15.14 to 4.97)] |
| conbercept vs bevacizumab+PDT | -14.95 [(-24.37 to -5.16) (-25.72 to -4.04)] * |
| conbercept vs PDT+ranibizumab | -11.7 [(-20.38 to -2.86) (-21.92 to -1.39)] * |
| conbercept vs IVTA+PDT | -8.27 [(-18.39 to 1.81) (-19.42 to 3.01)] |
| conbercept vs DXM+ranibizumab | -14.93 [(-24.43 to -5.28) (-25.66 to -3.91)] * |
| conbercept vs DXM+PDT+ranibizumab | -12.86 [(-24.46 to -1.13) (-25.63 to -0.02)] * |
| conbercept vs bevacizumab+IVTA | -20.75 [(-30.18 to -11.07) (-31.5 to -9.69)] * |
| conbercept vs bevacizumab+IVTA+PDT | -18.04 [(-32.13 to -3.8) (-33.06 to -2.9)] * |
| conbercept vs brolucizumab | -14.68 [(-24.01 to -5.17) (-25.48 to -3.94)] * |
| **Adverse Events (AEs)**  15 RCTs, 8 treatments + placebo, 5785 patients  No inconsistency in the network (chi-squared=0.01, p-value=0.93)  Between-study variance: 0.01 (0.00-0.15) | |
| aflibercept vs placebo | 1.32 [(0.78-2.23) (0.72-2.45)] |
| bevacizumab vs placebo | 1.45 [(0.66-3.05) (0.61-3.22)] |
| bevacizumab vs aflibercept | 1.11 [(0.53-2.1) (0.49-2.25)] |
| ranibizumab vs placebo | 1.61 [(0.92-2.88) (0.83-3.13)] |
| ranibizumab vs aflibercept | 1.23 [(0.76-1.93) (0.67-2.16)] |
| ranibizumab vs bevacizumab | 1.11 [(0.71-1.87) (0.63-2.12)] |
| PDT vs placebo | 1.57 [(1.07-2.3) (0.94-2.6)] * |
| PDT vs aflibercept | 1.19 [(0.79-1.8) (0.68-2.04)] |
| PDT vs bevacizumab | 1.08 [(0.53-2.35) (0.5-2.51)] |
| PDT vs ranibizumab | 0.97 [(0.56-1.7) (0.52-1.89)] |
| IVTA vs placebo | 1.82 [(0.39-9.63) (0.38-9.89)] |
| IVTA vs aflibercept | 1.37 [(0.28-7.8) (0.26-8.11)] |
| IVTA vs bevacizumab | 1.25 [(0.23-7.94) (0.22-8.12)] |
| IVTA vs ranibizumab | 1.12 [(0.22-6.54) (0.22-6.74)] |
| IVTA vs PDT | 1.16 [(0.24-6.3) (0.23-6.52)] |
| DXM+ranibizumab vs placebo | 4.03 [(1.71-9.41) (1.61-9.95)] * |
| DXM+ranibizumab vs aflibercept | 3.04 [(1.4-6.65) (1.29-7.05)] * |
| DXM+ranibizumab vs bevacizumab | 2.76 [(1.3-6.31) (1.21-6.83)] * |
| DXM+ranibizumab vs ranibizumab | 2.48 [(1.35-4.73) (1.24-5.11)] * |
| DXM+ranibizumab vs PDT | 2.56 [(1.11-5.85) (1.02-6.23)] * |
| DXM+ranibizumab vs IVTA | 2.2 [(0.34-12.81) (0.33-13.02)] |
| brolucizumab vs placebo | 1.41 [(0.76-2.59) (0.69-2.81)] |
| brolucizumab vs aflibercept | 1.07 [(0.77-1.46) (0.67-1.69)] |
| brolucizumab vs bevacizumab | 0.97 [(0.48-2.14) (0.45-2.34)] |
| brolucizumab vs ranibizumab | 0.87 [(0.5-1.55) (0.46-1.72)] |
| brolucizumab vs PDT | 0.9 [(0.53-1.52) (0.48-1.69)] |
| brolucizumab vs IVTA | 0.77 [(0.13-4) (0.13-4.17)] |
| brolucizumab vs DXM+ranibizumab | 0.35 [(0.15-0.81) (0.14-0.88)] * |
| conbercept vs placebo | 0.98 [(0.42-2.24) (0.4-2.39)] |
| conbercept vs aflibercept | 0.74 [(0.28-2) (0.26-2.09)] |
| conbercept vs bevacizumab | 0.67 [(0.22-2.15) (0.21-2.3)] |
| conbercept vs ranibizumab | 0.61 [(0.22-1.68) (0.21-1.77)] |
| conbercept vs PDT | 0.62 [(0.25-1.57) (0.24-1.66)] |
| conbercept vs IVTA | 0.53 [(0.08-3.13) (0.08-3.27)] |
| conbercept vs DXM+ranibizumab | 0.24 [(0.08-0.82) (0.07-0.85)] * |
| conbercept vs brolucizumab | 0.69 [(0.25-1.96) (0.23-2.08)] |
| **Arterial thromboembolic events (ATE)**  15 RCTs, 8 treatments + placebo, 6365 patients  No source of inconsistency in the network (no closed loops)  Between-study variance: 0.03 (0.00-0.48) | |
| aflibercept vs placebo | 0.69 [(0.13-3.38) (0.12-3.66)] |
| bevacizumab vs placebo | 0.79 [(0.2-2.94) (0.18-3.24)] |
| bevacizumab vs aflibercept | 1.13 [(0.31-4.32) (0.29-4.78)] |
| ranibizumab vs placebo | 1.25 [(0.38-4.02) (0.33-4.58)] |
| ranibizumab vs aflibercept | 1.81 [(0.61-5.86) (0.54-6.68)] |
| ranibizumab vs bevacizumab | 1.6 [(0.85-3.15) (0.7-3.85)] |
| PDT vs placebo | 4.79 [(0.22-259.8) (0.21-273.7)] |
| PDT vs aflibercept | 6.94 [(0.54-293.6) (0.51-300.1)] |
| PDT vs bevacizumab | 6.14 [(0.36-291.7) (0.35-312)] |
| PDT vs ranibizumab | 3.8 [(0.24-180.6) (0.23-189.7)] |
| PDT+ranibizumab vs placebo | 0.72 [(0.11-4.56) (0.1-4.93)] |
| PDT+ranibizumab vs aflibercept | 1.06 [(0.16-6.66) (0.15-7.3)] |
| PDT+ranibizumab vs bevacizumab | 0.93 [(0.18-4.44) (0.17-4.91)] |
| PDT+ranibizumab vs ranibizumab | 0.59 [(0.13-2.37) (0.11-2.67)] |
| PDT+ranibizumab vs PDT | 0.15 [(0-3.43) (0-3.55)] |
| DXM+ranibizumab vs placebo | 0.51 [(0.01-8.68) (0.01-8.99)] |
| DXM+ranibizumab vs aflibercept | 0.75 [(0.02-12.44) (0.02-13.36)] |
| DXM+ranibizumab vs bevacizumab | 0.68 [(0.02-8.6) (0.02-9.19)] |
| DXM+ranibizumab vs ranibizumab | 0.42 [(0.01-5.07) (0.01-5.31)] |
| DXM+ranibizumab vs PDT | 0.1 [(0-4.33) (0-4.73)] |
| DXM+ranibizumab vs PDT+ranibizumab | 0.71 [(0.01-13.35) (0.01-14.28)] |
| brolucizumab vs placebo | 0.45 [(0.07-2.58) (0.07-2.8)] |
| brolucizumab vs aflibercept | 0.66 [(0.28-1.52) (0.24-1.82)] |
| brolucizumab vs bevacizumab | 0.58 [(0.12-2.61) (0.11-2.93)] |
| brolucizumab vs ranibizumab | 0.36 [(0.09-1.42) (0.08-1.57)] |
| brolucizumab vs PDT | 0.09 [(0-1.39) (0-1.44)] |
| brolucizumab vs PDT+ranibizumab | 0.62 [(0.08-4.86) (0.07-5.19)] |
| brolucizumab vs DXM+ranibizumab | 0.88 [(0.05-40.4) (0.04-43.09)] |
| conbercept vs placebo | 0.51 [(0.01-17.95) (0.01-18.96)] |
| conbercept vs aflibercept | 0.73 [(0.01-38.5) (0.01-39.9)] |
| conbercept vs bevacizumab | 0.66 [(0.01-31.63) (0.01-32.15)] |
| conbercept vs ranibizumab | 0.41 [(0.01-19.15) (0.01-20.03)] |
| conbercept vs PDT | 0.1 [(0-12.63) (0-12.9)] |
| conbercept vs PDT+ranibizumab | 0.7 [(0.01-41.86) (0.01-42.36)] |
| conbercept vs DXM+ranibizumab | 1.04 [(0.01-177.6) (0.01-186.4)] |
| conbercept vs brolucizumab | 1.1 [(0.02-62.85) (0.02-64.99)] |

*statistically significant difference

**Abbreviations:** DXM, dexamethasone; IVTA, intravitreal triamcinolone acetonide; PDT, photodynamic therapy

# eFigure 2: Comparison-adjusted Funnel Plots

## Vision Gain

## Vision Loss

## Mean Change in Best-corrected Visual Acuity

## Mortality

## Arterial Thromboembolic Events

## Adverse Events

We decided to order the treatments chronologically (based on when they appeared in the Canadian market) so that the comparison-adjusted funnel plot can be interpreted as suggested by Chaimani et al (2013). This plot accounts for the certain order the treatments are sorted as it also assesses the hypothesis whether newer treatments are favored over older ones. The pre-specified treatment order allows a valid interpretation of the direction of the centered overall mean effects.

# eTable 7: All Pairwise Meta-Analysis Results

| **Outcome** | **Comparison** | **# of RCTs** | **# of patients** | **Estimate type** | **Estimate [95% CrI]** |
| --- | --- | --- | --- | --- | --- |
| **Vision gain in BCVA of ≥15 ETDRS letters**  (Between-study variance:  OR, 0.03 [95% CrI, 0.00-0.15]) | conbercept vs placebo | 1 | 123 | OR | 1.56 (0.61-4.38) |
|  | ranibizumab vs placebo | 2 | 602 | OR | 7.52 (3.78-14.74)* |
|  | PDT vs placebo | 3 | 936 | OR | 2.88 (1.39-6.48)* |
|  | brolucizumab vs aflibercept | 2 |  | OR | 1.2 (0.84-1.72) |
|  | ranibizumab vs afilbercept | 3 | 1192 | OR | 1.13 (0.81-1.58) |
|  | ranibizumab vs bevacizumab | 9 | 2679 | OR | 1.13 (0.9-1.44) |
|  | PDT vs aflibercept | 1 |  | OR | 0.2 (0.1-0.38) |
|  | bevacizumab vs bevacizumab+IVTA | 1 |  | OR | 2.44 (1.13-5.39) |
|  | bevacizumab+PDT vs bevacizumab | 3 | 204 | OR | 0.88 (0.48-1.64) |
|  | DXM+PDT+ranibizumab vs ranibizumab | 1 | 80 | OR | 0.49 (0.15-1.45) |
|  | PDT+ranibizumab vs ranibizumab | 6 | 521 | OR | 0.64 (0.39-1.01) |
|  | DXM+ranibizumab vs ranibizumab | 2 | 279 | OR | 0.98 (0.42-2.34) |
|  | IVTA+PDT vs PDT | 1 | 57 | OR | 6.72 (0.87-208) |
|  | DXM+PDT+ranibizumab vs PDT+ranibizumab | 1 | 82 | OR | 0.93 (0.26-3.1) |
| **Vision loss in BCVA of ≥15 ETDRS letters**  (Between-study variance:  OR, 0.02 [95% CrI, 0.00-0.14]) | conbercept vs placeb | 1 | 123 | OR | 0.03 (0-0.62)* |
|  | ranibizumab vs placebo | 2 | 602 | OR | 0.12 (0.07-0.19)* |
|  | PDT vs placebo | 4 | 1300 | OR | 0.61 (0.46-0.83)* |
|  | IVTA vs placebo | 1 | 134 | OR | 0.91 (0.44-1.86) |
|  | brolucizumab vs aflibercept | 2 |  | OR | 0.96 (0.56-1.63) |
|  | ranibizumab vs aflibercept | 3 | 1208 | OR | 0.93 (0.55-1.53) |
|  | PDT vs aflibercept | 1 |  | OR | 3.81 (1.2-12.47)* |
|  | bevacizumab+PDT vs bevacizumab | 1 |  | OR | 0.96 (0-3002) |
|  | ranibizumab vs bevacizumab | 10 | 2734 | OR | 0.96 (0.69-1.35) |
|  | bevacizumab+PDT vs bevacizumab | 3 | 203 | OR | 1.1 (0.46-2.63) |
|  | DXM+ranibizumab vs ranibizumab | 1 | 242 | OR | 0.44 (0.05-2.42) |
|  | DXM+PDT+ranibizumab vs ranibizumab | 1 | 80 | OR | 1.06 (0.30-3.83) |
|  | PDT+ranibizumab vs ranibizumab | 5 | 232 | OR | 1.38 (0.66-3) |
|  | IVTA+PDT vs PDT | 2 | 87 | OR | 0.49 (0.15-1.46) |
|  | DXM+PDT+ranibizumab vs PDT+ranibizumab | 1 | 82 | OR | 0.67 (0.2-2.1) |
| **Mean BCVA**  (Between-study variance: MD, 5.64 [95% CrI, 2.88 -10.50]) | Conbercept vs placebo | 1 | 123 | MD | 1.20 (-4.06 to 6.43) |
|  | ranibizumab vs placebo | 2 | 602 | MD | 19.82 (15.00-24.57)* |
|  | PDT vs placebo | 1 | 364 | MD | 3.30 (0.20-6.40)* |
|  | brolucizumab vs aflibercept | 2 |  | MD | -0.45 (-4.13 to 3.16) |
|  | ranibizumab vs aflibercept | 5 | 1227 | MD | 0.43 (-2.16 to 3.11) |
|  | PDT vs aflibercept | 1 |  | MD | -6.58 (-10.16 to -3.01)* |
|  | DXM+PDT+ranibizumab vs PDT+ranibizumab | 1 | 82 | MD | 1.38 (-6.55 to 9.33) |
|  | bevacizumab+IVTA vs bevacizumab | 2 | 115 | MD | 3.34 (-0.78 to 7.49) |
|  | ranibizumab vs bevacizumab | 8 | 2578 | MD | -1.14 (-3.03 to 0.77) |
|  | bevacizumab+PDT vs bevacizumab | 2 | 108 | MD | -2.37 (-6.28 to 1.56) |
|  | DXM+PDT+ranibizumab vs ranibizumab | 1 | 80 | MD | -3.31 (-11.16 to 1.56) |
|  | PDT+ranibizumab vs ranibizumab | 8 | 541 | MD | -4.45 (-6.96 to -2.13)* |
|  | DXM+ranibizumab vs ranibizumab | 2 | 282 | MD | -1.23 (-5.73 to 3.00) |
|  | IVTA+PDT vs PDT | 1 | 100 | MD | 3.04 (2.21 to 3.85)* |
|  | bevacizumab+IVTA+PDT vs bevacizumab+PDT | 1 | 80 | MD | 1.38 (-6.36 to 12.52) |
| **Vision-related function**  (Between-study variance:  MD, 0.52 [95% CrI, 0.00-4.659]) | ranibizumab vs placebo | 1 | 716 | MD | 11.14 (8.81-13.51)* |
|  | ranibizumab vs aflibercept | 2 | 1192 | MD | 0.40 (-1.59-2.40) |
| **Mortality**  (Between-study variance:  OR, 0.01 [95% CrI, 0-0.18]) | Ranibizumab vs placebo | 2 | 599 | OR | 1.00 (0.30-3.21) |
|  | PDT vs placebo | 3 | 1312 | OR | 1.19 (0.58-2.60) |
|  | IVTA vs placebo | 1 | 151 | OR | 1.85 (0.41-9.72) |
|  | brolucizumab vs aflibercept |  |  | OR | 0.68 (0.23-1.91) |
|  | Ranibizumab vs aflibercept | 3 | 1192 | OR | 0.59 (0.15-1.98) |
|  | PDT vs aflibercept | 2 | 304 | OR | 0.74 (0.02-7.39) |
|  | Ranibizumab vs bevacizumab | 7 | 2668 | OR | 1.03 (0.60-1.75) |
|  | Bevacizumab+PDT vs bevacizumab | 1 | 23 | OR | 5.91 (0.15-3816) |
|  | DXM+PDT+ranibizumab vs ranibizumab | 1 | 80 | OR | 0.59 (0.11-2.70) |
|  | PDT+ranibizumab vs ranibizumab | 3 | 554 | OR | 0.56 (0.16-1.64) |
|  | IVTA+PDT vs PDT | 1 | 100 | OR | 0.10 (0.00-2.13) |
|  | DXM+PDT+ranibizumab vs PDT+ranibizumab | 1 | 82 | OR | 1.86 (0.27-17.11) |
| **Legal blindness**  (Between-study variance:  OR, 0.04 [95% CrI, 0.00-0.89]) | ranibizumab vs placebo | 2 | 602 | OR | 0.22 (0.12-0.44)* |
|  | PDT vs placebo | 1 | 364 | OR | 0.60 (0.39-0.95)* |
|  | ranibizumab vs bevacizumab | 3 | 1219 | OR | 0.00 (0.00-0.03)* |
| **Adverse events (AE)**  (Between-study variance:  OR, 0.02 [95% CrI, 0.00-0.19]) | ranibizumab vs placebo | 1 | 475 | OR | 1.68 (0.84-3.55) |
|  | PDT vs placebo | 3 | 1312 | OR | 1.54 (1.01-2.38)* |
|  | IVTA vs placebo | 1 | 151 | OR | 1.81 (0.41-10.14) |
|  | conbercept vs. placebo | 1 | 123 | OR | 0.98 (0.46-2.11) |
|  | ranibizumab vs aflibercept | 2 | 600 | OR | 1.19 (0.68-2.02) |
|  | PDT vs aflibercept | 2 | 304 | OR | 1.22 (0.76-1.94) |
|  | brolucizumab vs aflibercept | 2 |  |  | 1.07 (0.75-1.49) |
|  | ranibizumab vs bevacizumab | 2 | 540 | OR | 1.11 (0.70-1.95) |
|  | DXM+ranibizumab vs ranibizumab | 1 | 239 | OR | 2.48 (1.44-4.22)* |
| **Serious AE**  (Between-study variance: OR, 0.04 [95% CrI, 0.00-0.32]) | ranibizumab vs aflibercept | 1 | 600 | OR | 0.81 (0.55-1.17) |
|  | PDT vs aflibercept |  |  | OR | 1.16 (0.43-2.82) |
|  | Brolucizumab vs aflibercept | 2 |  | OR | 3.03 (1.22-8.33)* |
|  | ranibizumab vs bevacizumab | 4 | 1999 | OR | 0.86 (0.59-1.22) |
|  | PDT+ranibizumab vs ranibizumab | 2 | 104 | OR | 0.51 (0.18-1.32) |
|  | DXM+ranibizumab vs ranibizumab | 1 | 239 | OR | 0.59 (0.24-1.40) |
|  | DXM+PDT+ranibizumab vs ranibizumab | 1 | 80 | OR | 0.57 (0.22-1.44) |
|  | DXM+PDT+ranibizumab vs PDT+ranibizumab | 1 | 82 | OR | 0.92 (0.36-2.33) |
| **Withdrawals due to AE**  (Between-study variance: OR, 0.03 [95% CrI, 0.00-0.66]) | ranibizumab vs placebo | 2 | 599 | OR | 0.94 (0.35-2.24) |
|  | PDT vs placebo | 2 | 973 | OR | 4.88 (1.69-18.78)* |
|  | PDT vs aflibercept | 1 | 304 | OR | 1.71 (0.60-4.64) |
|  | ranibizumab vs bevacizumab | 2 | 1095 | OR | 1.20 (0.47-3.14) |
|  | PDT vs bevacizumab | 1 |  | OR | 1.08 (0.00 – 725.60) |
|  | bevacizumab+PDT vs bevacizumab | 1 | 112 | OR | 5.35 (0.19-1204.00) |
|  | PDT+ranibizumab vs ranibizumab | 1 | 255 | OR | 0.48 (0.06-2.80) |
|  | bevacizumab+PDT vs PDT | 1 | 112 | OR | 4.91 (0.15-2597.00) |
| **Arterial thromboembolic events**  (Between-study variance: OR, 0.03 [95% CrI, 0.00-0.46]) | ranibizumab vs placebo | 2 | 599 | OR | 1.27 (0.38-4.02) |
|  | Conbercept vs placebo | 1 | 123 | OR | 0.48 (0.01-22.51) |
|  | ranibizumab vs aflibercept | 2 | 1208 | OR | 1.78 (0.60-5.78) |
|  | PDT vs aflibercept | 1 |  | OR | 7.56 (0.52-248.60) |
|  | Brolucizumab vs aflibercept | 2 |  | OR | 0.65 (0.28-1.49) |
|  | ranibizumab vs bevacizumab | 5 | 2178 | OR | 1.60 (0.85-3.17) |
|  | PDT+ranibizumab vs ranibizumab | 1 | 255 | OR | 0.59 (0.15-2.04) |
|  | DXM+ranibizumab vs ranibizumab | 1 | 239 | OR | 0.40 (0.01-5.05) |
| **Venous thromboembolic events**  (Between-study variance:  OR, 0.13 [95% CrI, 0.01-2.45]) | ranibizumab vs aflibercept | 1 | 610 | OR | 5.18 (0.17-2552.00) |
|  | ranibizumab vs bevacizumab | 4 | 2123 | OR | 0.58 (0.16-1.91) |
| **Bacterial endophthalmitis**  (Between-study variance:  OR, 0.05 [95% CrI, 0.00-1.37]) | ranibizumab vs placebo | 1 | 475 | OR | 13.54 (0.83-8899.00) |
|  | ranibizumab vs aflibercept | 1 | 600 | OR | 0.21 (0.00-6.02) |
|  | brolucizumab vs aflibercept | 2 |  | OR | 5.70 (0.65-187.90) |
|  | ranibizumab vs bevacizumab | 2 | 1072 | OR | 0.77 (0.15-4.09) |
| **Retinal detachment**  (Between-study variance:  OR, 0.05 [95% CrI, 0.00-1.62]) | ranibizumab vs placebo | 1 | 477 | OR | 0.20 (0.00-5.61) |
|  | conbercept vs. placebo | 1 | 123 | OR | 0.09 (0.00-2.90) |
|  | ranibizumab vs aflibercept | 1 | 602 | OR | 14.11 (0.92-11500.00) |
|  | Brolucizumab vs afliberept | 2 |  | OR | 1.01 (0.09-11.90) |
|  | ranibizumab vs bevacizumab | 2 | 1197 | OR | 0.93 (0.09-9.78) |
|  | PDT+ranibizumab vs ranibizumab | 1 | 86 | OR | 4.91 (0.16-4762) |
|  | DXM+PDT+ranibizumab vs PDT+ranibizumab | 1 | 84 | OR | 0.20 (0.00-7.14) |

*indicates statistically significant results

**Abbreviations:** AE, adverse events; BCVA, Best-corrected visual acuity; CrI, credible interval; DXM, dexamethasone; IVTA, intravitreal triamcinolone acetonide; MD, mean difference; OR, odds ratio; PDT, photodynamic therapy; RCT, randomized controlled trials

# eTable 8: Sensitivity Network Meta-Analysis results

## **Outcome: VISION GAIN**

| **TREATMENT COMPARISON** | **Sample Size >100**  **Odds Ratio [(95% CrI) (95% PrI)** |
| --- | --- |
|  | **23 RCTs, 12 treatments, 8253 patients** |
| aflibercept vs placebo | 8.56 [(4.59-15.89) (4.08-17.77)] * |
| bevacizumab vs placebo | 8.22 [(4.3-15.55) (3.7-17.31)] * |
| bevacizumab vs aflibercept | 0.96 [(0.63-1.42) (0.52-1.68)] |
| ranibizumab vs placebo | 9.29 [(5.1-16.87) (4.36-18.56)] * |
| ranibizumab vs aflibercept | 1.09 [(0.77-1.48) (0.62-1.82)] |
| ranibizumab vs bevacizumab | 1.13 [(0.89-1.45) (0.7-1.86)] |
| PDT vs placebo | 2.26 [(1.24-4.17) (1.11-4.74)] * |
| PDT vs aflibercept | 0.26 [(0.14-0.49) (0.13-0.57)] * |
| PDT vs bevacizumab | 0.27 [(0.14-0.56) (0.13-0.63)] * |
| PDT vs ranibizumab | 0.24 [(0.13-0.47) (0.12-0.54)] * |
| PDT+ranibizumab vs placebo | 5.62 [(2.33-13.14) (2.08-13.96) *] |
| PDT+ranibizumab vs aflibercept | 0.65 [(0.32-1.28) (0.28-1.41)] |
| PDT+ranibizumab vs bevacizumab | 0.68 [(0.35-1.32) (0.31-1.47)] |
| PDT+ranibizumab vs ranibizumab | 0.6 [(0.33-1.1) (0.29-1.25)] |
| PDT+ranibizumab vs PDT | 2.47 [(1-5.9) (0.9-6.49)] |
| DXM+ranibizumab vs placebo | 6.33 [(1.84-20.46) (1.73-21.95)] * |
| DXM+ranibizumab vs aflibercept | 0.74 [(0.24-2.18) (0.22-2.37)] |
| DXM+ranibizumab vs bevacizumab | 0.77 [(0.25-2.25) (0.24-2.45)] |
| DXM+ranibizumab vs ranibizumab | 0.68 [(0.23-1.94) (0.21-2.09)] |
| DXM+ranibizumab vs PDT | 2.8 [(0.79-9.22) (0.73-9.74)] |
| DXM+ranibizumab vs PDT+ranibizumab | 1.12 [(0.33-3.81) (0.31-4.09)] |
| DXM+PDT+ranibizumab vs placebo | 4.73 [(1.26-16.13) (1.17-17.16)] * |
| DXM+PDT+ranibizumab vs aflibercept | 0.55 [(0.16-1.7) (0.15-1.82)] |
| DXM+PDT+ranibizumab vs bevacizumab | 0.58 [(0.17-1.77) (0.16-1.89)] |
| DXM+PDT+ranibizumab vs ranibizumab | 0.51 [(0.16-1.52) (0.15-1.62)] |
| DXM+PDT+ranibizumab vs PDT | 2.1 [(0.54-7.29) (0.5-7.83)] |
| DXM+PDT+ranibizumab vs PDT+ranibizumab | 0.85 [(0.25-2.56) (0.24-2.8)] |
| DXM+PDT+ranibizumab vs DXM+ranibizumab | 0.75 [(0.16-3.4) (0.15-3.59)] |
| bevacizumab+IVTA vs placebo | 20 [(6.55-58.18) (6.04-62.2)] * |
| bevacizumab+IVTA vs aflibercept | 2.32 [(0.88-6.04) (0.82-6.59)] |
| bevacizumab+IVTA vs bevacizumab | 2.41 [(1.02-5.89) (0.92-6.28)] * |
| bevacizumab+IVTA vs ranibizumab | 2.14 [(0.87-5.35) (0.79-5.94)] |
| bevacizumab+IVTA vs PDT | 8.83 [(2.84-26.23) (2.61-27.89)] * |
| bevacizumab+IVTA vs PDT+ranibizumab | 3.58 [(1.18-10.8) (1.1-11.45)] * |
| bevacizumab+IVTA vs DXM+ranibizumab | 3.16 [(0.78-13.13) (0.75-13.7)] |
| bevacizumab+IVTA vs DXM+PDT+ranibizumab | 4.22 [(1.03-18.98) (0.98-19.88)] * |
| brolucizumab vs placebo | 10.31 [(5.03-20.94) (4.43-23.32)] * |
| brolucizumab vs aflibercept | 1.2 [(0.83-1.76) (0.69-2.13)] |
| brolucizumab vs bevacizumab | 1.25 [(0.74-2.22) (0.64-2.59)] |
| brolucizumab vs ranibizumab | 1.11 [(0.69-1.86) (0.6-2.18)] |
| brolucizumab vs PDT | 4.57 [(2.21-9.16) (1.99-10.16)] * |
| brolucizumab vs PDT+ranibizumab | 1.84 [(0.86-4.14) (0.78-4.63)] |
| brolucizumab vs DXM+ranibizumab | 1.64 [(0.52-5.38) (0.48-5.66)] |
| brolucizumab vs DXM+PDT+ranibizumab | 2.18 [(0.67-7.98) (0.62-8.6)] |
| brolucizumab vs bevacizumab+IVTA | 0.52 [(0.19-1.48) (0.17-1.62)] |
| conbercept vs placebo | 1.57 [(0.57-4.74) (0.52-5.08)] |
| conbercept vs aflibercept | 0.18 [(0.06-0.65) (0.05-0.7)] * |
| conbercept vs bevacizumab | 0.19 [(0.06-0.69) (0.05-0.74)] * |
| conbercept vs ranibizumab | 0.17 [(0.05-0.59) (0.05-0.64)] * |
| conbercept vs PDT | 0.69 [(0.21-2.44) (0.19-2.59)] |
| conbercept vs PDT+ranibizumab | 0.28 [(0.08-1.12) (0.07-1.18)] |
| conbercept vs DXM+ranibizumab | 0.25 [(0.05-1.29) (0.05-1.38)] |
| conbercept vs DXM+PDT+ranibizumab | 0.34 [(0.07-1.8) (0.06-1.89)] |
| conbercept vs bevacizumab+IVTA | 0.08 [(0.02-0.37) (0.02-0.39)] * |
| conbercept vs brolucizumab | 0.15 [(0.04-0.57) (0.04-0.61)] * |
| **Between-study variance** | 0.03 (0.00-0.18) |

*indicates statistically significant results

**Abbreviations:** CrI, credible interval; DXM, dexamethasone; PDT, photodynamic therapy; PrI, prediction interval; RCT, randomized controlled trials

## **Outcome: VISION LOSS**

| **TREATMENT COMPARISON** | **Sample Size >100**  **Odds Ratio [(95% CrI) (95% PrI)** |
| --- | --- |
|  | **18 RCTs, 9 treatments, 6214 patients** |
| aflibercept vs placebo | 0.13 [(0.07-0.25) (0.07-0.27)]* |
| bevacizumab vs placebo | 0.13 [(0.07-0.23) (0.07-0.25)]* |
| bevacizumab vs aflibercept | 0.97 [(0.53-1.76) (0.49-1.89)] |
| ranibizumab vs placebo | 0.12 [(0.07-0.19) (0.07-0.21)]* |
| ranibizumab vs aflibercept | 0.9 [(0.55-1.43) (0.49-1.58)] |
| ranibizumab vs bevacizumab | 0.93 [(0.66-1.31) (0.58-1.49)] |
| PDT vs placebo | 0.61 [(0.46-0.81) (0.39-0.97)]* |
| PDT vs aflibercept | 4.52 [(2.39-8.75) (2.21-9.44)]* |
| PDT vs bevacizumab | 4.71 [(2.51-9) (2.32-9.77)]* |
| PDT vs ranibizumab | 5.05 [(3.02-8.68) (2.72-9.59)]* |
| PDT+ranibizumab vs placebo | 0.19 [(0.05-0.69) (0.05-0.7)]* |
| PDT+ranibizumab vs aflibercept | 1.4 [(0.38-5.11) (0.37-5.34)] |
| PDT+ranibizumab vs bevacizumab | 1.44 [(0.42-5.11) (0.4-5.42)] |
| PDT+ranibizumab vs ranibizumab | 1.55 [(0.47-5.24) (0.45-5.51)] |
| PDT+ranibizumab vs PDT | 0.31 [(0.08-1.14) (0.08-1.2)] |
| IVTA vs placebo | 0.9 [(0.41-1.97) (0.39-2.13)] |
| IVTA vs aflibercept | 6.72 [(2.54-18.41) (2.39-19.56)]* |
| IVTA vs bevacizumab | 6.98 [(2.66-18.78) (2.51-19.62)]* |
| IVTA vs ranibizumab | 7.5 [(3.08-18.84) (2.87-20.07)]* |
| IVTA vs PDT | 1.48 [(0.65-3.48) (0.61-3.68)] |
| IVTA vs PDT+ranibizumab | 4.86 [(1.09-22.03) (1.04-22.94)]* |
| DXM+ranibizumab vs placebo | 0.05 [(0.01-0.32) (0.01-0.33)]* |
| DXM+ranibizumab vs aflibercept | 0.38 [(0.04-2.41) (0.04-2.49)] |
| DXM+ranibizumab vs bevacizumab | 0.39 [(0.04-2.46) (0.04-2.55)] |
| DXM+ranibizumab vs ranibizumab | 0.43 [(0.05-2.55) (0.05-2.63)] |
| DXM+ranibizumab vs PDT | 0.08 [(0.01-0.54) (0.01-0.56)]* |
| DXM+ranibizumab vs PDT+ranibizumab | 0.27 [(0.02-2.44) (0.02-2.51)] |
| DXM+ranibizumab vs IVTA | 0.06 [(0.01-0.42) (0.01-0.43)]* |
| DXM+PDT+ranibizumab vs placebo | 0.13 [(0.03-0.5) (0.03-0.52)]* |
| DXM+PDT+ranibizumab vs aflibercept | 0.93 [(0.23-3.84) (0.22-3.87)] |
| DXM+PDT+ranibizumab vs bevacizumab | 0.96 [(0.25-3.8) (0.24-4)] |
| DXM+PDT+ranibizumab vs ranibizumab | 1.03 [(0.27-3.98) (0.26-4.07)] |
| DXM+PDT+ranibizumab vs PDT | 0.2 [(0.05-0.86) (0.05-0.88)]* |
| DXM+PDT+ranibizumab vs PDT+ranibizumab | 0.68 [(0.19-2.23) (0.18-2.3)] |
| DXM+PDT+ranibizumab vs IVTA | 0.14 [(0.03-0.67) (0.03-0.69)]* |
| DXM+PDT+ranibizumab vs DXM+ranibizumab | 2.46 [(0.27-29.65) (0.26-30.68)] |
| bevacizumab+IVTA vs placebo | 0.11 [(0-28.12) (0-28.78)] |
| bevacizumab+IVTA vs aflibercept | 0.81 [(0-204.7) (0-202.9)] |
| bevacizumab+IVTA vs bevacizumab | 0.83 [(0-209.5) (0-206.6)] |
| bevacizumab+IVTA vs ranibizumab | 0.9 [(0-233.3) (0-233.4)] |
| bevacizumab+IVTA vs PDT | 0.18 [(0-46.29) (0-45.17)] |
| bevacizumab+IVTA vs PDT+ranibizumab | 0.59 [(0-176.6) (0-178.4)] |
| bevacizumab+IVTA vs IVTA | 0.12 [(0-33.07) (0-32.16)] |
| bevacizumab+IVTA vs DXM+ranibizumab | 2.33 [(0-847.4) (0-841.5)] |
| bevacizumab+IVTA vs DXM+PDT+ranibizumab | 0.87 [(0-274.1) (0-277.4)] |
| brolucizumab vs placebo | 0.13 [(0.06-0.29) (0.05-0.31)]* |
| brolucizumab vs aflibercept | 0.97 [(0.57-1.62) (0.52-1.76)] |
| brolucizumab vs bevacizumab | 1 [(0.45-2.22) (0.43-2.37)] |
| brolucizumab vs ranibizumab | 1.08 [(0.53-2.22) (0.5-2.37)] |
| brolucizumab vs PDT | 0.21 [(0.09-0.49) (0.09-0.52)]* |
| brolucizumab vs PDT+ranibizumab | 0.69 [(0.17-2.8) (0.16-2.85)] |
| brolucizumab vs IVTA | 0.14 [(0.05-0.44) (0.04-0.46)]* |
| brolucizumab vs DXM+ranibizumab | 2.56 [(0.37-24.91) (0.36-25.23)] |
| brolucizumab vs DXM+PDT+ranibizumab | 1.03 [(0.23-4.65) (0.22-4.78)] |
| brolucizumab vs bevacizumab+IVTA | 1.2 [(0-504.6) (0-485.8)] |
| conbercept vs placebo | 0.03 [(0-0.62) (0-0.64)]* |
| conbercept vs aflibercept | 0.2 [(0-4.97) (0-5.05)] |
| conbercept vs bevacizumab | 0.21 [(0-5.26) (0-5.31)] |
| conbercept vs ranibizumab | 0.23 [(0-5.6) (0-5.75)] |
| conbercept vs PDT | 0.05 [(0-1.03) (0-1.04)] |
| conbercept vs PDT+ranibizumab | 0.14 [(0-4.47) (0-4.58)] |
| conbercept vs IVTA | 0.03 [(0-0.77) (0-0.77)]* |
| conbercept vs DXM+ranibizumab | 0.51 [(0-26.11) (0-26.54)] |
| conbercept vs DXM+PDT+ranibizumab | 0.21 [(0-6.78) (0-6.98)] |
| conbercept vs bevacizumab+IVTA | 0.2 [(0-192.9) (0-201)] |
| conbercept vs brolucizumab | 0.21 [(0-5.42) (0-5.52)] |
| **Between-study variance** | 0.02 (0-0.12) |

*indicates statistically significant results

**Abbreviations:** CrI, credible interval, DXM, dexamethasone; IVTA, intravitreal triamcinolone acetonide; NA, not applicable; PDT, photodynamic therapy; PrI, prediction interval; RCT, randomized controlled trial

# eTable 9: Surface Under the Cumulative Ranking Curve (SUCRA) Values for the Overall NMA and Subgroup Analyses for Vision Gain and Vision Loss

| **Treatment** | **Overall NMA SUCRA** | **Sample Size >100** |
| --- | --- | --- |
| **Vision Gain** | | |
| bevacizumab+IVTA | 1 (0.58-1) | 1 (0.6-1) |
| IVTA+PDT | 0.92 (0.17-1) | NA |
| brolucizumab | 0.83 (0.42-1) | 0.9 (0.5-1) |
| ranibizumab | 0.67 (0.5-0.92) | 0.8 (0.5-0.9) |
| DXM+ranibizumab | 0.67 (0.25-1) | 0.5 (0.2-1) |
| aflibercept | 0.58 (0.33-0.83) | 0.7 (0.4-0.9) |
| bevacizumab | 0.58 (0.33-0.83) | 0.6 (0.4-0.9) |
| bevacizumab+PDT | 0.5 (0.25-0.92) | NA |
| PDT+ranibizumab | 0.33 (0.25-0.75) | 0.4 (0.2-0.8) |
| DXM+PDT+ranibizumab | 0.33 (0.08-0.92) | 0.3 (0.1-0.9) |
| PDT | 0.17 (0.08-0.25) | 0.2 (0.1-0.3) |
| conbercept | 0.08 (0-0.33) | 0.1 (0-0.4) |
| placebo | 0 (0-0.08) | 0 (0-0.1) |
| **Vision Loss** | | |
| DXM+ranibizumab | 0.92 (0.31-1) | 0.91 (0.27-1) |
| conbercept | 0.92 (0.23-1) | 0.91 (0.18-1) |
| ranibizumab | 0.69 (0.46-0.85) | 0.64 (0.36-0.91) |
| DXM+PDT+ranibizumab | 0.69 (0.23-1) | 0.64 (0.27-1) |
| bevacizumab | 0.62 (0.38-0.85) | 0.55 (0.27-0.91) |
| brolucizumab | 0.62 (0.31-0.92) | 0.55 (0.27-0.91) |
| aflibercept | 0.54 (0.31-0.85) | 0.55 (0.27-0.82) |
| bevacizumab+PDT | 0.54 (0.23-0.92) | NA |
| bevacizumab+IVTA | 0.54 (0-1) | 0.73 (0-1) |
| PDT+ranibizumab | 0.46 (0.23-0.85) | 0.36 (0.18-0.91) |
| IVTA+PDT | 0.31 (0.08-0.85) | NA |
| PDT | 0.15 (0.08-0.31) | 0.18 (0.09-0.27) |
| placebo | 0.08 (0-0.15) | 0.09 (0-0.18) |
| IVTA | 0.08 (0-0.23) | 0.09 (0-0.27) |

**Abbreviations:** DXM, dexamethasone; IVTA, intravitreal triamcinolone acetonide; NA, not applicable; NMA, network meta-analysis; PDT, photodynamic therapy; ROB, risk of bias

# eTable 10: Surface Under the Cumulative Ranking Curve (SUCRA) Results for all Other Outcomes

| **Intervention** | | | **SUCRA (95% CrI)** |
| --- | --- | --- | --- |
| **Best-corrected visual acuity (BCVA)** | | | |
| bevacizumab+IVTA | | 1.00 (0.77-1.00) | |
| bevacizumab+IVTA+PDT | | 0.92 (0.23-1.00) | |
| bevacizumab | | 0.84 (0.62-0.92) | |
| ranibizumab | | 0.69 (0.54-0.92) | |
| aflibercept | | 0.62 (0.38-0.85) | |
| bevacizumab+PDT | | 0.62 (0.31-0.92) | |
| DXM+ranibizumab | | 0.62 (0.31-0.92) | |
| brolucizumab | | 0.54 (0.31-0.92) | |
| PDT+ranibizumab | | 0.39 (0.23-0.54) | |
| DXM+PDT+ranibizumab | | 0.39 (0.15-1.00) | |
| IVTA+PDT | | 0.23 (0.15-0.62) | |
| PDT | | 0.15 (0.08-0.23) | |
| conbercept | | 0.08 (0.00-0.23) | |
| placebo | | 0.00 (0.00-0.08) | |
| **Mortality** | | | |
| IVTA+PDT | 1.00 (0.10-1.00) | | |
| PDT+ranibizumab | 0.80 (0.20-1.00) | | |
| DXM+PDT+ranibizumab | 0.80 (0.10-1.00) | | |
| placebo | 0.50 (0.10-0.90) | | |
| bevacizumab | 0.50 (0.10-0.90) | | |
| ranibizumab | 0.50 (0.20-0.80) | | |
| PDT | 0.40 (0.00-0.90) | | |
| brolucizumab | 0.40 (0.00-1.00) | | |
| aflibercept | 0.20 (0.00-0.80) | | |
| IVTA | 0.20 (0.00-0.90) | | |
| bevacizumab+PDT | 0.00 (0.00-1.00) | | |
| **Arterial thromboembolic events (ATEs)** | | | |
| DXM+ranibizumab | 0.75 (0.00-1.00) | | |
| brolucizumab | 0.75 (0.25-1.00) | | |
| conbercept | 0.75 (0.00-1.00) | | |
| aflibercept | 0.63 (0.13-0.88) | | |
| PDT+ranibizumab | 0.63 (0.00-1.00) | | |
| bevacizumab | 0.50 (0.13-1.00) | | |
| placebo | 0.38 (0.00-0.88) | | |
| ranibizumab | 0.25 (0.00-0.63) | | |
| PDT | 0.00 (0.00-0.88) | | |
| **Adverse events (AEs)** | | | |
| placebo | 0.88 (0.50-1.00) | | |
| conbercept | 0.88 (0.13-1.00) | | |
| aflibercept | 0.63 (0.25-1.00) | | |
| bevacizumab | 0.50 (0.13-1.00) | | |
| brolucizumab | 0.50 (0.13-1.00) | | |
| ranibizumab | 0.38 (0.13-0.75) | | |
| PDT | 0.38 (0.13-0.75) | | |
| IVTA | 0.25 (0.00-1.00) | | |
| DXM+ranibizumab | 0.00 (0.00-0.13) | | |

**Abbreviations:** CrI, credible interval; DXM, dexamethasone; IVTA, intravitreal triamcinolone acetonide; PDT, photodynamic therapy

# eTable 11: Dose effects network meta-analysis (NMA) results

| **Treatment Comparison** | **NMA Estimate**  **(95% CrI) (95% PrI)** |
| --- | --- |
| **Proportion of patients experiencing VISION GAIN (≥15 ETDRS letters)**  21 RCTs, 11,629 patients, 4 treatments, 9 doses  Between-study variance: 0.01 (0.00-0.22)  Between-dose variance: 0.05 (0.01-0.16) | |
| ranibizumab (0.3mg) vs placebo | 8.29 [(5.08-14.2) (4.13-17)] * |
| ranibizumab (0.5mg) vs placebo | 9.16 [(5.61-15.31) (4.52-18.47)] * |
| ranibizumab (0.5mg) vs ranibizumab (0.3mg) | 1.08 [(0.9-1.42) (0.64-1.88)] |
| ranibizumab (2mg) vs placebo | 8.5 [(4.77-15.22) (4.01-18.15)] * |
| ranibizumab (2mg) vs ranibizumab (0.3mg) | 1.01 [(0.71-1.44) (0.56-1.83)] |
| ranibizumab (2mg) vs ranibizumab (0.5mg) | 0.95 [(0.64-1.22) (0.52-1.65)] |
| bevacizumab (1.25mg) vs placebo | 7.89 [(4.54-14.09) (3.67-16.47)] * |
| bevacizumab (1.25mg) vs ranibizumab (0.3mg) | 0.95 [(0.67-1.35) (0.51-1.71)] |
| bevacizumab (1.25mg) vs ranibizumab (0.5mg) | 0.86 [(0.66-1.12) (0.48-1.51)] |
| bevacizumab (1.25mg) vs ranibizumab (2mg) | 0.93 [(0.64-1.44) (0.49-1.76)] |
| bevacizumab (2.5mg) vs placebo | 7.61 [(3.82-15.08) (3.24-17.24)] * |
| bevacizumab (2.5mg) vs ranibizumab (0.3mg) | 0.91 [(0.54-1.58) (0.45-1.87)] |
| bevacizumab (2.5mg) vs ranibizumab (0.5mg) | 0.84 [(0.49-1.32) (0.41-1.62)] |
| bevacizumab (2.5mg) vs ranibizumab (2mg) | 0.89 [(0.51-1.6) (0.43-1.87)] |
| bevacizumab (2.5mg) vs bevacizumab (1.25mg) | 0.99 [(0.6-1.4) (0.5-1.78)] |
| aflibercept (0.5mg) vs placebo | 8.59 [(4.87-15.53) (4-18.42)] * |
| aflibercept (0.5mg) vs ranibizumab (0.3mg) | 1.03 [(0.69-1.55) (0.55-1.95)] |
| aflibercept (0.5mg) vs ranibizumab (0.5mg) | 0.94 [(0.66-1.34) (0.52-1.72)] |
| aflibercept (0.5mg) vs ranibizumab (2mg) | 1.02 [(0.64-1.62) (0.51-2.01)] |
| aflibercept (0.5mg) vs bevacizumab (1.25mg) | 1.09 [(0.7-1.71) (0.57-2.13)] |
| aflibercept (0.5mg) vs bevacizumab (2.5mg) | 1.13 [(0.63-2.06) (0.53-2.46)] |
| aflibercept (2mg) vs placebo | 9.37 [(5.36-16.57) (4.49-19.66)] * |
| aflibercept (2mg) vs ranibizumab (0.3mg) | 1.12 [(0.75-1.7) (0.59-2.14)] |
| aflibercept (2mg) vs ranibizumab (0.5mg) | 1.02 [(0.74-1.42) (0.57-1.85)] |
| aflibercept (2mg) vs ranibizumab (2mg) | 1.1 [(0.72-1.82) (0.57-2.18)] |
| aflibercept (2mg) vs bevacizumab (1.25mg) | 1.18 [(0.78-1.82) (0.63-2.29)] |
| aflibercept (2mg) vs bevacizumab (2.5mg) | 1.21 [(0.71-2.3) (0.59-2.72)] |
| aflibercept (2mg) vs aflibercept (0.5mg) | 1.06 [(0.87-1.49) (0.63-1.93)] |
| aflibercept (4mg) vs placebo | 8.22 [(3.64-15.82) (3.18-18.43)] * |
| aflibercept (4mg) vs ranibizumab (0.3mg) | 0.99 [(0.49-1.62) (0.42-1.99)] |
| aflibercept (4mg) vs ranibizumab (0.5mg) | 0.91 [(0.43-1.4) (0.38-1.78)] |
| aflibercept (4mg) vs ranibizumab (2mg) | 0.97 [(0.46-1.65) (0.4-1.99)] |
| aflibercept (4mg) vs bevacizumab (1.25mg) | 1.05 [(0.49-1.77) (0.43-2.18)] |
| aflibercept (4mg) vs bevacizumab (2.5mg) | 1.09 [(0.46-2.06) (0.41-2.41)] |
| aflibercept (4mg) vs aflibercept (0.5mg) | 0.98 [(0.49-1.35) (0.44-1.71)] |
| aflibercept (4mg) vs aflibercept (2mg) | 0.93 [(0.42-1.21) (0.38-1.59)] |
| PDT (reduced) vs placebo | 2.48 [(1.22-5.52) (1.04-6.31)] * |
| PDT (reduced) vs ranibizumab (0.3mg) | 0.29 [(0.14-0.71) (0.12-0.79)] * |
| PDT (reduced) vs ranibizumab (0.5mg) | 0.27 [(0.13-0.61) (0.11-0.71)] * |
| PDT (reduced) vs ranibizumab (2mg) | 0.29 [(0.13-0.73) (0.12-0.83)] * |
| PDT (reduced) vs bevacizumab (1.25mg) | 0.31 [(0.14-0.74) (0.13-0.86)] * |
| PDT (reduced) vs bevacizumab (2.5mg) | 0.32 [(0.14-0.88) (0.12-1.01)] * |
| PDT (reduced) vs aflibercept (0.5mg) | 0.29 [(0.14-0.68) (0.12-0.78)] * |
| PDT (reduced) vs aflibercept (2mg) | 0.27 [(0.13-0.59) (0.11-0.68)] * |
| PDT (reduced) vs aflibercept (4mg) | 0.3 [(0.14-0.92) (0.12-1)] * |
| PDT (standard) vs placebo | 2.42 [(1.34-4.44) (1.12-5.3)] * |
| PDT (standard) vs ranibizumab (0.3mg) | 0.29 [(0.15-0.56) (0.13-0.66)] * |
| PDT (standard) vs ranibizumab (0.5mg) | 0.26 [(0.14-0.5) (0.12-0.59)] * |
| PDT (standard) vs ranibizumab (2mg) | 0.28 [(0.14-0.58) (0.12-0.68)] * |
| PDT (standard) vs bevacizumab (1.25mg) | 0.31 [(0.15-0.61) (0.13-0.74)] * |
| PDT (standard) vs bevacizumab (2.5mg) | 0.32 [(0.15-0.71) (0.13-0.82)] * |
| PDT (standard) vs aflibercept (0.5mg) | 0.28 [(0.15-0.54) (0.13-0.65)] * |
| PDT (standard) vs aflibercept (2mg) | 0.26 [(0.14-0.48) (0.12-0.58)] * |
| PDT (standard) vs aflibercept (4mg) | 0.29 [(0.14-0.72) (0.13-0.81)] * |
| PDT (standard) vs PDT (reduced) | 0.99 [(0.55-1.53) (0.47-1.87)] |
| conbercept (0.5mg) vs placebo | 1.59 [(0.56-5.13) (0.5-5.53)] |
| conbercept (0.5mg) vs ranibizumab (0.3mg) | 0.19 [(0.06-0.67) (0.05-0.74)] * |
| conbercept (0.5mg) vs ranibizumab (0.5mg) | 0.17 [(0.05-0.61) (0.05-0.67)] * |
| conbercept (0.5mg) vs ranibizumab (2mg) | 0.19 [(0.06-0.67) (0.05-0.74)] * |
| conbercept (0.5mg) vs bevacizumab (1.25mg) | 0.2 [(0.06-0.73) (0.06-0.8)] * |
| conbercept (0.5mg) vs bevacizumab (2.5mg) | 0.21 [(0.06-0.79) (0.05-0.86) *] |
| conbercept (0.5mg) vs aflibercept (0.5mg) | 0.19 [(0.06-0.67) (0.05-0.75)] * |
| conbercept (0.5mg) vs aflibercept (2mg) | 0.17 [(0.05-0.61) (0.05-0.66)] * |
| conbercept (0.5mg) vs aflibercept (4mg) | 0.2 [(0.06-0.79) (0.05-0.86)] * |
| conbercept (0.5mg) vs PDT (reduced) | 0.64 [(0.17-2.5) (0.16-2.74)] |
| conbercept (0.5mg) vs PDT (standard) | 0.66 [(0.2-2.42) (0.18-2.61)] |
| brolucizumab (3mg) vs placebo | 9.64 [(4.75-19.83) (4.08-23.16)] * |
| brolucizumab (3mg) vs ranibizumab (0.3mg) | 1.16 [(0.64-2.09) (0.54-2.49)] |
| brolucizumab (3mg) vs ranibizumab (0.5mg) | 1.05 [(0.6-1.85) (0.51-2.27)] |
| brolucizumab (3mg) vs ranibizumab (2mg) | 1.13 [(0.61-2.17) (0.51-2.55)] |
| brolucizumab (3mg) vs bevacizumab (1.25mg) | 1.22 [(0.67-2.31) (0.56-2.71)] |
| brolucizumab (3mg) vs bevacizumab (2.5mg) | 1.26 [(0.62-2.64) (0.55-3.06)] |
| brolucizumab (3mg) vs aflibercept (0.5mg) | 1.12 [(0.68-1.86) (0.56-2.27)] |
| brolucizumab (3mg) vs aflibercept (2mg) | 1.03 [(0.65-1.63) (0.52-2.02)] |
| brolucizumab (3mg) vs aflibercept (4mg) | 1.17 [(0.68-2.52) (0.56-2.85)] |
| brolucizumab (3mg) vs PDT (reduced) | 3.9 [(1.49-8.93) (1.32-10.17)] * |
| brolucizumab (3mg) vs PDT (standard) | 3.98 [(1.86-8.37) (1.64-9.69)] * |
| brolucizumab (3mg) vs conbercept (0.5mg) | 6.02 [(1.57-21) (1.47-22.85)] * |
| brolucizumab (6mg) vs placebo | 10.68 [(5.32-21.23) (4.49-24.6)] * |
| brolucizumab (6mg) vs ranibizumab (0.3mg) | 1.28 [(0.72-2.28) (0.59-2.7)] |
| brolucizumab (6mg) vs ranibizumab (0.5mg) | 1.16 [(0.7-1.96) (0.56-2.42)] |
| brolucizumab (6mg) vs ranibizumab (2mg) | 1.25 [(0.7-2.38) (0.58-2.82)] |
| brolucizumab (6mg) vs bevacizumab (1.25mg) | 1.35 [(0.76-2.44) (0.64-2.95)] |
| brolucizumab (6mg) vs bevacizumab (2.5mg) | 1.38 [(0.71-2.99) (0.6-3.42)] |
| brolucizumab (6mg) vs aflibercept (0.5mg) | 1.23 [(0.77-2.04) (0.63-2.48)] |
| brolucizumab (6mg) vs aflibercept (2mg) | 1.14 [(0.76-1.69) (0.6-2.12)] |
| brolucizumab (6mg) vs aflibercept (4mg) | 1.27 [(0.76-2.99) (0.61-3.37)] |
| brolucizumab (6mg) vs PDT (reduced) | 4.28 [(1.77-9.75) (1.54-11.13)] * |
| brolucizumab (6mg) vs PDT (standard) | 4.43 [(2.09-8.98) (1.79-10.4)] * |
| brolucizumab (6mg) vs conbercept (0.5mg) | 6.69 [(1.74-22.86) (1.63-25.14)] * |
| brolucizumab (6mg) vs brolucizumab (3mg) | 1.07 [(0.83-1.67) (0.62-2.01)] |
| **Proportion of patients experiencing VISION LOSS (≥15 ETDRS letters)**  20 RCTs, 10,496 patients, 5 treatments, 10 doses  Between-study variance: 0.04 (0.00-0.64)  Between-dose variance: 0.02 (0.00-0.14) | |
| ranibizumab (0.3mg) vs placebo | 0.12 [(0.07-0.19) (0.07-0.22)] * |
| ranibizumab (0.5mg) vs placebo | 0.12 [(0.08-0.18) (0.07-0.21)] * |
| ranibizumab (0.5mg) vs ranibizumab (0.3mg) | 1 [(0.68-1.49) (0.59-1.7)] |
| ranibizumab (2mg) vs placebo | 0.14 [(0.08-0.41) (0.07-0.42)] * |
| ranibizumab (2mg) vs ranibizumab (0.3mg) | 1.15 [(0.78-3.32) (0.67-3.57)] |
| ranibizumab (2mg) vs ranibizumab (0.5mg) | 1.16 [(0.81-3.09) (0.69-3.27)] |
| bevacizumab (1.25mg) vs placebo | 0.13 [(0.07-0.22) (0.07-0.25)] * |
| bevacizumab (1.25mg) vs ranibizumab (0.3mg) | 1.06 [(0.64-1.81) (0.56-2.02)] |
| bevacizumab (1.25mg) vs ranibizumab (0.5mg) | 1.07 [(0.76-1.51) (0.64-1.77)] |
| bevacizumab (1.25mg) vs ranibizumab (2mg) | 0.89 [(0.33-1.53) (0.32-1.72)] |
| bevacizumab (2.5mg) vs placebo | 0.15 [(0.07-0.41) (0.06-0.44)] * |
| bevacizumab (2.5mg) vs ranibizumab (0.3mg) | 1.21 [(0.63-3.56) (0.57-3.7)] |
| bevacizumab (2.5mg) vs ranibizumab (0.5mg) | 1.22 [(0.7-3.19) (0.62-3.38)] |
| bevacizumab (2.5mg) vs ranibizumab (2mg) | 1.02 [(0.38-2.38) (0.37-2.51)] |
| bevacizumab (2.5mg) vs bevacizumab (1.25mg) | 1.1 [(0.73-2.82) (0.64-2.98)] |
| aflibercept (0.5mg) vs placebo | 0.12 [(0.06-0.22) (0.06-0.24)] * |
| aflibercept (0.5mg) vs ranibizumab (0.3mg) | 0.98 [(0.53-1.78) (0.48-1.94)] |
| aflibercept (0.5mg) vs ranibizumab (0.5mg) | 0.98 [(0.6-1.59) (0.53-1.81)] |
| aflibercept (0.5mg) vs ranibizumab (2mg) | 0.81 [(0.27-1.53) (0.26-1.68)] |
| aflibercept (0.5mg) vs bevacizumab (1.25mg) | 0.92 [(0.5-1.67) (0.46-1.88)] |
| aflibercept (0.5mg) vs bevacizumab (2.5mg) | 0.79 [(0.27-1.7) (0.26-1.85)] |
| aflibercept (2mg) vs placebo | 0.12 [(0.07-0.22) (0.06-0.25)] * |
| aflibercept (2mg) vs ranibizumab (0.3mg) | 1.04 [(0.59-1.86) (0.54-2.04)] |
| aflibercept (2mg) vs ranibizumab (0.5mg) | 1.04 [(0.66-1.65) (0.59-1.86)] |
| aflibercept (2mg) vs ranibizumab (2mg) | 0.85 [(0.31-1.58) (0.3-1.78)] |
| aflibercept (2mg) vs bevacizumab (1.25mg) | 0.97 [(0.54-1.72) (0.49-1.92)] |
| aflibercept (2mg) vs bevacizumab (2.5mg) | 0.84 [(0.3-1.75) (0.29-1.92)] |
| aflibercept (2mg) vs aflibercept (0.5mg) | 1.04 [(0.74-1.64) (0.64-1.87)] |
| aflibercept (4mg) vs placebo | 0.11 [(0.03-0.23) (0.03-0.25)] * |
| aflibercept (4mg) vs ranibizumab (0.3mg) | 0.91 [(0.27-1.88) (0.26-2.04)] |
| aflibercept (4mg) vs ranibizumab (0.5mg) | 0.91 [(0.28-1.76) (0.27-1.95)] |
| aflibercept (4mg) vs ranibizumab (2mg) | 0.77 [(0.13-1.58) (0.13-1.74)] |
| aflibercept (4mg) vs bevacizumab (1.25mg) | 0.84 [(0.25-1.8) (0.24-1.97)] |
| aflibercept (4mg) vs bevacizumab (2.5mg) | 0.75 [(0.13-1.71) (0.13-1.87)] |
| aflibercept (4mg) vs aflibercept (0.5mg) | 0.96 [(0.31-1.6) (0.3-1.77)] |
| aflibercept (4mg) vs aflibercept (2mg) | 0.92 [(0.28-1.45) (0.27-1.64)] |
| IVTA (4mg) vs placebo | 0.92 [(0.4-1.97) (0.37-2.13)] |
| IVTA (4mg) vs ranibizumab (0.3mg) | 7.78 [(3-18.96) (2.86-20.66)] * |
| IVTA (4mg) vs ranibizumab (0.5mg) | 7.75 [(3.07-18.95) (2.87-20.27)] * |
| IVTA (4mg) vs ranibizumab (2mg) | 6.15 [(1.8-17.29) (1.7-18.49)] * |
| IVTA (4mg) vs bevacizumab (1.25mg) | 7.23 [(2.74-19.67) (2.57-20.7)] * |
| IVTA (4mg) vs bevacizumab (2.5mg) | 6.09 [(1.75-19.09) (1.66-19.94)] * |
| IVTA (4mg) vs aflibercept (0.5mg) | 7.98 [(2.86-21.54) (2.66-22.96)] * |
| IVTA (4mg) vs aflibercept (2mg) | 7.48 [(2.75-19.73) (2.55-20.65)] * |
| IVTA (4mg) vs aflibercept (4mg) | 8.85 [(2.84-34.75) (2.69-36.27)] * |
| PDT (reduced) vs placebo | 0.36 [(0.14-0.6) (0.13-0.68)] * |
| PDT (reduced) vs ranibizumab (0.3mg) | 3 [(1.04-5.96) (0.98-6.55)] * |
| PDT (reduced) vs ranibizumab (0.5mg) | 3 [(1.03-5.94) (0.98-6.5)] * |
| PDT (reduced) vs ranibizumab (2mg) | 2.53 [(0.47-5.71) (0.46-6.13)] |
| PDT (reduced) vs bevacizumab (1.25mg) | 2.77 [(0.9-6.07) (0.86-6.52)] |
| PDT (reduced) vs bevacizumab (2.5mg) | 2.43 [(0.48-6.03) (0.47-6.57)] |
| PDT (reduced) vs aflibercept (0.5mg) | 3.03 [(1-6.67) (0.95-7.17)] * |
| PDT (reduced) vs aflibercept (2mg) | 2.86 [(0.92-6.25) (0.87-6.69)] |
| PDT (reduced) vs aflibercept (4mg) | 3.39 [(1.04-9.5) (0.99-10.05)] * |
| PDT (reduced) vs IVTA (4mg) | 0.38 [(0.12-1.04) (0.11-1.11)] |
| PDT (standard) vs placebo | 0.46 [(0.34-0.62) (0.28-0.73)]* |
| PDT (standard) vs ranibizumab (0.3mg) | 3.83 [(2.27-6.65) (2.02-7.35)] * |
| PDT (standard) vs ranibizumab (0.5mg) | 3.84 [(2.33-6.44) (2.06-7.25)] * |
| PDT (standard) vs ranibizumab (2mg) | 3.14 [(1.13-6.02) (1.06-6.56)] * |
| PDT (standard) vs bevacizumab (1.25mg) | 3.6 [(1.95-6.71) (1.76-7.37)] * |
| PDT (standard) vs bevacizumab (2.5mg) | 3.08 [(1.1-6.74) (1.04-7.3)] * |
| PDT (standard) vs aflibercept (0.5mg) | 3.93 [(2.06-7.65) (1.84-8.36)] * |
| PDT (standard) vs aflibercept (2mg) | 3.72 [(1.99-6.84) (1.77-7.58)] * |
| PDT (standard) vs aflibercept (4mg) | 4.27 [(1.93-14.84) (1.78-15.34)] * |
| PDT (standard) vs IVTA (4mg) | 0.5 [(0.22-1.19) (0.2-1.3)] |
| PDT (standard) vs PDT (reduced) | 1.22 [(0.83-3.39) (0.71-3.58)] |
| conbercept (0.5mg) vs placebo | 0.03 [(0-0.65) (0-0.65)]* |
| conbercept (0.5mg) vs ranibizumab (0.3mg) | 0.28 [(0-5.59) (0-5.7)] |
| conbercept (0.5mg) vs ranibizumab (0.5mg) | 0.28 [(0-5.57) (0-5.71)] |
| conbercept (0.5mg) vs ranibizumab (2mg) | 0.21 [(0-4.56) (0-4.65)] |
| conbercept (0.5mg) vs bevacizumab (1.25mg) | 0.26 [(0-5.44) (0-5.54)] |
| conbercept (0.5mg) vs bevacizumab (2.5mg) | 0.21 [(0-4.68) (0-4.78)] |
| conbercept (0.5mg) vs aflibercept (0.5mg) | 0.28 [(0-5.9) (0-6.12)] |
| conbercept (0.5mg) vs aflibercept (2mg) | 0.27 [(0-5.54) (0-5.69)] |
| conbercept (0.5mg) vs aflibercept (4mg) | 0.33 [(0-8.2) (0-8.35)] |
| conbercept (0.5mg) vs IVTA (4mg) | 0.04 [(0-0.79) (0-0.82)]* |
| conbercept (0.5mg) vs PDT (reduced) | 0.1 [(0-2.22) (0-2.3)] |
| conbercept (0.5mg) vs PDT (standard) | 0.07 [(0-1.44) (0-1.47)] |
| brolucizumab (3mg) vs placebo | 0.12 [(0.05-0.27) (0.05-0.3)]* |
| brolucizumab (3mg) vs ranibizumab (0.3mg) | 1 [(0.46-2.25) (0.42-2.41)] |
| brolucizumab (3mg) vs ranibizumab (0.5mg) | 1 [(0.5-2.1) (0.45-2.29)] |
| brolucizumab (3mg) vs ranibizumab (2mg) | 0.81 [(0.26-1.9) (0.24-2.05)] |
| brolucizumab (3mg) vs bevacizumab (1.25mg) | 0.93 [(0.43-2.13) (0.4-2.27)] |
| brolucizumab (3mg) vs bevacizumab (2.5mg) | 0.8 [(0.25-2.05) (0.24-2.23)] |
| brolucizumab (3mg) vs aflibercept (0.5mg) | 1.03 [(0.52-2.06) (0.47-2.23)] |
| brolucizumab (3mg) vs aflibercept (2mg) | 0.97 [(0.55-1.7) (0.49-1.89)] |
| brolucizumab (3mg) vs aflibercept (4mg) | 1.11 [(0.52-3.81) (0.48-4.02)] |
| brolucizumab (3mg) vs IVTA (4mg) | 0.13 [(0.04-0.41) (0.04-0.44)]* |
| brolucizumab (3mg) vs PDT (reduced) | 0.34 [(0.13-1.19) (0.12-1.24)] |
| brolucizumab (3mg) vs PDT (standard) | 0.26 [(0.11-0.61) (0.11-0.66)]* |
| brolucizumab (3mg) vs conbercept (0.5mg) | 3.68 [(0.16-2316) (0.16-2286)] |
| brolucizumab (6mg) vs placebo | 0.12 [(0.06-0.26) (0.05-0.28)]* |
| brolucizumab (6mg) vs ranibizumab (0.3mg) | 1 [(0.48-2.14) (0.43-2.34)] |
| brolucizumab (6mg) vs ranibizumab (0.5mg) | 1 [(0.52-1.99) (0.47-2.17)] |
| brolucizumab (6mg) vs ranibizumab (2mg) | 0.81 [(0.26-1.84) (0.26-2)] |
| brolucizumab (6mg) vs bevacizumab (1.25mg) | 0.93 [(0.45-2.03) (0.41-2.17)] |
| brolucizumab (6mg) vs bevacizumab (2.5mg) | 0.79 [(0.26-1.97) (0.25-2.11)] |
| brolucizumab (6mg) vs aflibercept (0.5mg) | 1.02 [(0.55-1.93) (0.5-2.12)] |
| brolucizumab (6mg) vs aflibercept (2mg) | 0.97 [(0.58-1.58) (0.52-1.79)] |
| brolucizumab (6mg) vs aflibercept (4mg) | 1.11 [(0.54-3.66) (0.5-3.87)] |
| brolucizumab (6mg) vs IVTA (4mg) | 0.13 [(0.04-0.4) (0.04-0.42)]* |
| brolucizumab (6mg) vs PDT (reduced) | 0.34 [(0.13-1.14) (0.12-1.21)] |
| brolucizumab (6mg) vs PDT (standard) | 0.26 [(0.12-0.58) (0.11-0.63)]* |
| brolucizumab (6mg) vs conbercept (0.5mg) | 3.67 [(0.17-2322) (0.16-2274)] |
| brolucizumab (6mg) vs brolucizumab (3mg) | 1 [(0.63-1.6) (0.57-1.78)] |
| *indicates statistically significant results  **Abbreviations:** CrI, credible interval, IVTA, intravitreal triamcinolone acetonide; PDT, photodynamic therapy; PrI, prediction interval; RCT, randomized controlled trial | |

# eTable 12: Confidence in Network Meta-Analysis (CINeMA) assessment for the outcome of vision gain

| **Comparison** | **Number of studies** | **Within-study bias** | **Reporting bias** | **Indirectness** | **Imprecision** | **Heterogeneity** | **Incoherence** | **Confidence rating** | **Reason(s) for downgrading** |
| --- | --- | --- | --- | --- | --- | --- | --- | --- | --- |
| DXM+PDT+ranibizumab:PDT+ranibizumab | 1 | Some concerns | Low risk | No concerns | Major concerns | No concerns | No concerns | Moderate | ["Imprecision"] |
| DXM+PDT+ranibizumab:ranibizumab | 1 | Some concerns | Low risk | No concerns | Major concerns | No concerns | No concerns | Moderate | ["Imprecision"] |
| DXM+ranibizumab:ranibizumab | 2 | Major concerns | Low risk | No concerns | Major concerns | No concerns | No concerns | Low | ["Within-study bias","Imprecision"] |
| IVTA+PDT:PDT | 1 | Some concerns | Low risk | No concerns | Major concerns | No concerns | No concerns | Moderate | ["Imprecision"] |
| aflibercept:PDT | 1 | Major concerns | Low risk | No concerns | No concerns | No concerns | No concerns | Moderate | ["Within-study bias"] |
| PDT:placebo | 3 | Major concerns | Low risk | No concerns | No concerns | Some concerns | No concerns | Moderate | ["Within-study bias"] |
| PDT+ranibizumab:ranibizumab | 6 | Major concerns | Low risk | No concerns | Some concerns | Some concerns | No concerns | Moderate | ["Within-study bias"] |
| **aflibercept:brolucizumab** | **2** | **Major concerns** | **Low risk** | **No concerns** | **Some concerns** | **Some concerns** | **No concerns** | **Moderate** | **["Within-study bias"]** |
| **aflibercept:ranibizumab** | **3** | **Major concerns** | **Low risk** | **No concerns** | **Major concerns** | **No concerns** | **No concerns** | **Low** | **["Within-study bias","Imprecision"]** |
| bevacizumab:bevacizumab+IVTA | 1 | Some concerns | Low risk | No concerns | Some concerns | No concerns | No concerns | High | [] |
| bevacizumab:bevacizumab+PDT | 3 | Some concerns | Low risk | No concerns | Major concerns | No concerns | No concerns | Moderate | ["Imprecision"] |
| **bevacizumab:ranibizumab** | **9** | **No concerns** | **Low risk** | **No concerns** | **Some concerns** | **Some concerns** | **No concerns** | **High** | **[]** |
| conbercept:placebo | 1 | No concerns | Low risk | No concerns | Major concerns | No concerns | No concerns | Moderate | ["Imprecision"] |
| placebo:ranibizumab | 2 | Major concerns | Low risk | No concerns | No concerns | No concerns | No concerns | Moderate | ["Within-study bias"] |
| DXM+PDT+ranibizumab:DXM+ranibizumab | 0 | Some concerns | Low risk | No concerns | Major concerns | No concerns | No concerns | Moderate | ["Imprecision"] |
| DXM+PDT+ranibizumab:IVTA+PDT | 0 | Some concerns | Low risk | No concerns | Major concerns | No concerns | No concerns | Moderate | ["Imprecision"] |
| DXM+PDT+ranibizumab:PDT | 0 | Major concerns | Low risk | No concerns | Major concerns | No concerns | No concerns | Low | ["Within-study bias","Imprecision"] |
| aflibercept:DXM+PDT+ranibizumab | 0 | Major concerns | Low risk | No concerns | Major concerns | No concerns | No concerns | Low | ["Within-study bias","Imprecision"] |
| bevacizumab:DXM+PDT+ranibizumab | 0 | Some concerns | Low risk | No concerns | Major concerns | No concerns | No concerns | Moderate | ["Imprecision"] |
| bevacizumab+IVTA:DXM+PDT+ranibizumab | 0 | Some concerns | Low risk | No concerns | Some concerns | Some concerns | No concerns | Moderate | [] |
| bevacizumab+PDT:DXM+PDT+ranibizumab | 0 | Some concerns | Low risk | No concerns | Major concerns | No concerns | No concerns | Moderate | ["Imprecision"] |
| brolucizumab:DXM+PDT+ranibizumab | 0 | Major concerns | Low risk | No concerns | Major concerns | No concerns | No concerns | Low | ["Within-study bias","Imprecision"] |
| conbercept:DXM+PDT+ranibizumab | 0 | Some concerns | Low risk | No concerns | Major concerns | No concerns | No concerns | Moderate | ["Imprecision"] |
| DXM+PDT+ranibizumab:placebo | 0 | Some concerns | Low risk | No concerns | No concerns | No concerns | No concerns | High | [] |
| DXM+ranibizumab:IVTA+PDT | 0 | Major concerns | Low risk | No concerns | Major concerns | No concerns | No concerns | Low | ["Within-study bias","Imprecision"] |
| DXM+ranibizumab:PDT | 0 | Major concerns | Low risk | No concerns | No concerns | No concerns | No concerns | Moderate | ["Within-study bias"] |
| DXM+ranibizumab:PDT+ranibizumab | 0 | Major concerns | Low risk | No concerns | Major concerns | No concerns | No concerns | Low | ["Within-study bias","Imprecision"] |
| aflibercept:DXM+ranibizumab | 0 | Major concerns | Low risk | No concerns | Major concerns | No concerns | No concerns | Low | ["Within-study bias","Imprecision"] |
| bevacizumab:DXM+ranibizumab | 0 | Some concerns | Low risk | No concerns | Major concerns | No concerns | No concerns | Moderate | ["Imprecision"] |
| bevacizumab+IVTA:DXM+ranibizumab | 0 | Some concerns | Low risk | No concerns | Major concerns | No concerns | No concerns | Moderate | ["Imprecision"] |
| bevacizumab+PDT:DXM+ranibizumab | 0 | Some concerns | Low risk | No concerns | Major concerns | No concerns | No concerns | Moderate | ["Imprecision"] |
| brolucizumab:DXM+ranibizumab | 0 | Major concerns | Low risk | No concerns | Major concerns | No concerns | No concerns | Low | ["Within-study bias","Imprecision"] |
| conbercept:DXM+ranibizumab | 0 | Some concerns | Low risk | No concerns | No concerns | No concerns | No concerns | High | [] |
| DXM+ranibizumab:placebo | 0 | Major concerns | Low risk | No concerns | No concerns | No concerns | No concerns | Moderate | ["Within-study bias"] |
| IVTA+PDT:PDT+ranibizumab | 0 | Major concerns | Low risk | No concerns | Major concerns | No concerns | No concerns | Low | ["Within-study bias","Imprecision"] |
| aflibercept:IVTA+PDT | 0 | Major concerns | Low risk | No concerns | Major concerns | No concerns | No concerns | Low | ["Within-study bias","Imprecision"] |
| bevacizumab:IVTA+PDT | 0 | Some concerns | Low risk | No concerns | Major concerns | No concerns | No concerns | Moderate | ["Imprecision"] |
| bevacizumab+IVTA:IVTA+PDT | 0 | Some concerns | Low risk | No concerns | Major concerns | No concerns | No concerns | Moderate | ["Imprecision"] |
| bevacizumab+PDT:IVTA+PDT | 0 | Some concerns | Low risk | No concerns | Major concerns | No concerns | No concerns | Moderate | ["Imprecision"] |
| brolucizumab:IVTA+PDT | 0 | Major concerns | Low risk | No concerns | Major concerns | No concerns | No concerns | Low | ["Within-study bias","Imprecision"] |
| conbercept:IVTA+PDT | 0 | Some concerns | Low risk | No concerns | Major concerns | No concerns | No concerns | Moderate | ["Imprecision"] |
| IVTA+PDT:placebo | 0 | Major concerns | Low risk | No concerns | Some concerns | Some concerns | No concerns | Moderate | ["Within-study bias"] |
| IVTA+PDT:ranibizumab | 0 | Major concerns | Low risk | No concerns | Major concerns | No concerns | No concerns | Low | ["Within-study bias","Imprecision"] |
| PDT:PDT+ranibizumab | 0 | Major concerns | Low risk | No concerns | No concerns | Some concerns | No concerns | Moderate | ["Within-study bias"] |
| bevacizumab:PDT | 0 | Some concerns | Low risk | No concerns | No concerns | No concerns | No concerns | High | [] |
| bevacizumab+IVTA:PDT | 0 | Some concerns | Low risk | No concerns | No concerns | No concerns | No concerns | High | [] |
| bevacizumab+PDT:PDT | 0 | Some concerns | Low risk | No concerns | No concerns | No concerns | No concerns | High | [] |
| brolucizumab:PDT | 0 | Major concerns | Low risk | No concerns | No concerns | No concerns | No concerns | Moderate | ["Within-study bias"] |
| conbercept:PDT | 0 | Some concerns | Low risk | No concerns | Major concerns | No concerns | No concerns | Moderate | ["Imprecision"] |
| PDT:ranibizumab | 0 | Major concerns | Low risk | No concerns | No concerns | No concerns | No concerns | Moderate | ["Within-study bias"] |
| aflibercept:PDT+ranibizumab | 0 | Major concerns | Low risk | No concerns | Major concerns | No concerns | No concerns | Low | ["Within-study bias","Imprecision"] |
| bevacizumab:PDT+ranibizumab | 0 | Some concerns | Low risk | No concerns | Major concerns | No concerns | No concerns | Moderate | ["Imprecision"] |
| bevacizumab+IVTA:PDT+ranibizumab | 0 | Some concerns | Low risk | No concerns | No concerns | No concerns | No concerns | High | [] |
| bevacizumab+PDT:PDT+ranibizumab | 0 | Some concerns | Low risk | No concerns | Major concerns | No concerns | No concerns | Moderate | ["Imprecision"] |
| brolucizumab:PDT+ranibizumab | 0 | Major concerns | Low risk | No concerns | Some concerns | Some concerns | No concerns | Moderate | ["Within-study bias"] |
| conbercept:PDT+ranibizumab | 0 | Some concerns | Low risk | No concerns | Some concerns | Some concerns | No concerns | Moderate | [] |
| PDT+ranibizumab:placebo | 0 | Major concerns | Low risk | No concerns | No concerns | No concerns | No concerns | Moderate | ["Within-study bias"] |
| **aflibercept:bevacizumab** | **0** | **Some concerns** | **Low risk** | **No concerns** | **Major concerns** | **No concerns** | **No concerns** | **Moderate** | **["Imprecision"]** |
| aflibercept:bevacizumab+IVTA | 0 | Some concerns | Low risk | No concerns | Some concerns | Some concerns | No concerns | Moderate | [] |
| aflibercept:bevacizumab+PDT | 0 | Some concerns | Low risk | No concerns | Major concerns | No concerns | No concerns | Moderate | ["Imprecision"] |
| **aflibercept:conbercept** | **0** | **Some concerns** | **Low risk** | **No concerns** | **No concerns** | **No concerns** | **No concerns** | **High** | **[]** |
| aflibercept:placebo | 0 | Major concerns | Low risk | No concerns | No concerns | No concerns | No concerns | Moderate | ["Within-study bias"] |
| **bevacizumab:brolucizumab** | **0** | **Some concerns** | **Low risk** | **No concerns** | **Major concerns** | **No concerns** | **No concerns** | **Moderate** | **["Imprecision"]** |
| **bevacizumab:conbercept** | **0** | **Some concerns** | **Low risk** | **No concerns** | **No concerns** | **No concerns** | **No concerns** | **High** | **[]** |
| bevacizumab:placebo | 0 | Some concerns | Low risk | No concerns | No concerns | No concerns | No concerns | High | [] |
| bevacizumab+IVTA:bevacizumab+PDT | 0 | Some concerns | Low risk | No concerns | Some concerns | Some concerns | No concerns | Moderate | [] |
| bevacizumab+IVTA:brolucizumab | 0 | Some concerns | Low risk | No concerns | Major concerns | No concerns | No concerns | Moderate | ["Imprecision"] |
| bevacizumab+IVTA:conbercept | 0 | Some concerns | Low risk | No concerns | No concerns | No concerns | No concerns | High | [] |
| bevacizumab+IVTA:placebo | 0 | Some concerns | Low risk | No concerns | No concerns | No concerns | No concerns | High | [] |
| bevacizumab+IVTA:ranibizumab | 0 | Some concerns | Low risk | No concerns | Some concerns | Some concerns | No concerns | Moderate | [] |
| bevacizumab+PDT:brolucizumab | 0 | Some concerns | Low risk | No concerns | Major concerns | No concerns | No concerns | Moderate | ["Imprecision"] |
| bevacizumab+PDT:conbercept | 0 | Some concerns | Low risk | No concerns | No concerns | Some concerns | No concerns | High | [] |
| bevacizumab+PDT:placebo | 0 | Some concerns | Low risk | No concerns | No concerns | No concerns | No concerns | High | [] |
| bevacizumab+PDT:ranibizumab | 0 | Some concerns | Low risk | No concerns | Major concerns | No concerns | No concerns | Moderate | ["Imprecision"] |
| **brolucizumab:conbercept** | **0** | **Some concerns** | **Low risk** | **No concerns** | **No concerns** | **No concerns** | **No concerns** | **High** | **[]** |
| brolucizumab:placebo | 0 | Major concerns | Low risk | No concerns | No concerns | No concerns | No concerns | Moderate | ["Within-study bias"] |
| **brolucizumab:ranibizumab** | **0** | **Major concerns** | **Low risk** | **No concerns** | **Major concerns** | **No concerns** | **No concerns** | **Low** | **["Within-study bias","Imprecision"]** |
| **conbercept:ranibizumab** | **0** | **Some concerns** | **Low risk** | **No concerns** | **No concerns** | **No concerns** | **No concerns** | **High** | **[]** |

# eTable 13: Confidence in Network Meta-Analysis (CINeMA) assessment for the outcome of vision loss

| **Comparison** | **Number of studies** | **Within-study bias** | **Reporting bias** | **Indirectness** | **Imprecision** | **Heterogeneity** | **Incoherence** | **Confidence rating** | **Reason(s) for downgrading** |
| --- | --- | --- | --- | --- | --- | --- | --- | --- | --- |
| DXM+PDT+ranibizumab:PDT+ranibizumab | 1 | Some concerns | Low risk | No concerns | Major concerns | No concerns | No concerns | Moderate | ["Imprecision"] |
| DXM+PDT+ranibizumab:ranibizumab | 1 | Some concerns | Low risk | No concerns | Major concerns | No concerns | No concerns | Moderate | ["Imprecision"] |
| DXM+ranibizumab:ranibizumab | 1 | Major concerns | Low risk | No concerns | Major concerns | No concerns | No concerns | Low | ["Within-study bias","Imprecision"] |
| IVTA:placebo | 1 | No concerns | Low risk | No concerns | Major concerns | No concerns | No concerns | Moderate | ["Imprecision"] |
| IVTA+PDT:PDT | 2 | Some concerns | Low risk | No concerns | Major concerns | No concerns | No concerns | Moderate | ["Imprecision"] |
| aflibercept:PDT | 1 | Major concerns | Low risk | No concerns | No concerns | No concerns | No concerns | Moderate | ["Within-study bias"] |
| PDT:placebo | 4 | Major concerns | Low risk | No concerns | No concerns | No concerns | No concerns | Moderate | ["Within-study bias"] |
| PDT+ranibizumab:ranibizumab | 5 | Some concerns | Low risk | No concerns | Major concerns | No concerns | No concerns | Moderate | ["Imprecision"] |
| **aflibercept:brolucizumab** | **2** | **Major concerns** | **Low risk** | **No concerns** | **Major concerns** | **No concerns** | **No concerns** | **Low** | **["Within-study bias","Imprecision"]** |
| **aflibercept:ranibizumab** | **3** | **Major concerns** | **Low risk** | **No concerns** | **Major concerns** | **No concerns** | **No concerns** | **Low** | **["Within-study bias","Imprecision"]** |
| bevacizumab:bevacizumab+IVTA | 1 | Some concerns | Low risk | No concerns | Major concerns | No concerns | No concerns | Moderate | ["Imprecision"] |
| bevacizumab:bevacizumab+PDT | 3 | Some concerns | Low risk | No concerns | Major concerns | No concerns | No concerns | Moderate | ["Imprecision"] |
| **bevacizumab:ranibizumab** | **10** | **No concerns** | **Low risk** | **No concerns** | **Major concerns** | **No concerns** | **No concerns** | **Moderate** | **["Imprecision"]** |
| conbercept:placebo | 1 | No concerns | Low risk | No concerns | Major concerns | No concerns | No concerns | Moderate | ["Imprecision"] |
| placebo:ranibizumab | 2 | Major concerns | Low risk | No concerns | No concerns | No concerns | No concerns | Moderate | ["Within-study bias"] |
| DXM+PDT+ranibizumab:DXM+ranibizumab | 0 | Some concerns | Low risk | No concerns | Major concerns | No concerns | No concerns | Moderate | ["Imprecision"] |
| DXM+PDT+ranibizumab:IVTA | 0 | Major concerns | Low risk | No concerns | No concerns | No concerns | No concerns | Moderate | ["Within-study bias"] |
| DXM+PDT+ranibizumab:IVTA+PDT | 0 | Some concerns | Low risk | No concerns | Major concerns | No concerns | No concerns | Moderate | ["Imprecision"] |
| DXM+PDT+ranibizumab:PDT | 0 | Major concerns | Low risk | No concerns | No concerns | No concerns | No concerns | Moderate | ["Within-study bias"] |
| aflibercept:DXM+PDT+ranibizumab | 0 | Major concerns | Low risk | No concerns | Major concerns | No concerns | No concerns | Low | ["Within-study bias","Imprecision"] |
| bevacizumab:DXM+PDT+ranibizumab | 0 | Some concerns | Low risk | No concerns | Major concerns | No concerns | No concerns | Moderate | ["Imprecision"] |
| bevacizumab+IVTA:DXM+PDT+ranibizumab | 0 | Some concerns | Low risk | No concerns | Major concerns | No concerns | No concerns | Moderate | ["Imprecision"] |
| bevacizumab+PDT:DXM+PDT+ranibizumab | 0 | Some concerns | Low risk | No concerns | Major concerns | No concerns | No concerns | Moderate | ["Imprecision"] |
| brolucizumab:DXM+PDT+ranibizumab | 0 | Major concerns | Low risk | No concerns | Major concerns | No concerns | No concerns | Low | ["Within-study bias","Imprecision"] |
| conbercept:DXM+PDT+ranibizumab | 0 | Major concerns | Low risk | No concerns | Major concerns | No concerns | No concerns | Low | ["Within-study bias","Imprecision"] |
| DXM+PDT+ranibizumab:placebo | 0 | Some concerns | Low risk | No concerns | No concerns | No concerns | No concerns | High | [] |
| DXM+ranibizumab:IVTA | 0 | Major concerns | Low risk | No concerns | No concerns | No concerns | No concerns | Moderate | ["Within-study bias"] |
| DXM+ranibizumab:IVTA+PDT | 0 | Major concerns | Low risk | No concerns | Major concerns | No concerns | No concerns | Low | ["Within-study bias","Imprecision"] |
| DXM+ranibizumab:PDT | 0 | Major concerns | Low risk | No concerns | No concerns | No concerns | No concerns | Moderate | ["Within-study bias"] |
| DXM+ranibizumab:PDT+ranibizumab | 0 | Major concerns | Low risk | No concerns | Major concerns | No concerns | No concerns | Low | ["Within-study bias","Imprecision"] |
| aflibercept:DXM+ranibizumab | 0 | Major concerns | Low risk | No concerns | Major concerns | No concerns | No concerns | Low | ["Within-study bias","Imprecision"] |
| bevacizumab:DXM+ranibizumab | 0 | Major concerns | Low risk | No concerns | Major concerns | No concerns | No concerns | Low | ["Within-study bias","Imprecision"] |
| bevacizumab+IVTA:DXM+ranibizumab | 0 | Some concerns | Low risk | No concerns | Major concerns | No concerns | No concerns | Moderate | ["Imprecision"] |
| bevacizumab+PDT:DXM+ranibizumab | 0 | Some concerns | Low risk | No concerns | Major concerns | No concerns | No concerns | Moderate | ["Imprecision"] |
| brolucizumab:DXM+ranibizumab | 0 | Major concerns | Low risk | No concerns | Major concerns | No concerns | No concerns | Low | ["Within-study bias","Imprecision"] |
| conbercept:DXM+ranibizumab | 0 | Major concerns | Low risk | No concerns | Major concerns | No concerns | No concerns | Low | ["Within-study bias","Imprecision"] |
| DXM+ranibizumab:placebo | 0 | Major concerns | Low risk | No concerns | No concerns | No concerns | No concerns | Moderate | ["Within-study bias"] |
| IVTA:IVTA+PDT | 0 | Major concerns | Low risk | No concerns | Major concerns | No concerns | No concerns | Low | ["Within-study bias","Imprecision"] |
| IVTA:PDT | 0 | Major concerns | Low risk | No concerns | Major concerns | No concerns | No concerns | Low | ["Within-study bias","Imprecision"] |
| IVTA:PDT+ranibizumab | 0 | Major concerns | Low risk | No concerns | No concerns | No concerns | No concerns | Moderate | ["Within-study bias"] |
| aflibercept:IVTA | 0 | Major concerns | Low risk | No concerns | No concerns | No concerns | No concerns | Moderate | ["Within-study bias"] |
| bevacizumab:IVTA | 0 | No concerns | Low risk | No concerns | No concerns | No concerns | No concerns | High | [] |
| bevacizumab+IVTA:IVTA | 0 | No concerns | Low risk | No concerns | Major concerns | No concerns | No concerns | Moderate | ["Imprecision"] |
| bevacizumab+PDT:IVTA | 0 | No concerns | Low risk | No concerns | No concerns | No concerns | No concerns | High | [] |
| brolucizumab:IVTA | 0 | Major concerns | Low risk | No concerns | No concerns | No concerns | No concerns | Moderate | ["Within-study bias"] |
| conbercept:IVTA | 0 | No concerns | Low risk | No concerns | Major concerns | No concerns | No concerns | Moderate | ["Imprecision"] |
| IVTA:ranibizumab | 0 | Major concerns | Low risk | No concerns | No concerns | No concerns | No concerns | Moderate | ["Within-study bias"] |
| IVTA+PDT:PDT+ranibizumab | 0 | Major concerns | Low risk | No concerns | Major concerns | No concerns | No concerns | Low | ["Within-study bias","Imprecision"] |
| aflibercept:IVTA+PDT | 0 | Major concerns | Low risk | No concerns | Major concerns | No concerns | No concerns | Low | ["Within-study bias","Imprecision"] |
| bevacizumab:IVTA+PDT | 0 | Major concerns | Low risk | No concerns | Major concerns | No concerns | No concerns | Low | ["Within-study bias","Imprecision"] |
| bevacizumab+IVTA:IVTA+PDT | 0 | Some concerns | Low risk | No concerns | Major concerns | No concerns | No concerns | Moderate | ["Imprecision"] |
| bevacizumab+PDT:IVTA+PDT | 0 | Some concerns | Low risk | No concerns | Major concerns | No concerns | No concerns | Moderate | ["Imprecision"] |
| brolucizumab:IVTA+PDT | 0 | Major concerns | Low risk | No concerns | Major concerns | No concerns | No concerns | Low | ["Within-study bias","Imprecision"] |
| conbercept:IVTA+PDT | 0 | Major concerns | Low risk | No concerns | Major concerns | No concerns | No concerns | Low | ["Within-study bias","Imprecision"] |
| IVTA+PDT:placebo | 0 | Major concerns | Low risk | No concerns | No concerns | Some concerns | No concerns | Moderate | ["Within-study bias"] |
| IVTA+PDT:ranibizumab | 0 | Major concerns | Low risk | No concerns | Major concerns | No concerns | No concerns | Low | ["Within-study bias","Imprecision"] |
| PDT:PDT+ranibizumab | 0 | Major concerns | Low risk | No concerns | No concerns | No concerns | No concerns | Moderate | ["Within-study bias"] |
| bevacizumab:PDT | 0 | Major concerns | Low risk | No concerns | No concerns | No concerns | No concerns | Moderate | ["Within-study bias"] |
| bevacizumab+IVTA:PDT | 0 | Major concerns | Low risk | No concerns | Major concerns | No concerns | No concerns | Low | ["Within-study bias","Imprecision"] |
| bevacizumab+PDT:PDT | 0 | Major concerns | Low risk | No concerns | No concerns | No concerns | No concerns | Moderate | ["Within-study bias"] |
| brolucizumab:PDT | 0 | Major concerns | Low risk | No concerns | No concerns | No concerns | No concerns | Moderate | ["Within-study bias"] |
| conbercept:PDT | 0 | Major concerns | Low risk | No concerns | Major concerns | No concerns | No concerns | Low | ["Within-study bias","Imprecision"] |
| PDT:ranibizumab | 0 | Major concerns | Low risk | No concerns | No concerns | No concerns | No concerns | Moderate | ["Within-study bias"] |
| aflibercept:PDT+ranibizumab | 0 | Major concerns | Low risk | No concerns | Major concerns | No concerns | No concerns | Low | ["Within-study bias","Imprecision"] |
| bevacizumab:PDT+ranibizumab | 0 | Some concerns | Low risk | No concerns | Major concerns | No concerns | No concerns | Moderate | ["Imprecision"] |
| bevacizumab+IVTA:PDT+ranibizumab | 0 | Some concerns | Low risk | No concerns | Major concerns | No concerns | No concerns | Moderate | ["Imprecision"] |
| bevacizumab+PDT:PDT+ranibizumab | 0 | Some concerns | Low risk | No concerns | Major concerns | No concerns | No concerns | Moderate | ["Imprecision"] |
| brolucizumab:PDT+ranibizumab | 0 | Major concerns | Low risk | No concerns | Major concerns | No concerns | No concerns | Low | ["Within-study bias","Imprecision"] |
| conbercept:PDT+ranibizumab | 0 | Major concerns | Low risk | No concerns | Major concerns | No concerns | No concerns | Low | ["Within-study bias","Imprecision"] |
| PDT+ranibizumab:placebo | 0 | Major concerns | Low risk | No concerns | No concerns | No concerns | No concerns | Moderate | ["Within-study bias"] |
| **aflibercept:bevacizumab** | **0** | **Major concerns** | **Low risk** | **No concerns** | **Major concerns** | **No concerns** | **No concerns** | **Low** | **["Within-study bias","Imprecision"]** |
| aflibercept:bevacizumab+IVTA | 0 | Some concerns | Low risk | No concerns | Major concerns | No concerns | No concerns | Moderate | ["Imprecision"] |
| aflibercept:bevacizumab+PDT | 0 | Some concerns | Low risk | No concerns | Major concerns | No concerns | No concerns | Moderate | ["Imprecision"] |
| **aflibercept:conbercept** | **0** | **Major concerns** | **Low risk** | **No concerns** | **Major concerns** | **No concerns** | **No concerns** | **Low** | **["Within-study bias","Imprecision"]** |
| aflibercept:placebo | 0 | Major concerns | Low risk | No concerns | No concerns | No concerns | No concerns | Moderate | ["Within-study bias"] |
| **bevacizumab:brolucizumab** | **0** | **Major concerns** | **Low risk** | **No concerns** | **Major concerns** | **No concerns** | **No concerns** | **Low** | **["Within-study bias","Imprecision"]** |
| **bevacizumab:conbercept** | **0** | **No concerns** | **Low risk** | **No concerns** | **Major concerns** | **No concerns** | **No concerns** | **Moderate** | **["Imprecision"]** |
| bevacizumab:placebo | 0 | Major concerns | Low risk | No concerns | No concerns | No concerns | No concerns | Moderate | ["Within-study bias"] |
| bevacizumab+IVTA:bevacizumab+PDT | 0 | Some concerns | Low risk | No concerns | Major concerns | No concerns | No concerns | Moderate | ["Imprecision"] |
| bevacizumab+IVTA:brolucizumab | 0 | Major concerns | Low risk | No concerns | Major concerns | No concerns | No concerns | Low | ["Within-study bias","Imprecision"] |
| bevacizumab+IVTA:conbercept | 0 | No concerns | Low risk | No concerns | Major concerns | No concerns | No concerns | Moderate | ["Imprecision"] |
| bevacizumab+IVTA:placebo | 0 | Some concerns | Low risk | No concerns | Major concerns | No concerns | No concerns | Moderate | ["Imprecision"] |
| bevacizumab+IVTA:ranibizumab | 0 | Some concerns | Low risk | No concerns | Major concerns | No concerns | No concerns | Moderate | ["Imprecision"] |
| bevacizumab+PDT:brolucizumab | 0 | Major concerns | Low risk | No concerns | Major concerns | No concerns | No concerns | Low | ["Within-study bias","Imprecision"] |
| bevacizumab+PDT:conbercept | 0 | No concerns | Low risk | No concerns | Major concerns | No concerns | No concerns | Moderate | ["Imprecision"] |
| bevacizumab+PDT:placebo | 0 | Some concerns | Low risk | No concerns | No concerns | No concerns | No concerns | High | [] |
| bevacizumab+PDT:ranibizumab | 0 | Some concerns | Low risk | No concerns | Major concerns | No concerns | No concerns | Moderate | ["Imprecision"] |
| **brolucizumab:conbercept** | **0** | **Major concerns** | **Low risk** | **No concerns** | **Major concerns** | **No concerns** | **No concerns** | **Low** | **["Within-study bias","Imprecision"]** |
| brolucizumab:placebo | 0 | Major concerns | Low risk | No concerns | No concerns | No concerns | No concerns | Moderate | ["Within-study bias"] |
| **brolucizumab:ranibizumab** | **0** | **Major concerns** | **Low risk** | **No concerns** | **Major concerns** | **No concerns** | **No concerns** | **Low** | **["Within-study bias","Imprecision"]** |
| **conbercept:ranibizumab** | **0** | **Major concerns** | **Low risk** | **No concerns** | **Major concerns** | **No concerns** | **No concerns** | **Low** | **["Within-study bias","Imprecision"]** |

# eFigure 3: Rank Heat Plot


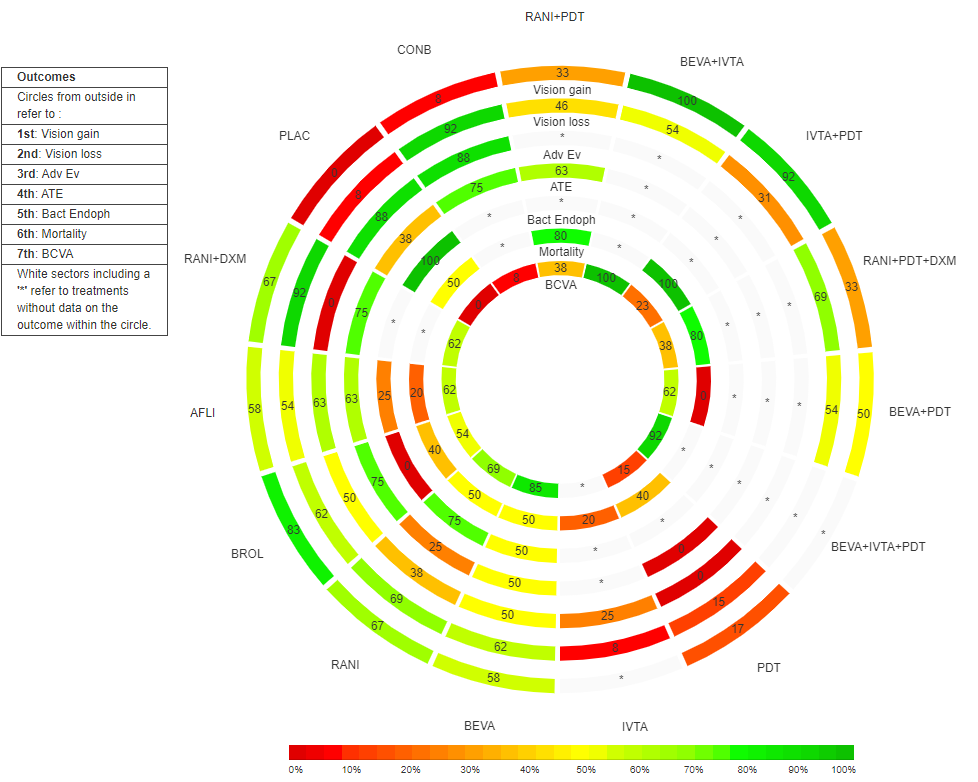


Each concentric circle represents a different outcome (as labeled), with the outermost circle representing vision gain, and the innermost circle representing best-corrected visual acuity. The scale bar represents the ranking statistic for each intervention using the SUCRA values, where 0% (red) indicates the lowest possible rank (worst treatment), and 100% (green) represents the highest possible rank (best treatment). Each rectangle represents an intervention which is described outside the outermost circle. The number within each rectangle represents the ranking statistic of the intervention for the particular outcome circle.

**Abbreviations:** AE, adverse events; AFLI, aflibercept; ATE, arterial thromboembolic events; BCVA, best-corrected visual acuity; BEVA, bevacizumab; BROL, brolucizumab; CONB, conbercept; DXM, dexamethasone; IVTA, intravitreal triamcinolone acetonide; PDT, photodynamic therapy; PLAC, placebo; RANI, ranibizumab; RD, retinal detachment; VTE, venous thromboembolic events; WDAE, withdrawals due to adverse events

# eTable 14: Comparison to Previous Systematic Reviews

|  | This review | Review by Fadda et al.[97] | Solomon et al.[98] |
| --- | --- | --- | --- |
| Number of included studies | 90 | 5 | 16 |
| Included treatments | Aflibercept  Bevacizumab  Bevacizumab+IVTA+PDT  Bevacizumab+PDT  DXM  DXM+PDT+Ranibizumab  DXM+Ranibizumab  IVTA  IVTA+Bevacizumab  IVTA+PDT  IVTA+Ranibizumab  PDT  PDT+Ranibizumab  Ranibizumab  Conbercept  Brolucizumab | Bevacizumab  Pegatanib  Ranibizumab  Verteporfin | Bevacizumab  Pegaptanib  Ranibizumab |

**Abbreviations:** DXM, dexamethasone; IVTA, intravitreal triamcinolone acetonide; PDT, photodynamic therapy

# References

1. Bayer Inc. Eylea - Aflibercept Single Use Vials for the Treatment of a Single Eye 40 mg/mL Solution for Intravitreal Injection Ophthalmological / Antineovascularization agent. 2017; <http://omr.bayer.ca/omr/online/eylea-pm-en.pdf>. Accessed May 2017.

2. Novartis Pharmaceuticals Canada Inc. Lucenits - Anti-Vascular Endothelial Growth Factor-A (VEGF-A inhibitor) For Age-Related Macular Degeneration, Visual Impairment due to Diabetic Macular Edema, Visual Impairment due to Macular Edema Secondary to Retinal Vein Occlusion, and Visual Impairment due to Choroidal Neovascularisation Secondary to Pathologic Myopia Antineovascularisation agent. 2015; <https://www.novartis.ca/sites/www.novartis.ca/files/lucentis_scrip_e.pdf>. Accessed May 2017.

3. Medscape. bevacizumab (Rx). <http://reference.medscape.com/drug/avastin-bevacizumab-342257>. Accessed May 2017.

4. Ahmadieh H, Taei R, Riazi-Esfahani M, et al. Intravitreal bevacizumab versus combined intravitreal bevacizumab and triamcinolone for neovascular age-related macular degeneration: six-month results of a randomized clinical trial. Retina (Philadelphia, Pa). 2011;31(9):1819-1826.

5. Amarakoon S, Martinez-Ciriano JP, van den Born LI, Baarsma S, Missotten T. Bevacizumab in age-related macular degeneration: a randomized controlled trial on the effect of on-demand therapy every 4 or 8 weeks. Acta ophthalmologica. 2019;97(1):107-112.

6. Arias L, Garcia-Arumi J, Ramon JM, Badia M, Rubio M, Pujol O. Photodynamic therapy with intravitreal triamcinolone in predominantly classic choroidal neovascularization: one-year results of a randomized study. Ophthalmology. 2006;113(12):2243-2250.

7. Verteporfin In Photodynamic Therapy Study Group. Verteporfin therapy of subfoveal choroidal neovascularization in age-related macular degeneration: two-year results of a randomized clinical trial including lesions with occult with no classic choroidal neovascularization--verteporfin in photodynamic therapy report 2. Am J Ophthalmol. 2001;131(5):541-560.

8. Azab M, Boyer DS, Bressler NM, et al. Verteporfin therapy of subfoveal minimally classic choroidal neovascularization in age-related macular degeneration: 2-year results of a randomized clinical trial. Arch Ophthalmol. 2005;123(4):448-457.

9. Barikian A, Mahfoud Z, Abdulaal M, Safar A, Bashshur ZF. Induction with intravitreal bevacizumab every two weeks in the management of neovascular age-related macular degeneration. Am J Ophthalmol. 2015;159(1):131-137.

10. Bashshur ZF, Schakal A, Hamam RN, El Haibi CP, Jaafar RF, Noureddin BN. Intravitreal bevacizumab vs verteporfin photodynamic therapy for neovascular age-related macular degeneration. Arch Ophthalmol. 2007;125(10):1357-1361.

11. Berg K, Pedersen TR, Sandvik L, Bragadottir R. Comparison of ranibizumab and bevacizumab for neovascular age-related macular degeneration according to LUCAS treat-and-extend protocol. Ophthalmology. 2015;122(1):146-152.

12. Biswas P, Sengupta S, Choudhary R, Home S, Paul A, Sinha S. Comparing ranibizumab with bevacizumab. Ophthalmology. 2011;118(3):600-600.

13. Biswas P, Sengupta S, Choudhary R, Home S, Paul A, Sinha S. Comparative role of intravitreal ranibizumab versus bevacizumab in choroidal neovascular membrane in age-related macular degeneration. Indian Journal of Ophthalmology. 2011;59(3):191-196.

14. Boyer DS, Heier JS, Brown DM, Francom SF, Ianchulev T, Rubio RG. A phase IIIb study to evaluate the safety of ranibizumab in subjects with neovascular age-related macular degeneration. Ophthalmology. 2009;116(9):1731-1739.

15. Bressler NM. Photodynamic therapy of subfoveal choroidal neovascularization in age-related macular degeneration with verteporfin: two-year results of 2 randomized clinical trials-tap report 2. Arch Ophthalmol. 2001;119(2):198-207.

16. Brown DM, Heier JS, Ciulla T, et al. Primary endpoint results of a phase II study of vascular endothelial growth factor trap-eye in wet age-related macular degeneration. Ophthalmology. 2011;118(6):1089-1097.

17. Heier JS, Boyer D, Nguyen QD, et al. The 1-year results of CLEAR-IT 2, a phase 2 study of vascular endothelial growth factor trap-eye dosed as-needed after 12-week fixed dosing. Ophthalmology. 2011;118(6):1098-1106.

18. Busbee BG, Ho AC, Brown DM, et al. Twelve-month efficacy and safety of 0.5 mg or 2.0 mg ranibizumab in patients with subfoveal neovascular age-related macular degeneration. Ophthalmology. 2013;120(5):1046-1056.

19. Chakravarthy U, Harding SP, Rogers CA, et al. Alternative treatments to inhibit VEGF in age-related choroidal neovascularisation: 2-year findings of the IVAN randomised controlled trial. Lancet. 2013;382(9900):1258-1267.

20. Chan CK, Abraham P, Sarraf D, Nuthi AS, Lin SG, McCannel CA. Earlier therapeutic effects associated with high dose (2.0 mg) Ranibizumab for treatment of vascularized pigment epithelial detachments in age-related macular degeneration. Eye (Lond). 2015;29(1):80-87.

21. Chaudhary V, Mao A, Hooper PL, Sheidow TG. Triamcinolone acetonide as adjunctive treatment to verteporfin in neovascular age-related macular degeneration: a prospective randomized trial. Ophthalmology. 2007;114(12):2183-2189.

22. Chen E, Brown DM, Wong TP, et al. Lucentis using Visudyne study: determining the threshold-dose fluence of verteporfin photodynamic therapy combined with intravitreal ranibizumab for exudative macular degeneration. Clin Ophthalmol. 2010;4:1073-1079.

23. Costagliola C, Romano MR, Rinaldi M, et al. Low fluence rate photodynamic therapy combined with intravitreal bevacizumab for neovascular age-related macular degeneration. Br J Ophthalmol. 2010;94(2):180-184.

24. Danis RP, Ciulla TA, Pratt LM, Anliker W. Intravitreal triamcinolone acetonide in exudative age-related macular degeneration. Retina (Philadelphia, Pa). 2000;20(3):244-250.

25. Datseris I, Kontadakis GA, Diamanti R, et al. Prospective comparison of low-fluence photodynamic therapy combined with intravitreal bevacizumab versus bevacizumab monotherapy for choroidal neovascularization in age-related macular degeneration. Semin Ophthalmol. 2015;30(2):112-117.

26. Dugel PU, Koh A, Ogura Y, et al. HAWK and HARRIER: Phase 3, Multicenter, Randomized, Double-Masked Trials of Brolucizumab for Neovascular Age-Related Macular Degeneration. Ophthalmology. 2020;127(1):72-84.

27. Dunavoelgyi R, Sacu S, Simader C, Pruente C, Schmidt-Erfurth U. Changes in macular sensitivity after reduced fluence photodynamic therapy combined with intravitreal triamcinolone. Acta ophthalmologica. 2011;89(2):166-171.

28. Eldem BM, Muftuoglu G, Topbas S, et al. A randomized trial to compare the safety and efficacy of two ranibizumab dosing regimens in a Turkish cohort of patients with choroidal neovascularization secondary to AMD. Acta ophthalmologica. 2015;93(6):e458-464.

29. El-Mollayess GM, Mahfoud Z, Schakal AR, Salti HI, Jaafar D, Bashshur ZF. Fixed-interval versus OCT-guided variable dosing of intravitreal bevacizumab in the management of neovascular age-related macular degeneration: a 12-month randomized prospective study. Am J Ophthalmol. 2012;153(3):481-489.e481.

30. Feltgen N, Bertelmann T, Bretag M, et al. Efficacy and safety of a fixed bimonthly ranibizumab treatment regimen in eyes with neovascular age-related macular degeneration: results from the RABIMO trial. Graefes Arch Clin Exp Ophthalmol. 2017;255(5):923-934.

31. Fung AT, Kumar N, Vance SK, et al. Pilot study to evaluate the role of high-dose ranibizumab 2.0 mg in the management of neovascular age-related macular degeneration in patients with persistent/recurrent macular fluid <30 days following treatment with intravitreal anti-VEGF therapy (the LAST Study). Eye (Lond). 2012;26(9):1181-1187.

32. Gharbiya M, Giustolisi R, Marchiori J, et al. Comparison of Short-Term Choroidal Thickness and Retinal Morphological Changes after Intravitreal Anti-VEGF Therapy with Ranibizumab or Aflibercept in Treatment-Naive Eyes. Current eye research. 2018;43(3):391-396.

33. Gillies MC, Simpson JM, Luo W, et al. A randomized clinical trial of a single dose of intravitreal triamcinolone acetonide for neovascular age-related macular degeneration: one-year results. Arch Ophthalmol. 2003;121(5):667-673.

34. Gillies MC, Simpson JM, Billson FA, et al. Safety of an intravitreal injection of triamcinolone: results from a randomized clinical trial. Arch Ophthalmol. 2004;122(3):336-340.

35. Gillies MC, Hunyor AP, Arnold JJ, et al. Effect of Ranibizumab and Aflibercept on Best-Corrected Visual Acuity in Treat-and-Extend for Neovascular Age-Related Macular Degeneration: A Randomized Clinical Trial. JAMA ophthalmology. 2019;137(4):372-379.

36. Giustolisi R, Fantozzi N, Staltari M, et al. Combined intravitreal ranibizumab and verteporfin photodynamic therapy versus ranibizumab alone for the treatment of age-related macular degeneration. Digit J Ophthalmol. 2011;17(3):23-30.

37. Guymer RH, Markey CM, McAllister IL, Gillies MC, Hunyor AP, Arnold JJ. Tolerating Subretinal Fluid in Neovascular Age-Related Macular Degeneration Treated with Ranibizumab Using a Treat-and-Extend Regimen: FLUID Study 24-Month Results. Ophthalmology. 2019;126(5):723-734.

38. Haga A, Kawaji T, Ideta R, Inomata Y, Tanihara H. Treat-and-extend versus every-other-month regimens with aflibercept in age-related macular degeneration. Acta ophthalmologica. 2018;96(3):e393-e398.

39. Hatz K, Schneider U, Henrich PB, Braun B, Sacu S, Prunte C. Ranibizumab plus verteporfin photodynamic therapy in neovascular age-related macular degeneration: 12 months of retreatment and vision outcomes from a randomized study. Ophthalmologica Journal international d'ophtalmologie International journal of ophthalmology Zeitschrift fur Augenheilkunde. 2015;233(2):66-73.

40. Heier JS, Brown DM, Chong V, et al. Intravitreal aflibercept (VEGF trap-eye) in wet age-related macular degeneration. Ophthalmology. 2012;119(12):2537-2548.

41. QLT Inc. Reduced Fluence Visudyne-Anti-VEGF-Dexamethasone In Combination for AMD Lesions (RADICAL) (RADICAL). 2011; <https://clinicaltrials.gov/show/NCT00492284>. Accessed December 18, 2020.

42. Kaiser PK. Verteporfin PDT for subfoveal occult CNV in AMD: two-year results of a randomized trial. Curr Med Res Opin. 2009;25(8):1853-1860.

43. Kaiser PK, Boyer DS, Cruess AF, Slakter JS, Pilz S, Weisberger A. Verteporfin plus ranibizumab for choroidal neovascularization in age-related macular degeneration: twelve-month results of the DENALI study. Ophthalmology. 2012;119(5):1001-1010.

44. Kertes PJ, Galic IJ, Greve M, et al. Canadian Treat-and-Extend Analysis Trial with Ranibizumab in Patients with Neovascular Age-Related Macular Disease: One-Year Results of the Randomized Canadian Treat-and-Extend Analysis Trial with Ranibizumab Study. Ophthalmology. 2019;126(6):841-848.

45. Kodjikian L, Souied EH, Mimoun G, et al. Ranibizumab versus bevacizumab for neovascular age-related macular degeneration: results from the GEFAL noninferiority randomized trial. Ophthalmology. 2013;120(11):2300-2309.

46. Krebs I, Schmetterer L, Boltz A, et al. A randomised double-masked trial comparing the visual outcome after treatment with ranibizumab or bevacizumab in patients with neovascular age-related macular degeneration. British Journal of Ophthalmology. 2013;97(3):266-271.

47. Krebs I, Vecsei Marlovits V, Bodenstorfer J, et al. Comparison of Ranibizumab monotherapy versus combination of Ranibizumab with photodynamic therapy with neovascular age-related macular degeneration. Acta ophthalmologica. 2013;91(3):e178-183.

48. Kuppermann BD, Goldstein M, Maturi RK, et al. Dexamethasone Intravitreal Implant as Adjunctive Therapy to Ranibizumab in Neovascular Age-Related Macular Degeneration: A Multicenter Randomized Controlled Trial. Ophthalmologica Journal international d'ophtalmologie International journal of ophthalmology Zeitschrift fur Augenheilkunde. 2015;234(1):40-54.

49. Lai TY, Liu DT, Chan KP, Luk FO, Pang CP, Lam DS. Visual outcomes and growth factor changes of two dosages of intravitreal bevacizumab for neovascular age-related macular degeneration: a randomized, controlled trial. Retina (Philadelphia, Pa). 2009;29(9):1218-1226.

50. Larsen M, Schmidt-Erfurth U, Lanzetta P, et al. Verteporfin plus ranibizumab for choroidal neovascularization in age-related macular degeneration: twelve-month MONT BLANC study results. Ophthalmology. 2012;119(5):992-1000.

51. Lazic R, Gabric N. Verteporfin therapy and intravitreal bevacizumab combined and alone in choroidal neovascularization due to age-related macular degeneration. Ophthalmology. 2007;114(6):1179-1185.

52. Lee J, Freeman WR, Azen SP, Chung EJ, Koh HJ. Prospective, randomized clinical trial of intravitreal triamcinolone treatment of neovascular age-related macular degeneration: one-year results. Retina (Philadelphia, Pa). 2007;27(9):1205-1213.

53. Li X, Hu Y, Sun X, Zhang J, Zhang M. Bevacizumab for neovascular age-related macular degeneration in China. Ophthalmology. 2012;119(10):2087-2093.

54. Intravitreal Aflibercept Versus Photodynamic Therapy in Chinese Patients with Neovascular Age-Related Macular Degeneration: Outcomes of the SIGHT Study. Journal of Ocular Pharmacology and Therapeutics. 2017;33(6):435-444.

55. Lim JI, Niec M, Wong V. One year results of a phase 1 study of the safety and tolerability of combination therapy using sustained release intravitreal triamcinolone acetonide and ranibizumab for subfoveal neovascular AMD. Br J Ophthalmol. 2015;99(5):618-623.

56. Liu K, Song Y, Xu G, et al. Conbercept for Treatment of Neovascular Age-related Macular Degeneration: Results of the Randomized Phase 3 PHOENIX Study. American Journal of Ophthalmology. 2019;197:156-167.

57. Lushchyk T, Amarakoon S, Martinez-Ciriano JP, van den Born LI, Baarsma GS, Missotten T. Bevacizumab in age-related macular degeneration: a randomized controlled trial on the effect of injections every 4 weeks, 6 weeks and 8 weeks. Acta ophthalmologica. 2013;91(6):e456-461.

58. Maberley D. Photodynamic therapy and intravitreal triamcinolone for neovascular age-related macular degeneration: a randomized clinical trial. Ophthalmology. 2009;116(11):2149-2157.e2141.

59. Mahmood S, Roberts SA, Aslam TM, Parkes J, Barugh K, Bishop PN. Routine versus As-Needed Bevacizumab with 12-Weekly Assessment Intervals for Neovascular Age-Related Macular Degeneration: 92-Week Results of the GMAN Trial. Ophthalmology. 2015;122(7):1348-1355.

60. Mantel I, Gianniou C, Dirani A. CONVERSION TO AFLIBERCEPT THERAPY VERSUS CONTINUING WITH RANIBIZUMAB THERAPY FOR NEOVASCULAR AGE-RELATED MACULAR DEGENERATION DEPENDENT ON MONTHLY RANIBIZUMAB TREATMENT. Retina (Philadelphia, Pa). 2016;36(1):53-58.

61. Martin DF, Maguire MG, Ying GS, Grunwald JE, Fine SL, Jaffe GJ. Ranibizumab and bevacizumab for neovascular age-related macular degeneration. New England Journal of Medicine. 2011;364(20):1897-1908.

62. Menon G, Chandran M, Sivaprasad S, Chavan R, Narendran N, Yang Y. Is it necessary to use three mandatory loading doses when commencing therapy for neovascular age-related macular degeneration using bevacizumab? (BeMOc Trial). Eye (Lond). 2013;27(8):959-963.

63. Michels S, Wachtlin J, Gamulescu MA, et al. Comparison of early retreatment with the standard regimen in verteporfin therapy of neovascular age-related macular degeneration. Ophthalmology. 2005;112(12):2070-2075.

64. Modarres M, Naseripour M, Falavarjani KG, Nikeghbali A, Hashemi M, Parvaresh MM. Intravitreal injection of 2.5 mg versus 1.25 mg bevacizumab (Avastin) for treatment of CNV associated with AMD. Retina (Philadelphia, Pa). 2009;29(3):319-324.

65. Mori R, Tanaka K, Haruyama M, Kawamura A, Furuya K, Yuzawa M. Comparison of pro re nata versus Bimonthly Injection of Intravitreal Aflibercept for Typical Neovascular Age-Related Macular Degeneration. Ophthalmologica Journal international d'ophtalmologie International journal of ophthalmology Zeitschrift fur Augenheilkunde. 2017;238(1-2):17-22.

66. Motarjemizadeh Q AN, Abbaszadeh M, Sadrinia V. Intravitreal Bevacizumab with or without Triamcinolone for Wet Age-related Macular Degeneration: Twelve-month Results of a Prospective, Randomized Investigation. Middle East African Journal of Opht 2018 Jan-Mar;25(1):1-7. DOI: 10.4103/meajo.meajo_292_16.

67. Nguyen QD, Campochiaro PA, Shah SM, et al. Evaluation of very high- and very low-dose intravitreal aflibercept in patients with neovascular age-related macular degeneration. J Ocul Pharmacol Ther. 2012;28(6):581-588.

68. Meyer P. Die altersabh„ngige Makuladegeneration. Praxis. 2002;91(3):76-79.

69. Grunwald JE, Daniel E, Huang J, et al. Risk of geographic atrophy in the comparison of age-related macular degeneration treatments trials. Ophthalmology. 2014;121(1):150-161.

70. VEGF Trap-Eye: Investigation of Efficacy and Safety in Chinese Subjects With Wet AMD (Age-Related Macular Degeneration). (2016). Retrieved from <https://clinicaltrials.gov/> (Identification No. NCT01482910). 2016.

71. Jiang HL, Han XW, Zhang SQ, Fang XL, Zhao BJ. Efficacy of intravitreal ranibizumab injection combined with macular grid photocoagulation for diabetic macular edema. International Journal of Ophthalmology. 2015;14(7):1253-1256.

72. Parodi MB, Cascavilla M, Papayannis A, Kontadakis DS, Bandello F, Iacono P. Intravitreal bevacizumab in advanced-stage neovascular age-related macular degeneration with visual acuity lower than 20/200. Arch Ophthalmol. 2012;130(7):934-935.

73. Piermarocchi S, Sartore M, Lo Giudice G, Maritan V, Midena E, Segato T. Combination of photodynamic therapy and intraocular triamcinolone for exudative age-related macular degeneration and long-term chorioretinal macular atrophy. Arch Ophthalmol. 2008;126(10):1367-1374.

74. Piri N, Ahmadieh H, Taei R, et al. Photodynamic Therapy and Intravitreal Bevacizumab with Versus without Triamcinolone for Neovascular Age-related Macular Degeneration; a Randomized Clinical Trial. J Ophthalmic Vis Res. 2014;9(4):469-477.

75. Potter MJ, Claudio CC, Szabo SM. A randomised trial of bevacizumab and reduced light dose photodynamic therapy in age-related macular degeneration: the VIA study. Br J Ophthalmol. 2010;94(2):174-179.

76. Ranchod TM, Ray SK, Daniels SA, Leong CJ, Ting TD, Verne AZ. LuceDex: a prospective study comparing ranibizumab plus dexamethasone combination therapy versus ranibizumab monotherapy for neovascular age-related macular degeneration. Retina (Philadelphia, Pa). 2013;33(8):1600-1604.

77. Regillo CD, Brown DM, Abraham P, et al. Randomized, double-masked, sham-controlled trial of ranibizumab for neovascular age-related macular degeneration: PIER Study year 1. Am J Ophthalmol. 2008;145(2):239-248.

78. Rezar-Dreindl S, Eibenberger K, Buehl W, et al. ROLE OF ADDITIONAL DEXAMETHASONE FOR THE MANAGEMENT OF PERSISTENT OR RECURRENT NEOVASCULAR AGE-RELATED MACULAR DEGENERATION UNDER RANIBIZUMAB TREATMENT. Retina (Philadelphia, Pa). 2017;37(5):962-970.

79. Riazi-Esfahani M, Ahmadieh H, Faghihi H, et al. Intravitreal Bevacizumab versus Combined Bevacizumab and Triamcinolone Acetonide for Neovascular Age-Related Macular Degeneration. J Ophthalmic Vis Res. 2008;3(2):95-101.

80. Rosenfeld PJ, Brown DM, Heier JS, et al. Ranibizumab for neovascular age-related macular degeneration. New England Journal of Medicine. 2006;355(14):1419-1431.

81. Chang TS, Bressler NM, Fine JT, Dolan CM, Ward J, Klesert TR. Improved vision-related function after ranibizumab treatment of neovascular age-related macular degeneration: results of a randomized clinical trial. Archives of Ophthalmology. 2007;125(11):1460-1469.

82. Rosenfeld PJ, Heier JS, Hantsbarger G, Shams N. Tolerability and efficacy of multiple escalating doses of ranibizumab (Lucentis) for neovascular age-related macular degeneration. Ophthalmology. 2006;113(4):623.e621.

83. Rosenfeld PJ, Boyer DS, Bressler NM, et al. Verteporfin therapy of subfoveal occult choroidal neovascularization in AMD using delayed light application: one-year results of the VALIO Study. Am J Ophthalmol. 2007;144(6):970-972.

84. Sacu S, Varga A, Michels S, et al. Reduced fluence versus standard photodynamic therapy in combination with intravitreal triamcinolone: short-term results of a randomised study. Br J Ophthalmol. 2008;92(10):1347-1351.

85. Schauwvlieghe AM, Dijkman G, Hooymans JM, et al. Comparing the Effectiveness of Bevacizumab to Ranibizumab in Patients with Exudative Age-Related Macular Degeneration. The BRAMD Study. PLoS One. 2016;11(5):e0153052.

86. Schmidt-Erfurth U, Sacu S. Randomized multicenter trial of more intense and standard early verteporfin treatment of neovascular age-related macular degeneration. Ophthalmology. 2008;115(1):134-140.

87. Schmidt-Erfurth U, Eldem B, Guymer R, et al. Efficacy and safety of monthly versus quarterly ranibizumab treatment in neovascular age-related macular degeneration: the EXCITE study. Ophthalmology. 2011;118(5):831-839.

88. Scholler A, Richter-Mueksch S, Weingessel B, Vecsei-Marlovits PV. Differences of frequency in administration of ranibizumab and bevacizumab in patients with neovascular AMD. Wien Klin Wochenschr. 2014;126(11-12):355-359.

89. Semeraro F, Russo A, Delcassi L, et al. TREATMENT OF EXUDATIVE AGE-RELATED MACULAR DEGENERATION WITH RANIBIZUMAB COMBINED WITH KETOROLAC EYEDROPS OR PHOTODYNAMIC THERAPY. Retina (Philadelphia, Pa). 2015;35(8):1547-1554.

90. Silva R, Berta A, Larsen M, Macfadden W, Feller C, Monés J. Treat-and-Extend versus Monthly Regimen in Neovascular Age-Related Macular Degeneration: Results with Ranibizumab from the TREND Study. Ophthalmology. 2018;125(1):57-65.

91. Subramanian ML, Abedi G, Ness S, et al. Bevacizumab vs ranibizumab for age-related macular degeneration: 1-year outcomes of a prospective, double-masked randomised clinical trial. Eye (Lond). 2010;24(11):1708-1715.

92. Tano Y, Ohji M. EXTEND-I: safety and efficacy of ranibizumab in Japanese patients with subfoveal choroidal neovascularization secondary to age-related macular degeneration. Acta ophthalmologica. 2010;88(3):309-316.

93. Vallance JH, Johnson B, Majid MA, Banerjee S, Mandal K, Bailey CC. A randomised prospective double-masked exploratory study comparing combination photodynamic treatment and intravitreal ranibizumab vs intravitreal ranibizumab monotherapy in the treatment of neovascular age-related macular degeneration. Eye (Lond). 2010;24(10):1561-1567.

94. Weingessel B, Mihaltz K, Vecsei-Marlovits PV. Predictors of 1-year visual outcome in OCT analysis comparing ranibizumab monotherapy versus combination therapy with PDT in exsudative age-related macular degeneration. Wien Klin Wochenschr. 2016;128(15-16):560-565.

95. Williams PD, Callanan D, Solley W, Avery RL, Pieramici DJ, Aaberg T. A prospective pilot study comparing combined intravitreal ranibizumab and half-fluence photodynamic therapy with ranibizumab monotherapy in the treatment of neovascular age-related macular degeneration. Clin Ophthalmol. 2012;6:1519-1525.

96. Wykoff CC, Croft DE, Brown DM, et al. Prospective Trial of Treat-and-Extend versus Monthly Dosing for Neovascular Age-Related Macular Degeneration: TREX-AMD 1-Year Results. Ophthalmology. 2015;122(12):2514-2522.

97. Fadda V, Maratea D, Trippoli S, Messori A. Treatments for macular degeneration: summarising evidence using network meta-analysis. Br J Ophthalmol. 2011;95(10):1476-1477.

98. Solomon SD, Lindsley K, Vedula SS, Krzystolik MG, Hawkins BS. Anti-vascular endothelial growth factor for neovascular age-related macular degeneration. The Cochrane database of systematic reviews. 2019;3(3):Cd005139.
